# Replacement of Less-Preferred Dipolar Aprotic and Ethereal Solvents in Synthetic Organic Chemistry with More Sustainable Alternatives

**DOI:** 10.1021/acs.chemrev.1c00672

**Published:** 2022-02-24

**Authors:** Andrew Jordan, Callum G. J. Hall, Lee R. Thorp, Helen F. Sneddon

**Affiliations:** † School of Chemistry, University of Nottingham, GlaxoSmithKline Carbon Neutral Laboratory, 6 Triumph Road, Nottingham, NG7 2GA, U.K.; ‡ Department of Pure and Applied Chemistry, WestCHEM, University of Strathclyde, Glasgow, Scotland G1 1XL, U.K.; § GlaxoSmithKline Medicines Research Centre, Gunnels Wood Road, Stevenage, Hertfordshire SG1 2NY, U.K.; ⊥ Green Chemistry Centre of Excellence, University of York, Department of Chemistry, University of York, Heslington, York YO10 5DD, U.K.

## Abstract

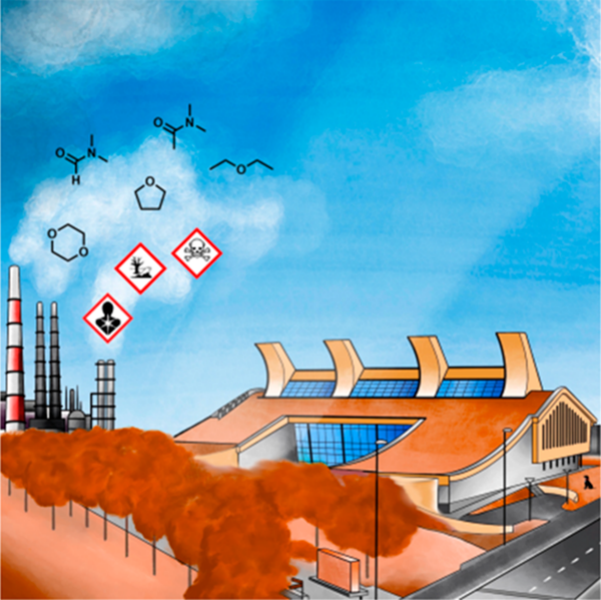

Dipolar aprotic and ethereal solvents comprise just over 40% of all organic solvents utilized in synthetic organic, medicinal, and process chemistry. Unfortunately, many of the common “go-to” solvents are considered to be “less-preferable” for a number of environmental, health, and safety (EHS) reasons such as toxicity, mutagenicity, carcinogenicity, or for practical handling reasons such as flammability and volatility. Recent legislative changes have initiated the implementation of restrictions on the use of many of the commonly employed dipolar aprotic solvents such as dimethylformamide (DMF) and *N*-methyl-2-pyrrolidinone (NMP), and for ethers such as 1,4-dioxane. Thus, with growing legislative, EHS, and societal pressures, the need to identify and implement the use of alternative solvents that are greener, safer, and more sustainable has never been greater. Within this review, the ubiquitous nature of dipolar aprotic and ethereal solvents is discussed with respect to the physicochemical properties that have made them so appealing to synthetic chemists. An overview of the current legislative restrictions being imposed on the use of dipolar aprotic and ethereal solvents is discussed. A variety of alternative, safer, and more sustainable solvents that have garnered attention over the past decade are then examined, and case studies and examples where less-preferable solvents have been successfully replaced with a safer and more sustainable alternative are highlighted. Finally, a general overview and guidance for solvent selection and replacement are included in the Supporting Information of this review.

## 1 Introduction

Solvents are an integral component within many industries, perhaps most significantly in the paints and coatings sector, which makes up for ∼46% of global solvent use. The next largest sector is the pharmaceutical industry comprising 9% of global solvent usage, closely followed by the adhesives (6%), printer inks (6%), and cosmetics (6%) industries. With respect to the pharmaceutical industry, organic solvent use can account for up to 56% of the entire mass involved in the synthesis of an active pharmaceutical ingredient (API).^1^ If water is included in this same calculation, then up to 88% of the mass of an entire process can be attributed to solvent.^1^ Many traditional solvents employed in organic and medicinal chemistry have associated issues such as toxicity, environmental, sustainability, and safety concerns.^2,3^ Thus, reducing the volume of solvent used and using safer and more sustainable solvents are two key concepts that are in agreement with a number of the 12 principles of green chemistry.^4^ It is the intention of this review to present credible, greener, safer, and more sustainable alternatives to a number of dipolar aprotic and ethereal solvents with known issues that are to the best of current knowledge, safer. Case studies that highlight the successful replacement of problematic solvents in commonly employed synthetic organic chemistry with “greener”, more sustainable solvents will also be discussed.

### 1.1 Dipolar Aprotic Solvents: Non Hydrogen Bond Donating Solvents

**Figure 1:**
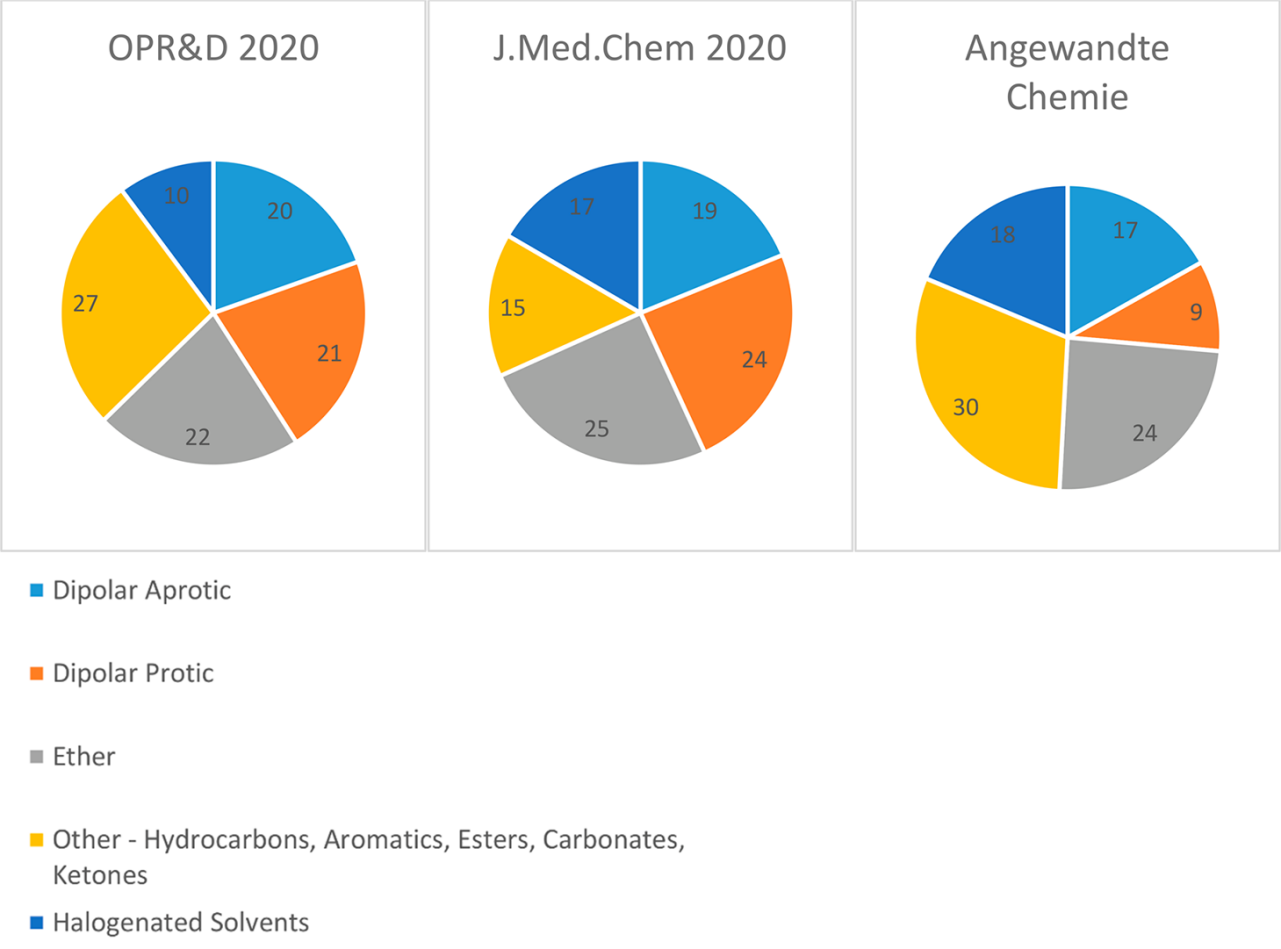
Solvent usage categories across three representative organic chemistry journals, 2020. Percent solvent use included as data label.

Dipolar aprotic solvents are ubiquitous in organic chemistry due to their ability to dissolve a wide variety of materials (often including salts), their versatility as reaction media, and often low cost (e.g., anhydrous DMF could recently be purchased for £46.60 for 1 L (>99% purity GC, ≤ 0.1% water)^5^ and anhydrous 1,4-dioxane could be purchased for £90.40 for 1 L (>99.8%)).^6^ In bulk quantities (not anhydrous), both solvents can be obtained for as little as £0.01/mL.^7^ Another key factor that encourages their widespread use includes the abundance and precedence of existing literature supporting their use in myriad transformations and processes. Dipolar aprotic solvents, such as dimethylformamide (DMF), dimethyl sulfoxide (DMSO), *N*-methyl-2-pyrrolidinone (NMP), acetonitrile (CH_3_CN), acetone, and tetrahydrofuran (THF) are regularly encountered as reaction solvents in chemical transformations. A survey of research published in *Organic Process Research and Development* (*OPR&D*) showed that the most popular dipolar aprotic solvents are DMF, acetonitrile, DMSO, and NMP, in that order.^8^ This is not a significant change from the order reported for the period of 2007–2012 other than DMF and acetonitrile swapping places. DMAc was not listed in the top 25 solvents employed for 2019.^8^ Uptake of dimethyl carbonate was however noted and is a promising sign that more sustainable solvents are being considered as viable alternatives.^9^ A similar analysis was performed for 2020 and analyzed solvent usage trends in three representative journals: *OPR&D*, the *Journal of Medicinal Chemistry*, and *Angewandte Chemie*. Across each of the three distinct journals for the period of 2020, ethereal solvents feature prominently comprised 22–25% of all solvents employed. Dipolar aprotic solvents comprised 17–20% of all solvents used with each class and were similarly represented in each journal, see Figure 1. See the Supporting Information for full solvent usage analysis. It is the composition of each of these classes that is of further interest.

**Figure 2:**
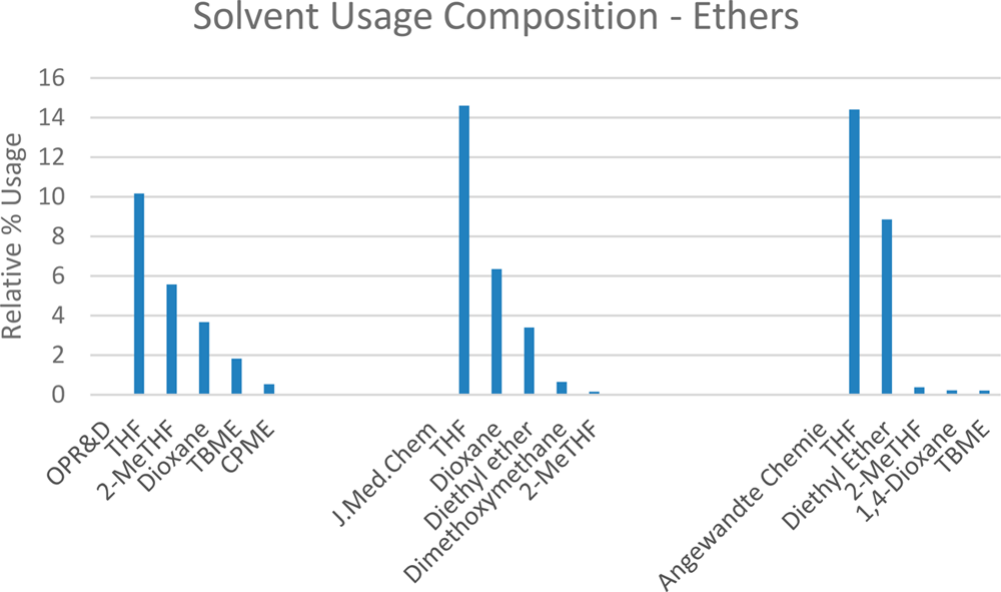
Percentage solvent composition of the ether class.

A closer analysis of each of the solvent categories shows the differences between the particular ethers and dipolar aprotics that are employed in each of the three journals’ publications. THF remains the most popular ethereal solvent across all three data sets. Diethyl ether, with its associated flammability hazards rendering it particularly unfavorable for large scale work, does not feature in the top 25 solvents employed in *OPR&D* in 2020, see Figure 2. Furthermore, it can be seen that alternative ethers such as 2-MeTHF feature far more prominently in *OPR&D* publications. Of note is that 1,4-dioxane does not appear as frequently in publications in *Angewandte Chemie* and we surmise that this is due to the reliance on 1,4-dioxane for cross-coupling in medicinal chemistry C–C bond forming reactions, and Boc protecting group removal.

**Figure 3:**
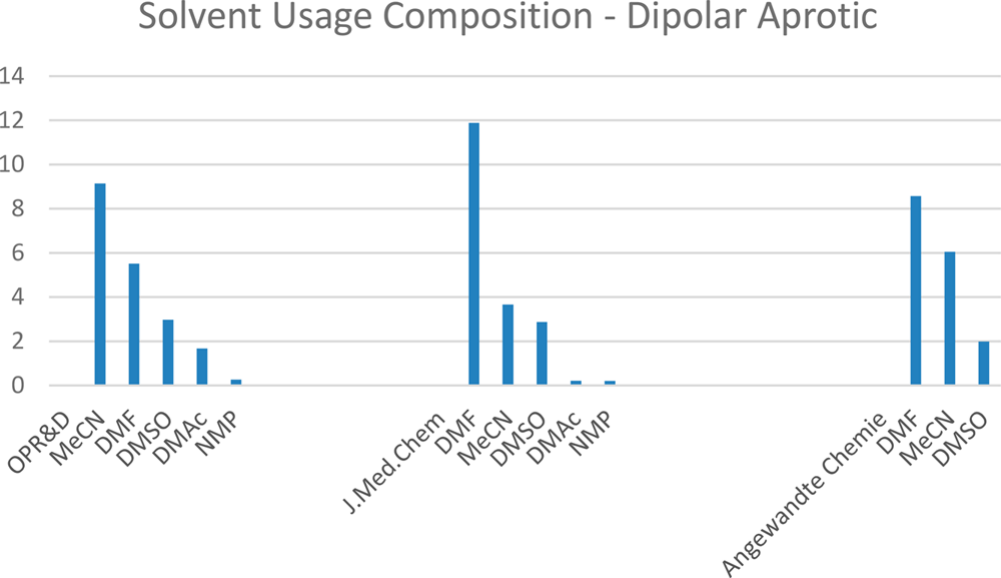
Percentage solvent composition of the dipolar aprotic class.

When the same analysis is applied to the dipolar aprotic class of solvents, it can be seen that publications in the process-scale focused journal *OPR&D* employed CH_3_CN as the most common dipolar aprotic. This is in contrast to both *J. Med. Chem.* and *Angewandte Chemie* in which DMF is the most prominent, see Figure 3. One potential reason for this trend may be that solvent selection in process chemistry and scale-up operations involves far more in-depth risk assessment, which may preclude DMF from use. This may be due to either physicochemical incompatibilities, for example, DMF is known to be incompatible with sodium hydride,^10^ or due to regulatory concerns, which are described in the following section.

Nearly half of all dipolar aprotic solvent usages (with the exception of CH_3_CN) can be attributed to nucleophilic substitution reactions (S_N_2 and S_N_Ar).^9^ Dipolar aprotic solvents are favored for S_N_2 reactions as they do not hydrogen bond to nucleophiles as would a protic solvent, thus promoting the rate of reaction.^11,12^ Use of dipolar aprotic solvents has also long been known to have a potentially enormous rate enhancing effect for S_N_Ar reactions.^13^ 1,4-Dioxane, THF, NMP, and DMF are all considered “go-to” solvents in palladium-catalyzed cross-coupling reactions.^14,15^ The popularity of the less polar ethereal solvents such as 1,4-dioxane and THF is in part due to their ability to coordinate reactive species via hydrogen bond accepting capabilities and Lewis basic character.^15^ Labeling solvents as “aprotic” has long been challenged,^16^ as a number of what are widely considered “aprotic” solvents are in fact incompatible with very strong bases. Therefore, the terms “dipolar non-hydroxylic” and “dipolar non-hydrogen-bond donor” can also be found in the literature.^16^ For all their aforementioned advantages, the majority of commonly employed dipolar aprotic and ethereal solvents pose significant concerns from an environmental, health, and safety perspective (EHS), the reasons for which are discussed in the next section.

### 1.2 Driving Change: Environmental Health and Safety Concerns

The drive to find safer and more sustainable alternatives to dipolar aprotic solvents within the pharmaceutical industry has been a key activity for many years, and it was highlighted in 2007 as a key research area by the ACS Green Chemistry Institute Pharmaceutical Roundtable (ACS GCI PR).^3^ The primary motivating force for identifying alternatives for dipolar aprotic solvents is due to the serious health concerns posed by these solvents such as reproductive toxicity (DMF, DMAc, NMP).^2,17−19^ These concerns have also been flagged under European Union Registration, Evaluation, Authorization, and Restriction of Chemicals (REACH) legislation, categorizing DMF, DMAc, and NMP as “substances of very high concern” (SVHC).^2,17−19^ The authorization process helps to ensure that SVHCs are eventually replaced with less hazardous substances where “technically and economically feasible alternatives are available”.^20^ Restrictions differ in that they are limits or bans on the use of a substance due to unacceptable environmental health and safety risks.^21^ NMP is restricted for use under REACH Annex XVII (substances for which certain uses are Restricted)^22^ due to its reproductive toxicity.^17^ Annex XVII restrictions as of May 2020 limit the sale of NMP on the marketplace as solutions of concentration >0.3%, unless relevant chemical safety reports, data sheets, risk management, and appropriate operating conditions for downstream workers are provided.^22^ DMF and DMAc have also been placed on the candidate list of substances of very high concern;^23^ the intention of the candidate list is for further restrictions on use to be assessed for REACH Annex XVII restrictions.^23^ Similarly, DMF will be restricted from use from December 2023; specifically DMF “shall not be placed on the market as a substance on its own, as a constituent of other substances, or in mixtures in a concentration equal to or greater than 0.3% after 12 December 2023 unless manufacturers, importers and downstream users have included in the relevant chemical safety reports and safety data sheets, Derived No-Effect Levels (DNELs) relating to exposure of workers of 6 mg/m^3^ for exposure by inhalation and 1.1 mg/kg/day for dermal exposure.”^24^ Thus, the need to seek alternatives to DMF is now absolutely critical. Thermal stability and potential safety hazards associated with the use of DMF as a reaction solvent have also recently been reviewed by Yang et al., highlighting a multitude of potential thermal safety issues including thermal runaway and explosive decomposition.^25^

1,4-Dioxane is listed on the US Agency for Toxic Substances and Disease Registry (ATSDR), a repository of hazardous substances and health effects.^26^ Both long- and short-term exposure effects are listed as possible including severe kidney and liver damage and carcinogenicity.^26^ As of July 2021, the European Chemicals Agency (ECHA) has added 1,4-dioxane to its “Candidate List of Substances of Very High Concern for Authorization”^27^ due to its carcinogenic properties and probable serious effects to the environment and human health, see Table 1. Thus, 1,4-dioxane is considered a “substance of very high concern” (SVHC).^23^

**Table 1:**
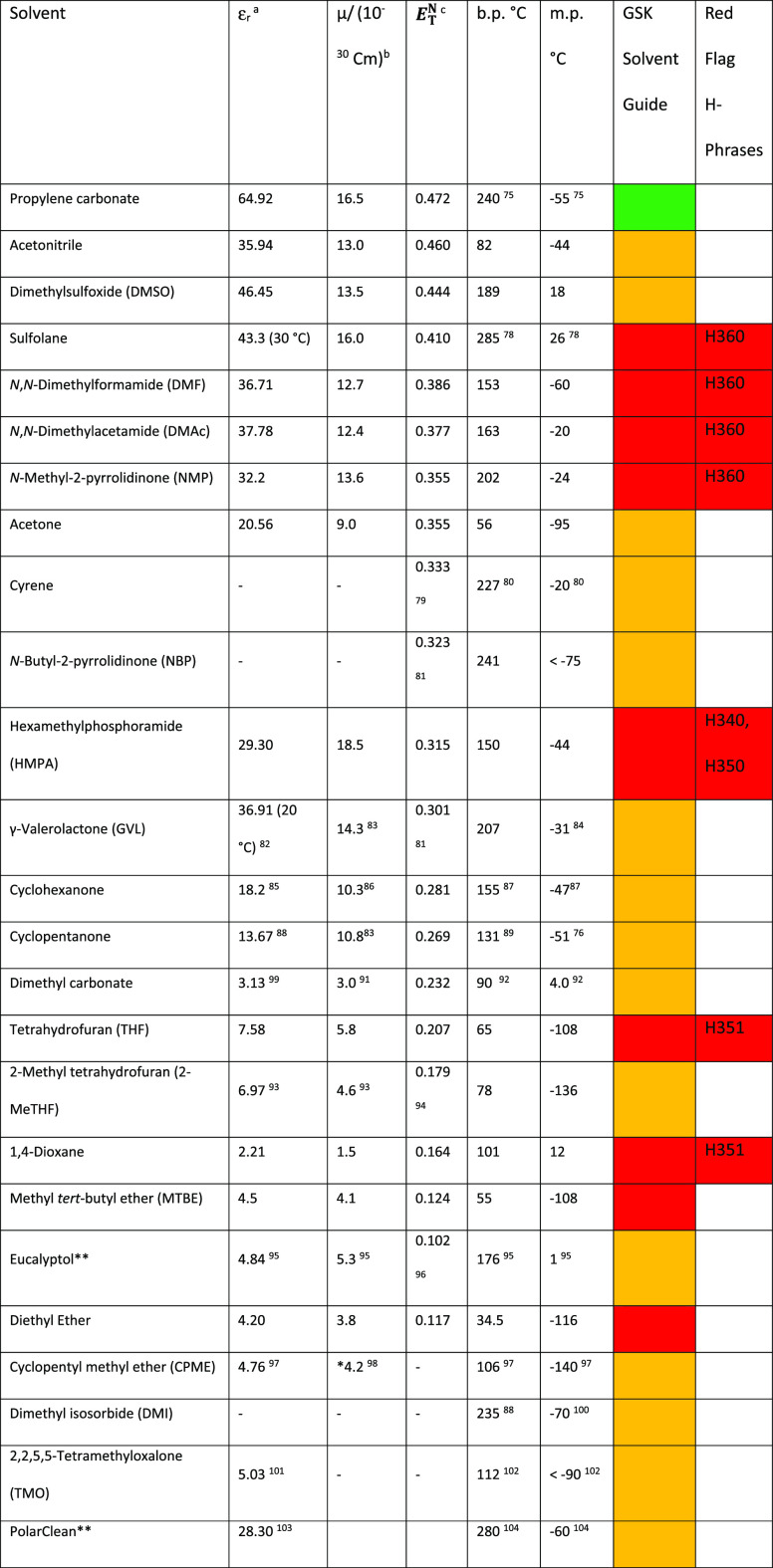
Selected Physical Properties of Some Dipolar Aprotic Solvents^76,79−105^

a Relative permittivity, a.k.a. dielectric constant, of the pure liquid at 25 °C unless stated otherwise.

b Dipole moment in Coulombmeter (Cm).

c Normalized Reichardt polarity parameter measured solvatochromically.^71,73^ Refs from (71) unless noted otherwise. Melting point (m.p.), boiling point (b.p.) from ref (74) unless noted otherwise.^74−77^

d Calculated. Solvents arranged in order of decreasing *E*_*T*_^*N*^.

e Solvents not part of original publication but scored according to published methodology. Solvents are color coded according to a traffic light system where green = solvents with few issues, amber = solvents with some issues, and red = solvents with major issues.^44^ Categorization of dipolar aprotic and ethereal solvents by H-phrases according to the work of McElroy et al.^78^ Note: Hazard phrases are those according to the “Harmonized Classification and Labeling” system as approved by the EU. See the Supporting Information (Unified Solvent Selection Guide for Dipolar Aprotic Solvents) Table S2 for a more in-depth analysis of solvent H-phrase and occupational exposure limits.

By 2023, the limit for 1,4-dioxane in personal care and cleaning products in the state of New York will be reduced to just 1 ppm, with the expectation that similar laws will spread nationwide.^28^ THF is also believed to be a suspect carcinogen, see Table 1. 1,4-Dioxane, diethyl ether, and THF are also known to be peroxide forming solvents and are categorized as “Group B: Peroxide hazards on concentration” as noted in the review of safety guidelines for peroxidizable chemicals by Kelly.^29^ The hazards present when distilling group B solvents arise from the fact that the peroxides formed are often less volatile than the solvent being distilled; thus, the peroxide concentration in the distillation flask can increase to dangerous levels as the operation proceeds. The formation of peroxides can lead to potential explosions^30^ and can be triggered by friction from ground glass stoppers, leading to injury and even fatality.^31^ Peroxide formation can however be mitigated by radical scavengers and stabilizers such as BHT.^32^ Issues associated with the use of diethyl ether, aside from peroxide formation, include high volatility,^33^ low flash point,^33^ flammability,^34^ and explosivity.^30^ Furthermore, diethyl ether has formerly been utilized as an anesthetic^36^ and has been known as a substance with a potential for recreational abuse as early as the 1800s.^37^ Other less commonly employed ethers such as 1,2-dimethoxyethane are also known to be flammable, acutely toxic by inhalation, and to show reproductive toxicity.^38^

With traditionally employed dipolar aprotic solvents coming under increased scrutiny by legislative bodies, a time may come in the not so distant future that sees their use severely curtailed. Such a situation (albeit not for a dipolar aprotic solvent) has already been observed for halogenated solvent dichloroethane (DCE).^39^ Special authorization is now required for the use of DCE on an industrial scale. Furthermore, the permit, once granted, is only valid for 4–12 years, on a case-dependent basis. The short-term nature of the permits is intended to drive the chemical industry to find alternative solvents for DCE, which is a go-to solvent for much of the current pioneering academic research in C–H bond activation.^39^

Other factors that require consideration, outside of direct legislative concerns, are how to dispose of dipolar aprotic solvents postreaction. Often, boiling points of dipolar aprotic solvents are elevated, see Table 1, making distillation and recovery challenging on scale, or indeed so energy intensive as to preclude this method of recovery.^2^ Instead, removal from reaction mixtures can be achieved by aqueous workup. The mixed aqueous–organic waste must then be safely and responsibly disposed of, for example, by incineration unless biological wastewater treatment has been shown to be possible.

To overcome the challenges posed by the changing legislative landscape, and to answer the direct call from industry to find alternative solvents, pioneering research has been conducted over the past ten years. Considerable progress in identifying alternative solvents and reaction conditions for traditional dipolar aprotic solvents has been made, and examples and case studies will be discussed in the next section.

### 1.3 Recent Developments in Greener, More Sustainable Solvents and Reaction Methodology

Recent years have seen a flurry of activity to explore novel bioderived solvents. The major driving forces for such interest, which are described in the previous section, include legislative pressure, pressure to improve industrial sustainability, reduction in global carbon footprint, and a move away from petrochemical derived products toward those that are sustainably sourced. Many of these goals are encompassed by the UN Sustainable Development Goals, for example, Goal 12: “Ensure sustainable consumption and production patterns”^40^ and Goal 13: “Take urgent action to combat climate change and its impacts”.^41^

It should be noted that the source of a solvent is just one aspect of its lifecycle. The energy, water, and reagents, etc. used in the production of the solvent should also be considered, along with its disposal, that is, a life cycle assessment.^42^ Separate from a full LCA (where this information is available), other aspects of a solvent’s overall “greenness” that might be more readily assessed include its biodegradability, volatility, and toxicity. Multiple pharmaceutical companies, universities, and research institutions have attempted to score these attributes using multiparameter chemometric analysis, and a number of solvent selection guides have been produced including guides from Chem21,^43^ GSK,^44^ Sanofi,^45^ AstraZeneca (AZ),^46^ Pfizer,^47^ and the ACS GCI PR.^48^ Furthermore, computational tools^49,50^ and machine-learning^51^ have also been investigated to assist in the solvent selection process. Other industries outside the realms of synthetic chemistry, such as printed electronics, are also developing new tools and methods in solvent selection for their selected specific needs and applications.^52^

Over a decade has passed since the call for finding replacement dipolar aprotic solvents was made by the ACS GCI PR.^3^ In the intervening years, a number of key advances have been made to tackle this challenge.^2^ Developments in the area of greener and more sustainable solvents are regularly highlighted in the series “Green Chemistry Articles of Interest to the Pharmaceutical Industry” published a number of times per year by *Organic Process Research and Development* (*OPR&D*) since 2008.^53^

Original and inventive research utilizing surfactant–water chemistry, such as that championed by Handa et al. and Lipshutz et al., is challenging the very concept of employing organic solvents as reaction media. Instead, it is suggested that significantly important transformations employed in medicinal chemistry, such as amide bond formation and metal catalyzed cross-coupling, can be conducted in surfactant–water systems.^15,54−59^ Key developments have also been made in identifying and discovering new dipolar aprotic solvents with improved safety, sustainability, and green chemistry criteria such as *N*-butyl-2-pyrrolidinone, propylene carbonate, dimethyl isosorbide, and dihydrolevoglucosenone (Cyrene).^2^ Recently, new candidates such as diformylxylose have also been proposed as a replacements for traditional dipolar aprotics, though no toxicity studies have yet been conducted on this solvent.^60^ Also of significance is the growing body of literature employing these alternative reaction solvents and conditions. The growth of literature adopting greener, more sustainable solvents is reinforcing the position that these replacement solvents are genuinely an improvement on the current state of the art and are a credible alternative to the current status quo. EHS evaluations of these new solvents are also ongoing to avoid the potential situation that a replacement solvent could pose toxic effects to the end user or detrimental effects to the environment.^61,62^ Avoiding the promotion of solvents that are just as bad or worse for the user/environment has long been considered^63^ and remains critically important today.

In the following section, some of the recent advances made in identifying, to the best of our knowledge, more preferable, alternative solvents for dipolar aprotic and ethereal solvents are discussed in conjunction with their production, physical, and chemical properties. It is the intention of the authors that these examples can be used as literature case studies and for inspiration and guidance in making judicious solvent selection in chemical processes. More esoteric technologies such as deep eutectic solvents and ionic liquids have purposely been omitted as they are topics requiring reviews of their own. A recently published review by Gao et al. critically discusses replacement strategies for dipolar aprotic solvents and includes both topics. We point the reader toward this review for further information.^64^

#### 1.3.1 Physicochemical Properties of Dipolar Aprotic and Ethereal Solvents

**Figure 4:**
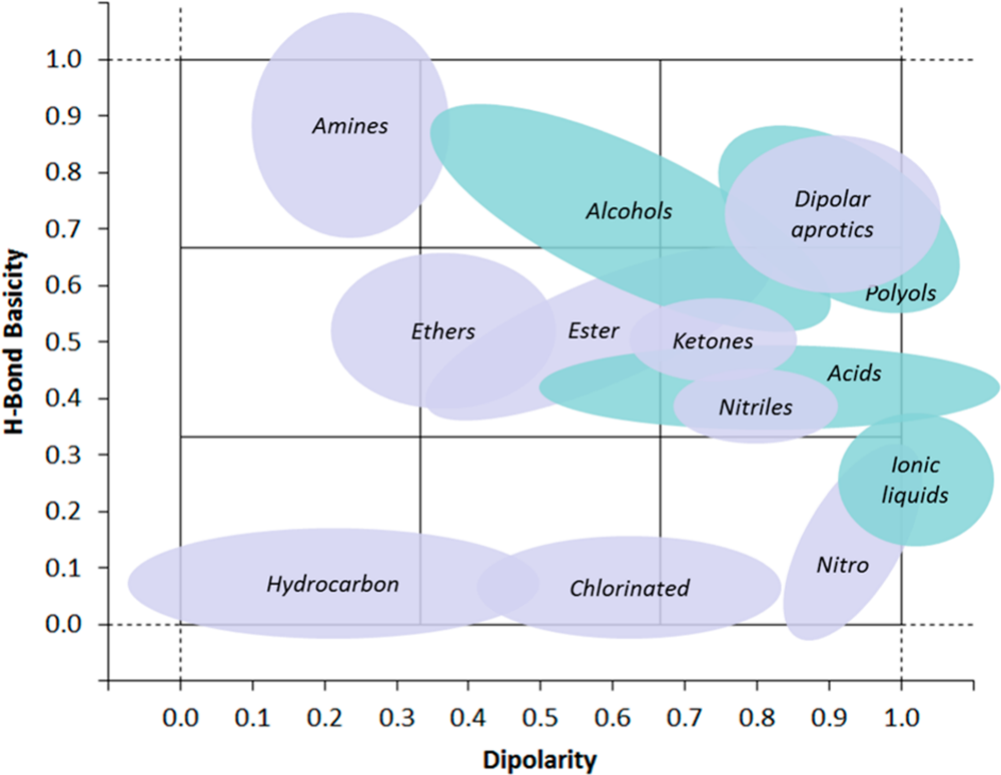
Kamlet–Taft H-bond basicity (β) plotted versus dipolarity (π*) for aprotic solvents (in purple) and protic solvents (in green). Reprinted with permission from ref (66). Copyright 2016 John Wiley and Sons.

Tables of physicochemical properties, Hansen Solubility Parameters (HSP) and Kamlet–Taft (KT) parameters, have been included as both a reference guide and to facilitate the comparison and contrast of various solvents of interest. Plotting HSP parameters in a 3-dimensional space or sphere gives what is known as the “Hansen space”.^65^ Solvents that are close in Hansen space can often exhibit similar solvency power. For example, solvents that were within 3 MPa^1/2^ of each other were identified as potential NMP and DCM replacements by Pacheco et al.^66^ Chemometric tools have also been developed to explore the Hansen space and to identify sets of greener solvents that could potentially be screened for their solubilizing properties.^67^ HSP values can also be calculated for solutes; the closer a solute’s HSP profile is to a known solvent, the more likely it is to dissolve in that solvent. HSP values for solvent blends can also be predicted predicted.^68−70^ Kamlet–Taft parameters describe linear free-energy relationships and are useful for correlating solvent properties with reaction kinetics and processes in equilibrium.^71^ Dipolar aprotic solvents characteristically exhibit no hydrogen bond acidity (HBA) α with the exception of acetone (0.06) and acetonitrile (0.19). Kamlet–Taft parameters can be plotted against each other to produce solvent maps that highlight areas of similarity and difference between solvents and solvent classes, for example, see Figure 4. Methodology for replacing dipolar aprotic solvents by using Kamlet–Taft parameters has been described by Duereh et al.^72^

## 2 Selection of Alternative Solvents

**Figure 5:**
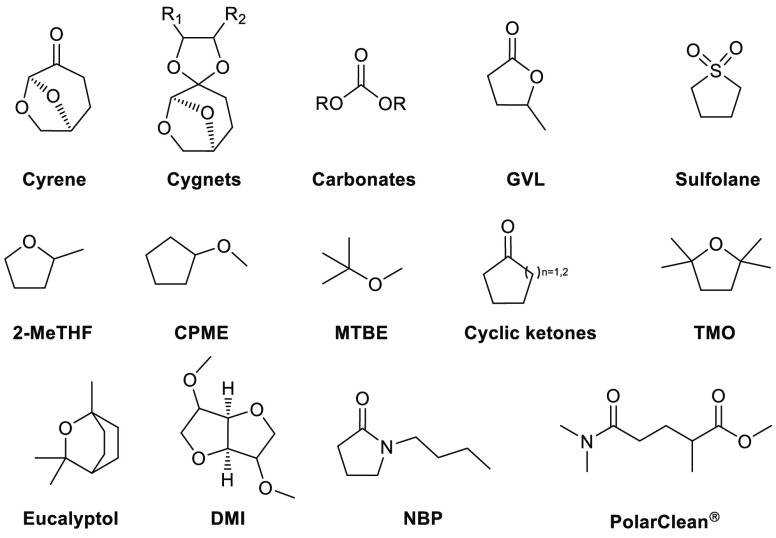
Selection of proposed, greener, safer, more sustainable solvents.

In this section, a selection of solvents that have been promoted as greener and more sustainable alternatives to traditional dipolar aprotic and ethereal solvents will be discussed (Figure 5). An overview of their key features and physicochemical properties that have made them attractive alternatives will be highlighted. Recent reclassification of the “greenness” of certain solvents such as sulfolane (due to reproductive toxicity effects) are also noted. Specific examples and case studies highlighting successful solvent replacements using these solvents will be discussed later.

### 2.1 Cyrene

**Figure 6:**
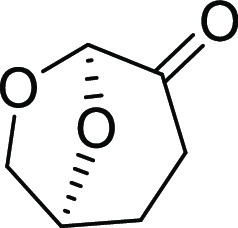
Bio-based solvent “Cyrene”.

**Scheme 1:**
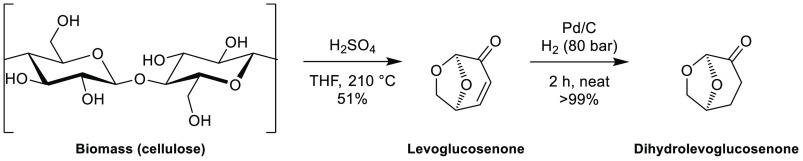
Synthesis of Cyrene from Biomass^118^

Cyrene, or dihydrolevoglucosenone, see Figure 6, is a dipolar aprotic solvent that was designed as a potential alternative to common REACH-restricted solvents such as NMP and DMF.^80^ It is produced from renewable resources (accessed from cellulose in two steps, Scheme 1), can be considered as readily biodegradable, possesses low acute oral toxicity, and has not demonstrated mutagenicity by Ames testing. Ocular irritation has however been reported.^118^ The Kamlet–Taft and HSP parameters for Cyrene were calculated, and it was shown to occupy a similar Hansen solvent space to NMP, see Table 2.^80^

**Table 2:**
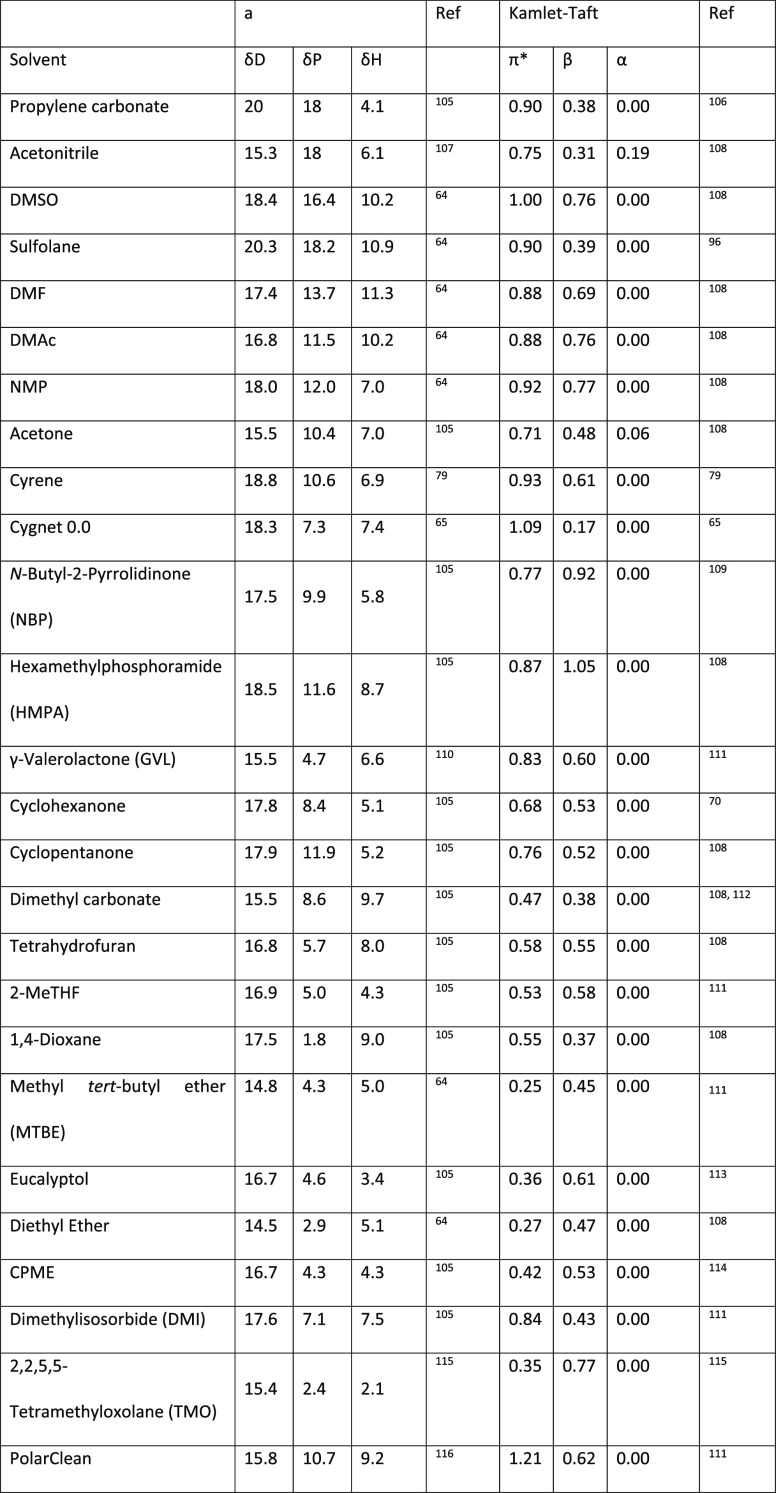
HSP Values (Left) and Kamlet-Taft Parameters (Right) For Some Selected Dipolar Aprotic and Ethereal Solvents^a^^,^^68,106,108,109,111−115,117^

a HSP: van der Waals forces (δD), polarity (δP), and hydrogen bonding (δH). Kamlet–Taft: Dipolarity/polarizability (π*), hydrogen-bonding acidity (donor) (α), and hydrogen-bonding basicity (acceptor) (β). Solvent ordered by empirical solvent polarity, as in Table 1.

Cyrene has been successfully employed as a solvent in multiple transformations including palladium-catalyzed cross-coupling reactions,^14^ acid chloride and amide synthesis,^119,120^ urea synthesis,^121^ MOF synthesis,^122^ and fabrication of water filtration membranes.^123,124^ For a review of Cyrene production and utilization, see Camp et al.^125^ The solubility of solutes in Cyrene has been shown to be dependent on the water content of Cyrene.^126^ An equilibrium between Cyrene’s ketone and a geminal diol form exists. Maximum solubility of solutes was found to be associated with the presence of the diol form. These observations have further led Cyrene to be considered as a solvent with potentially tunable or switchable properties.^126^ The use of Cyrene with bases however must be appropriately considered as a number of inorganic bases react with Cyrene, catalyzing aldol addition. Furthermore, amines can react with Cyrene if heated, although triethylamine at 30 °C has been found to be tolerated.^127^

### 2.2 Cygnet Solvents

**Figure 7:**
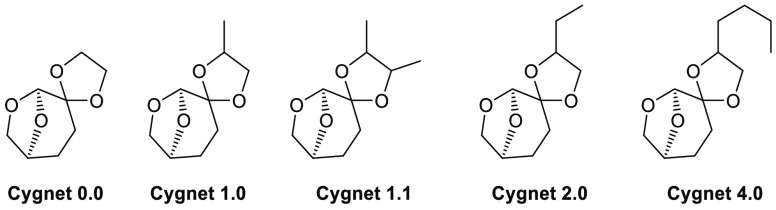
Cyrene derived “Cygnet” family of solvents.

The Cygnet class of solvents, Figure 7, refers to functionalized, ketal-protected variants of the previously discussed Cyrene.^66^ These derivatives are more stable than the unprotected ketone Cyrene, and were purposely designed to act as more inert forms of the original solvent, given that the ketone can be unstable under acidic conditions. The simplest Cygnet variant, Cygnet 0.0, is functionalized such that the ketone is protected with ethylene glycol, with the higher variants protected with derivatized ethylene glycol variants.^128^

Each variation can be prepared by condensation of the corresponding diol with Cyrene in the presence of an acid clay catalyst, liberating only water as a byproduct.^66^ Five variants of Cygnet have been synthesized to date, with their nomenclature based on the number of carbon atoms in the chain attached to each of the two ketal carbon atoms (e.g., Cygnet 1.1 contains 1 carbon atom, a methyl group, attached to each carbon of ethylene glycol). Because of the increased molecular weight of Cygnet derivatives compared with Cyrene, the melting point of each solvent (e.g., for Cygnet 0.0, > 70 °C) can lead to more difficult handling as a solvent. Of particular interest is the use of Cygnet 0.0 as a solvent, given that the predicted Hansen Solubility Parameters (HSP) place it in a similar part of Hansen Solubility Space as dichloromethane (DCM); however, alternatives to chlorinated solvents were the focus of a recent review by some of the authors of this work and are not covered here.^8,66^ To date, Cygnet 0.0 has been screened as a potential green solvent for dissolution of polymers for NIPS membrane casting.^128^ Toxicity and mutagenicity testing is underway for Cygnet 0.0, although Cygnet variants are predicted to be nontoxic and nonmutagenic.^66^ Cygnet 0.0 and Cyrene blends have been successfully utilized in the biocatalytic synthesis of polyesters, effectively replacing traditionally employed polar aprotic solvents.^129^

### 2.3 Carbonates and Cyclic Carbonates

**Figure 8:**
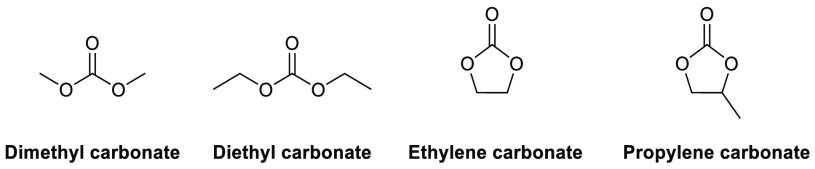
Some commonly employed carbonate-based solvents.

Carbonate-based solvents count dimethyl carbonate (DMC), diethyl carbonate, ethylene carbonate, and propylene carbonate, see Figure 8, among the most popular and widely used exemplars. Of the aforementioned solvents, propylene and ethylene carbonate score most favorably according to the GSK solvent selection guide,^44^ though their elevated boiling points (242 and 248 °C, respectively) may preclude their use in some applications. Depending on the polarity of the product, partitioning of high boiling solvents into an aqueous layer followed by product extraction with an immiscible nonpolar solvent can be achieved.^2^ However, this additional step inevitably increases process mass intensity and waste generation via the introduction of an additional solvent. Propylene carbonate synthesis and production have been discussed by Forero et al. in their review of propylene carbonate as a green solvent;^130^ multiple methods utilize CO_2_ as a carbon source in conjunction with other potentially renewable starting materials. Propylene carbonate occupies a Hansen solvent space close to acetonitrile, see Table 2, giving a rough indication of comparable solvating ability. Potential applications for carbonate based solvents include, but are not limited to, metal catalyzed cross-coupling reactions,^107^ proline-catalyzed aldol reactions,^131^ hydrosilylation,^132^ cyanohydrin synthesis,^133^ proline-catalyzed hydrazination,^134^ amine-electrophile S_N_2 chemistry,^135^ and the Appel reaction.^136^

### 2.4 γ-Valerolactone (GVL)

**Figure 9:**
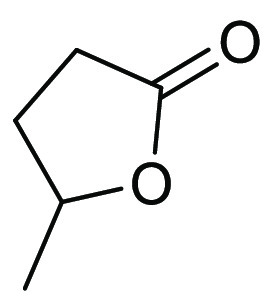
γ-Valerolactone.

γ-Valerolactone, see Figure 9, is a high boiling (207 °C) dipolar aprotic solvent and platform molecule^137^ that can be isolated from lignocellulosic biomass via levulinic acid.^138^ Multiple reviews and methods of its production have been published; 128 patents mention GVL, and it is present as a product in 567 separate publications.^139^ GVL has enjoyed moderate uptake as a reaction solvent across a wide variety of chemistry types. A search conducted using SciFinder shows that GVL has been employed as reaction solvent in 750 reactions across 108 publications, more than three-quarters of which have been published since 2014.^140^ Applications of this solvent as a reaction medium including palladium-catalyzed cross-coupling,^141^ electrochemistry,^142^ lignin depolymerization,^143^ C–H activation chemistry,^144^ and solid phase peptide synthesis.^145^ The success and wide variety of applications in which GVL has been employed are in part due to GVL’s favorable solvent properties; Kamlet–Taft parameters are comparable to DMF and DMAc and COSMO-RS modeling, and HSP comparisons have shown GVL to be most similar to acetone, NMP, DMF, and DMAc.^146^ Biodegradation studies have shown GVL to rapidly and completely break down in aquatic environments, and it has been demonstrated to be less toxic to humans that comparable dipolar aprotic solvents.^146^ Caution however must be taken when utilizing GVL as it is a known prodrug for γ-hydroxyvaleric acid (GHV), which itself has been noted as an alternative for UK Class C drug γ-hydroxybutyric acid (GHB),^147^ possession of which is punishable by up to 2 years imprisonment. Therefore, it is the authors suggestion that use and inventory/storage of this solvent be treated with caution due to its sedative properties, potential for abuse, and potential for future legislative restriction. Furthermore, due to the aforementioned concerns, the authors have opted not to recommend GVL as a replacement solvent in the condensed solvent selection guide provided in the Supporting Information (Unified Solvent Selection Guide for Dipolar Aprotic Solvents).

### 2.5 Sulfolane

**Figure 10:**
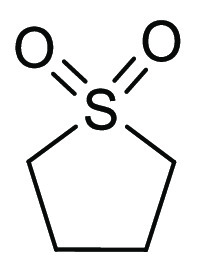
Sulfolane.

Sulfolane, see Figure 10, is a high-boiling (285 °C) dipolar aprotic solvent developed by Shell, and it has seen use in industrial applications in gas and oil refining, for example, the Sulfinol Process.^148^ Sulfolane can be produced industrially via the reaction of SO_2_ with butadiene followed by hydrogenation of the intermediate sulfolene to give sulfolane.^149^ HSP values for sulfolane suggest that is has very high solubilizing power for polar compounds, similar to DMSO, see Table 2. Sulfolane also possesses one of the highest Kamlet–Taft polarizability/dipolarity scores (π* = 0.90) of any of the solvents in Table 2, only DMSO is higher at 1.00. Sulfolane’s hydrogen bond basicity β is much lower however at 0.39 compared to DMSO 0.76 and is closer to propylene carbonate in this parameter (π* = 0.90, β = 0.38). Sulfolane is immiscible with alkanes and MTBE, which can allow for facile workups and product extraction of MTBE soluble products.^149^ Sulfolane has previously been considered as a safer dipolar aprotic solvent including for the fact that sulfolane has a much lower skin permeability than DMSO, DMF, DMAc, and NMP.^149^ Sulfolane, however, has been identified as potentially more hazardous than originally thought. A study on the reproductive toxicity effects of sulfolane in rats has suggested that the solvent could harm unborn child.^150^ Some SDS now carry a H360 warning to reflect this evidence.^151^ Furthermore, sulfolane has been recategorized according to the Chem21 solvent selection guide from “recommended” to “hazardous”.^43^ Sulfolane contamination of groundwater and drinking water was reported near the oil refinery in the community of Fairbanks, Alaska.^152^ Thus, it is the authors’ recommendation that sulfolane is not considered as a suitable green solvent. For a review of reactions conducted using sulfolane, see Tilstam et al.^149^

### 2.6 2-Methyltetrahydrofuran (2-MeTHF)

**Figure 11:**
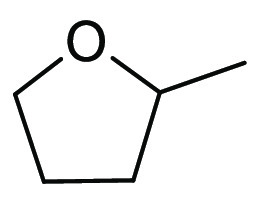
2-Methyltetrahydrofuran.

2-Methyltetrahydrofuran, see Figure 11, is an ethereal solvent that can be derived from renewable resources such as levulinic acid or furfural,^153^ which can themselves be derived from agricultural waste such as corn stover, sugar cane bagasse, and rice straw.^154^ Life cycle assessment conducted on 2-MeTHF has shown that production of the solvent from furfural derived from corn-cob waste can reduce emissions by up to 97% when compared to a traditional chemical synthesis of THF,^155^ though it is not always clear how individual supplies of 2-MeTHF are currently made. Structurally, 2-MeTHF differs from THF by the addition of one single methyl group, yet this addition is responsible for several significantly different physiochemical properties. First, the boiling point is elevated by 13 °C, see Table 1. 2-MeTHF is significantly less miscible with water at room temperature (129.2 g/L)^156^ than THF (494.7 g/L).^157^ This immiscibility has led 2-MeTHF to be employed as an extraction solvent and also as an organic component in aqueous–organic biphasic reaction mixtures.^158^ Interestingly, 2-MeTHF is also inversely soluble in water; at 0 °C, 2-MeTHF is 21 wt % soluble in water, whereas at 19 °C, this is reduced to 14 wt % and at 50 °C it is just 7.8 wt %.^159^ 2-MeTHF is also more stable to acidic hydrolysis than THF and has a longer *t*_1/2_ for R-Li mediated decomposition when compared to THF (130 min vs 10 min at 35 °C),^158^ though it is still susceptible to peroxide formation.^160^ As 2-MeTHF occupies a very similar solvent space to THF, see Table 2, it has been promoted as a “drop-in” replacement for THF that is greener and more sustainable. A number of reagent solutions are now commercially available in 2-MeTHF such as Grignard and lithium reagents,^161^ further promoting their uptake as viable alternatives to THF and diethyl ether.^153^ Use of 2-MeTHF has seen gradual increase as noted in the solvent use survey conducted by Ashcroft et al. (14% of publications in OPR&D for the period of 2009–2012 utilized 2-MeTHF in some stage of the reported synthesis).^9^ Many reviews of solvents ignore price, as such information can vary greatly depending on geography, purchasing contracts, or time. However, it is instructive to note that a price comparison of 2-MeTHF and THF was conducted in 2021 using Sigma-Aldrich’s website, see Table 3. Search criteria used were anhydrous solvents containing 250 ppm butylated hydroxyl toluene (BHT) inhibitor and a purity of ≥99.9%. Cost per liter of 2-MeTHF is nearly 2.5-times greater than standard THF at the purity and grade that were considered. However, economies of scale come into effect at 200 L drum size; 2-MeTHF was only 13% more expensive than THF. Depending on the details of the reaction concerned, it is therefore easily conceivable that the increased cost of purchase could be offset by other reduced costs (e.g., less aqueous waste for incineration). For further reviews of 2-MeTHF use, see Pace et al. 2012,^153^ Monticelli et al. 2017,^160^ and most recently Bijoy et al. 2021.^162^

**Table 3:** Cost Comparison for THF and 2-MeTHF According to Sigma-Aldrich^163,164^

solvent	cost (1 L)	cost (200 L)
THF	£ 55.20	£ 4492.00
2-MeTHF	£ 136.00	£ 5082.00

### 2.7 Cyclopentyl Methyl Ether (CPME)

**Figure 12:**
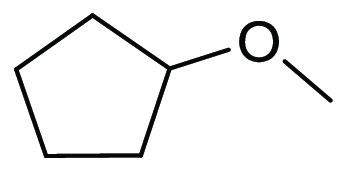
Cyclopentyl methyl ether.

**Scheme 2:**
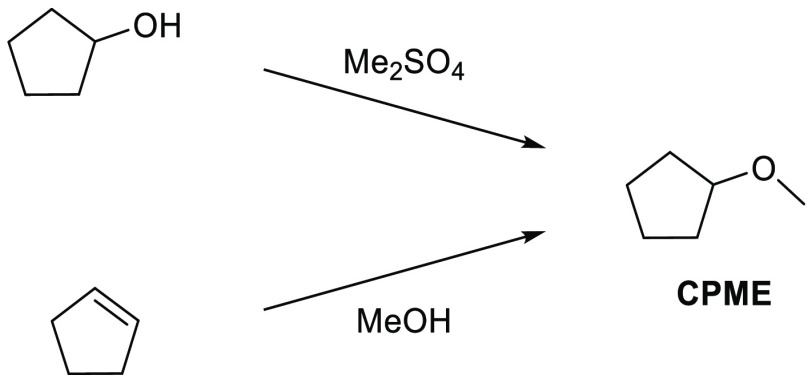
Synthetic Routes to CPME^165^

Cyclopentyl methyl ether, see Figure 12, is a hydrophobic ethereal solvent with a high boiling point (106 °C), see Table 1. It became a credible alternative to many ethereal solvents, such as THF and 1,4-dioxane, when commercial quantities became available in 2005.^165^ CPME can be produced from either cyclopentanol or from cyclopentene, see Scheme 2.^165^ The similar HSP parameters of CPME to other ethereal solvents (and their proximity to one another in Hansen space), such as 1,4-dioxane and THF, give an indication that CPME likely possesses similar solubilizing capabilities,^80^ see Table 2. CPME can be made from renewable resources, although it is not currently commercially available via a biobased route, and offers other benefits, including significantly safer handling and relative stability under acidic and basic conditions.^165^ Peroxide formation is still an issue however, and butylated hydroxytoluene (BHT) is still required as an additive to prevent oxidation.^165,103,166^ Toxicological assessment of CPME was conducted by Watanabe et al. in 2013, and it was shown to have relatively low acute toxicity, and was shown not to be a skin sensitizer nor a mutagen. Eye and skin irritation were however observed.^167^ The potential for this solvent to become a solvent for use in process chemistry was reviewed in 2007^165^ and more recently in 2019^168^ where its position as a more sustainable, environmentally friendly ether has become reinforced. Applications where it has been successfully employed include organometallic chemistry, transition metal catalysis, acid catalysis, amidation reactions, oxidations, radical reactions, and as an organic phase in biphasic chemistry.^168^ For specific examples, see reviews by Azzena et al.,^168^ Watanabe et al.,^165^ and most recently Bijoy et al.^162^

### 2.8 Methyl *tert*-Butyl Ether (MTBE)

**Figure 13:**
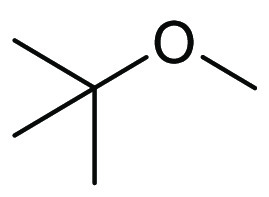
Methyl *tert*-butyl ether.

Methyl *tert*-butyl ether (MTBE), see Figure 13, sometimes referred to as *tert*-butyl methyl ether (TBME), is a volatile (bp 55 °C), flammable, colorless liquid that is commonly used as a fuel additive to reduce detonation risk in addition to reducing unwanted emissions.^169^ Similar to other ethers, MTBE is often used in the place of THF and diethyl ether due to its significantly lower risk of peroxide formation, with the *tert*-butyl group thought to contribute to this.^170^ In comparison with other ethereal solvents, MTBE does, however, have a much lower flash point (−28 °C, compared with −14.5 °C for THF and −1 °C for CPME), see Table 4.^166^ Furthermore, there have been some health concerns related to MTBE, with links to endocrine disruption.^172^ MTBE is synthesized industrially in a similar way to CPME, through the reaction of methanol (derived from natural gas), and isobutylene, sourced from the dehydrogenation of isobutane.^173^

**Table 4:** Additional Physicochemical Properties and Peroxide Forming Abilities of a Selection of Ethers

	CPME	2-MeTHF	THF	MTBE
Solubility in water (23 °C) (g/100 g)	1.1	14	miscible	4.8
Flash point (°C)	–1	–11	–14.5	–28
Peroxide formation	slower	faster	faster	slower
Acid stability	more stable	less stable	unstable	unstable
Boiling point (°C)	106	80	66	55
Dielectric constant (25 °C)	4.76	7.0	7.4	2.6
Dipole moment (D)	1.27	-	1.7	1.4

MTBE has been used as a solvent in reaction classes including C–H borylations,^174^ Suzuki-Miyaura couplings,^174^ palladium-catalyzed silyl enolate alkylations, and Sonogashira cross-couplings.^175^ It has additionally been known to be used as an alternative greener solvent in normal-phase column chromatography,^176^ and as an alternative extraction solvent due to its immiscibility with water.^177^ Unlike other ethers, the low Lewis basicity of MTBE results in incompatibility with formation of Grignard reagents and limits use in lithiations.

### 2.9 Cyclic Ketones: Cyclohexanone and Cyclopentanone

**Figure 14:**
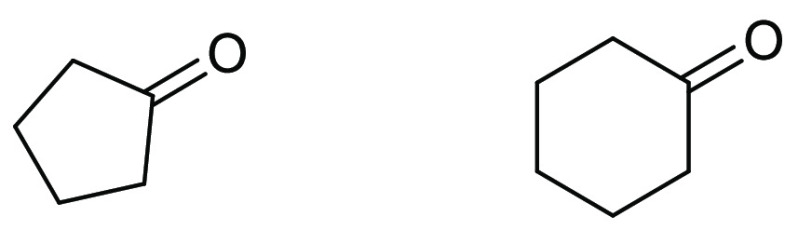
Cyclic ketones cyclopentanone (left) and cyclohexanone (right).

Both cyclopentanone and cyclohexanone, see Figure 14, have been promoted as alternative dipolar aprotic ketone solvent replacements that can be potentially produced from biorenewable resources.^178^ Cyclopentanone can be produced from the biobased platform molecule furfural,^179^ and cyclohexanone by catalytic reduction of aromatic ethers such as anisole.^180^ Both solvents score highly according to the GSK solvent selection guide as potentially greener and more sustainable ketone solvents for myriad reasons including favorable recyclability scoring, minimal known measured aquatic impact, moderate to good exposure and health hazard scoring, and a good reactivity and flammability score.^44^ Both cyclopentanone and cyclohexanone have boiling points that are elevated (cyclopentanone 129 °C, cyclohexanone 155 °C (Table 1) and occupy a similar Hansen solubility space to each other when comparing δD and δH. However, the polarity parameter or δP differs significantly; cyclopentanone is 11.9, whereas cyclohexanone is 8.4, see Table 2, suggesting that cyclopentanone is more polar than cyclohexanone. This is at odds with the Reichardt polarity parameter (0.281 vs 0.269, Table 1). When examining the Kamlet–Taft parameters, cyclopentanone possesses a larger π* (0.76 vs 0.68) and both solvents have nearly identical hydrogen bond basicity β of (0.52 and 0.53). π* as a measure of dipolarity/polarizability is in agreement with the information provided by the HSP parameters; cyclopentanone is “more polar” by this measure than cyclohexanone. Cyclopentanone has been successfully employed as solvent in several processes. A SciFinder analysis of the literature shows that 74 nonparticipating single-step transformations have been reported in 29 different journal publications.^181^ Uses include solvent in photoredox catalysis,^182^ aldol reactions (as both solvent and reagent),^183^ and solvent for Steglich-type thioester formation,^184^ transition metal catalysis,^185^ and total synthesis.^186^ Similarly, cyclohexanone has seen use in 1545 reactions in 421 journal publications.^187^ Uses include transition metal catalysis,^188,189^ aldol reactions,^190^ total synthesis,^191^ and as a general synthesis solvent in medicinal chemistry drug discovery.^192^ Aside from their use as solvents, both cyclic ketones have found significant use as organic chemistry building-blocks; cyclohexanone is an important precursor for Nylon-6.^193^

### 2.10 2,2,5,5-Tetramethyloxolane (TMO)

**Figure 15:**
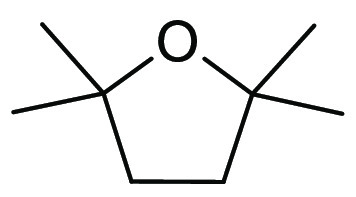
2,2,5,5-Tetramethyloxolane.

TMO or 2,2,5,5-tetramethyltetrahydrofuran (TMTHF), see Figure 15, is a volatile nonpolar (VNP) ether that was identified as a potential replacement for volatile and peroxide forming ethers and also toluene as part of the ReSolve project.^194^ Specific properties of a boiling point of <115 °C and nonperoxide forming characteristics were required, and TMO fulfilled these criteria (bp 112 °C, Table 1) from a selection of other quaternary alpha-carbon ether hydrocarbons that were considered.^103^ TMO can be synthesized from 2,5-dimethylhexane-2,5-diol (which itself can be potentially produced from renewable resources) via a dehydration reaction.^103^ Additionally, a recent report by Byrne et al. compared a selection of biobased synthetic routes to TMO from glucose, one of which included use of methyl levulinate (a byproduct of polyethylene furanoate production, Avantium).^195^ Each potential route was further analyzed by a range of metric tools for comparison.

TMO’s Kamlet–Taft parameters were measured and the HSP parameters calculated using HSPiP software. The Kamlet–Taft properties showed that TMO had strong hydrogen bond accepting character (β = 0.77, Table 2) and a low polarity-polarizability character (π* = 0.35). As per the other aprotic solvents, the hydrogen bond donating ability α was zero. In practice, however, the tetramethyl groups adjacent to the ether oxygen in TMO introduce significant steric bulk and essentially shield the lone-pairs, thus reducing their ability to accept hydrogen bonds. The effects of the steric hindrance are consistent with the calculated HSP values, which show that TMO has a hydrogen bonding ability (δH) of 2.1; for comparison, THF has a δH of 8.0, see Table 2. The low δH value translates into real-world chemical reactivity effects. For example, Byrne et al. have demonstrated that TMO is a poor solvent for the formation of some Grignard reagents due to the poor accessibility of the oxygen lone-pairs (despite strong Lewis-acid characteristics). TMO was shown to behave more like hydrocarbon solvents such as toluene, giving excellent rates of reaction in esterification and amidation reactions.^103^ TMO has also been assessed for its potential as an extraction solvent in aqueous–organic extractions and was shown to be a particularly useful solvent in extracting hydrogen bond donating solutes from aqueous systems when toluene was not.^196^ Pellis et al. have investigated the use of TMO as an alternative solvent to toluene and THF in the biocatalyzed polymerization of polyesters,^197^ and its resin swelling ability has been assessed by Yanrui et al.^198^ In a more niche use, TMO also performs effectively as a solvent for the oxidation of (hydroxymethyl)furfural to diformylfuran.^199^

### 2.11 Eucalyptol

**Figure 16:**
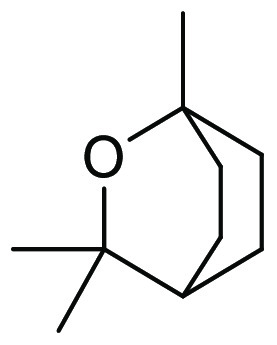
Bicyclic ether “eucalyptol”.

Eucalyptol is a naturally occurring bicyclic ether, see Figure 16, belonging to the monoterpenoid family, and is the major component of eucalyptus oil.^200^ The high-boiling liquid (176 °C) is insoluble in water and soluble in organic solvents including alcohols, ethers, and chloroform.^201^ Also known within the chemical literature as 1,8-cineole, the oil is manufactured globally from tree species within the *Eucalyptus* genus, with over 300 species known to contain the oil. These trees are commonly grown for the purpose of timber production, and the extraction of eucalyptol from the “waste leaf” from timber operations makes the source of the oil potentially sustainable.^202^ Pure eucalyptol is obtained from the “waste leaf” in a two-step process, distillation of harvested leaf with steam, followed by freezing of the crude material at −40 °C, with removal of unfrozen limonene impurities (usually 2–7%).^202^ Eucalyptol, like a number of other dipolar aprotics solvents, is hygroscopic. The oil is known to have medicinal applications including potent anti-inflammatory and antioxidant properties.^203^ Because of its use as a medicine, in addition to a common food additive, eucalyptol has been extensively tested and can be considered to have a low risk of toxicity.^204^ To date, very few publications (<10) have been reported using eucalyptol as a sustainable solvent for organic transformations, although it has been shown to be an effective solvent in applications such as transition-metal catalyzed C–C, C–O, C–N, and C–S bond formation^205−207^ in addition to carboxylations.^208^

### 2.12 Dimethyl Isosorbide (DMI)

**Figure 17:**
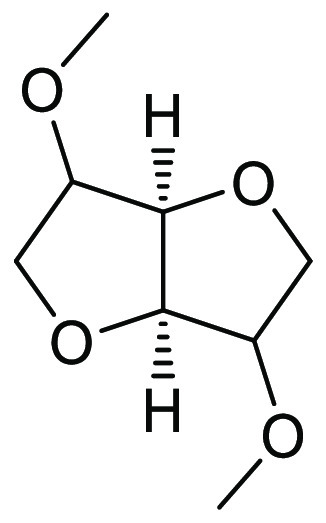
Dimethyl isosorbide: a sorbitol derived dipolar aprotic solvent.

Dimethyl isosorbide is a high boiling (235 °C) dipolar aprotic solvent, see Figure 17, that can be synthesized from biorenewable resources such as cellulose (in two steps via glucose and then sorbitol)^209^ or via direct methylation of platform molecule isosorbide.^210−213^ DMI has been assessed for its toxicity and mutagenicity and did not display acute oral toxicity nor genotoxicity by Ames test.^118^ DMI was shown to be not readily biodegradable.^118^ DMI’s Kamlet–Taft properties show that it possesses dipolarity/polarizability that is similar to DMF and DMAc; however, its hydrogen bond basicity is much less, and it is closer to acetone in that respect. Applications for DMI include general synthesis as a replacement for traditional dipolar aprotic solvents in palladium-catalyzed cross-coupling,^214^ solid phase synthesis,^215^ and membrane preparation.^216^ Uptake of DMI for use in the personal care products industry has also been noted.^217^ A search of current literature using SciFinder^218^ shows that the uptake of DMI as a reaction solvent is still quite low, with only 63 reported reactions in just four publications.^208,214,219,220^

### 2.13 *N*-Butyl-2-pyrrolidinone (NBP)

**Figure 18:**
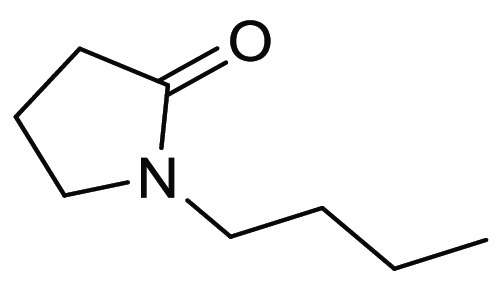
*N*-Butyl-2-pyrrolidone: a potential NMP drop-in replacement.

Following a false start exploring whether *N*-ethyl-2-pyrrolidinone could offer advantages over the reprotoxic *N*-methyl-2-pyrrolidinone (NEP was found to have reproductive toxicity concerns),^221^*N*-butyl-2-pyrrolidinone, see Figure 18, has been identified as a potential alternative to NMP due to its favorable characteristics: NBP is nonreproductively toxic (according to OECD 414 test method), nonmutagenic (OECD 471), and also inherently biodegradable (OECD 302B).^110^ It is also possible to produce NBP from biorenewables.^110^ The apparent lack of reproductive toxicity makes it an attractive alternative to NMP; however, NBP possesses acute oral toxicity that is greater than NMP so caution must still be exercised when using this solvent. NBP resides in a similar solvent space to other dipolar aprotic solvents such as NMP, DMF, and DMAc, see Table 2, and due to these similarities has been proposed as a somewhat safer “drop-in” alternative, though it should be noted the boiling point of NBP is significantly elevated at 241 °C, see Table 1, S_N_2 reaction kinetic experiments using the Menschutkin reaction as a model reaction have shown that the rate of reaction is highly dependent on the Kamlet–Taft parameter π* of the dipolar aprotic solvents screened; higher values lead to faster rates of reaction. NBP’s π* value of 0.77 is closest to acetonitrile’s of 0.75, and indeed the most comparable rate of reaction experimentally observed for NBP was acetonitrile, seeFigure 19. NBP has also been examined for use as a drop-in replacement for DMF in solid supported peptide synthesis.^222^

**Figure 19:**
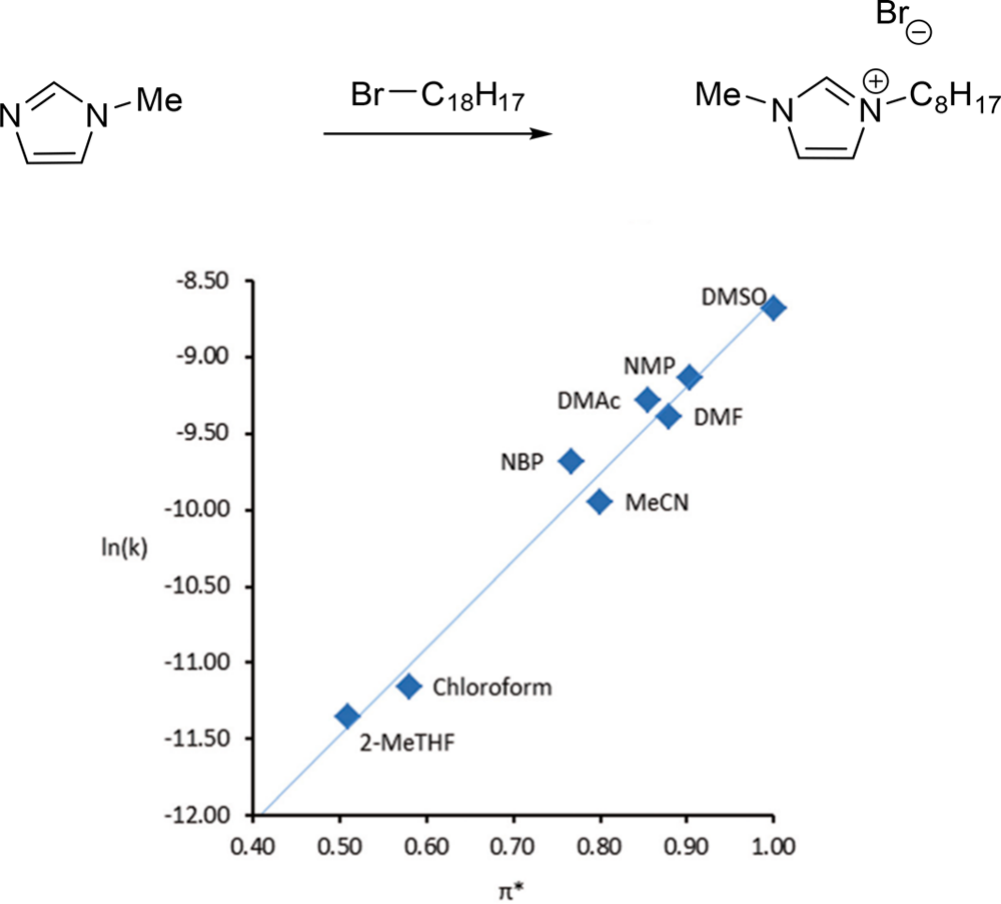
Linear solvent energy relationship, ln(*k*) versus π*, describing the rate of reaction of a Menschutkin reaction. Reproduced from ref (110) with permission from the Royal Society of Chemistry.

### 2.14 Methyl-5-(dimethylamino)-2-methyl-5-oxopentanoate (Polarclean)

**Figure 20:**
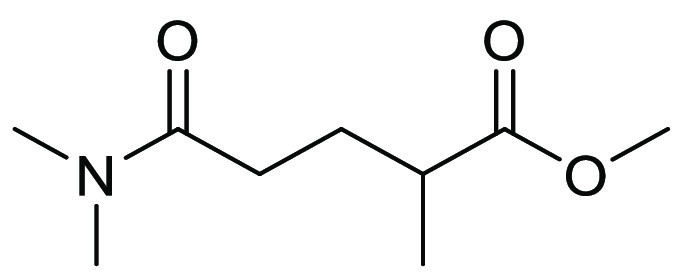
Methyl-5-(dimethylamino)-2-methyl-5-oxopentanoate: PolarClean.

**Scheme 3:**
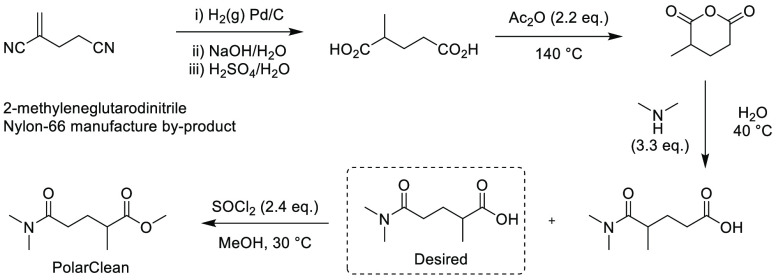
Synthetic Path to PolarClean Utilizing Nylon-66 Byproduct as Starting Material^225^

Methyl-5-(dimethylamino)-2-methyl-5-oxopentanoate, see Figure 20, otherwise known commercially as Rhodiasolv PolarClean, is a clear, slightly yellow solvent. The nonvolatile liquid has a boiling point in the range of 278–282 °C, a melting point of −60 °C, and a flash point of 144–146 °C. Similar to other dipolar aprotic solvents, PolarClean is highly water miscible, with a solubility of greater than 490 g/L.^223^ It is reported that the hygroscopicity of the solvent is very low, with water content values estimated between 0.00 and 0.10%, although some values up to 0.30% have also been reported.^223^ Prior to increased use within synthetic chemistry, use as a crystal growth inhibitor in agrochemical formulations has been reported.^224^ A contributing factor to the sustainability credentials of PolarClean arises from its production; it is produced on an industrial scale from a byproduct of Nylon-66 manufacture, which would otherwise be burnt for disposal.^224^ The production of PolarClean from this byproduct, methyleneglutarodinitrile (MDN), involves a multistep process. Starting from MDN, the route consists of hydrogenation, hydration, cyclization, and subsequent ring-opening with dimethylamine, followed by methylation of the remaining carboxylic acid, see Scheme 3, yielding a mixture of two regioisomers.^225^ Further work has been described to redesign and improve the synthetic route to PolarClean by Szekely and co-workers.^104^

Similar in structure to PolarClean, a small number of diamide dipolar aprotic solvents derived from succinic acid were reported by Byrne and co-workers in 2020.^226^ Designed to have high dipolarity and low toxicity, the solvents were shown to perform effectively in the Heck cross-coupling reaction of iodobenzene and methyl acrylate in addition to potential uses in polymer solubilization and MOF synthesis.^226^

### 2.15 Surfactant-Based Amphiphiles

A novel class of surfactant-based amphiphiles, including PS-750-M (also known as FI-750-M), polyoxyethanyl-α-tocopheryl sebacate (PTS), d,l-α-tocopherol methoxypolyethylene glycol succinate (TPGS-750-M), and SPGS-550-M (Nok), has recently emerged, with the aim of facilitating efficient organic chemical reactions in water.^227^ When dissolved in water, the surfactant amphiphilic molecules self-aggregate into micelles, with the hydrophilic “head” interacting with the aqueous medium, while the hydrophobic “tails” form an inner “lipophilic core”.^227^ The nanometer-sized particles formed during this aggregation process can be thought of as nanoreactors, with localized pockets of high concentration reaction substrates, leading to rapid reaction rates.^228^ Use of these surfactants in an aqueous media can allow significant reduction in the use of commonly used dipolar aprotic solvents in reactions such as transition metal-catalyzed cross-couplings,^229^ leading to a reduction of organic solvent waste streams. When suitably optimized, these systems have been demonstrated, often on large scale,^230^ to lead to significant yield and selectivity improvements, reduced catalyst loadings,^231^ lower environmental footprints, and a general improvement in productivity.^232^ Challenges associated with the use of aqueous-surfactant systems include a lack of total generality (often a surfactant can be demonstrably compatible with one class of reactions/starting materials and incompatible with another), only an empirical/trial and error based understanding of reactions and why they work, mixing effects can be pronounced,^233^ preferential solubilization/dispersion of reagents versus products can affect reaction outcome,^234^ lack of in-house precedent/expertise to encourage the use of less mainstream reaction media, and extraction and isolation of products by conventional organic solvents. “Synthetic chemistry rules most people learn are not universal. They apply to reactions in organic chemistry, but chemistry in water obeys different rules”;^235^ thus, discovering and learning these rules is an ongoing goal in the field. Many of the surfactants described in this section are commercially available at the time of writing, whereas others are “designer”, that is, tailored specifically for specific transformations. The aim of this section is to introduce the reader to some of the cutting edge research in the field of organic synthesis in aqueous-micellar systems. It also serves to demonstrate that water does not have to be the enemy of the organic chemist but a powerful ally.^236^ For further details, an excellent review discussing the challenges and opportunities faced by aqueous-micellar organic synthesis was published in 2021 in collaboration with Novartis (Gallou et al.).^237^

#### 2.15.1 PS-750-M

**Scheme 4:**
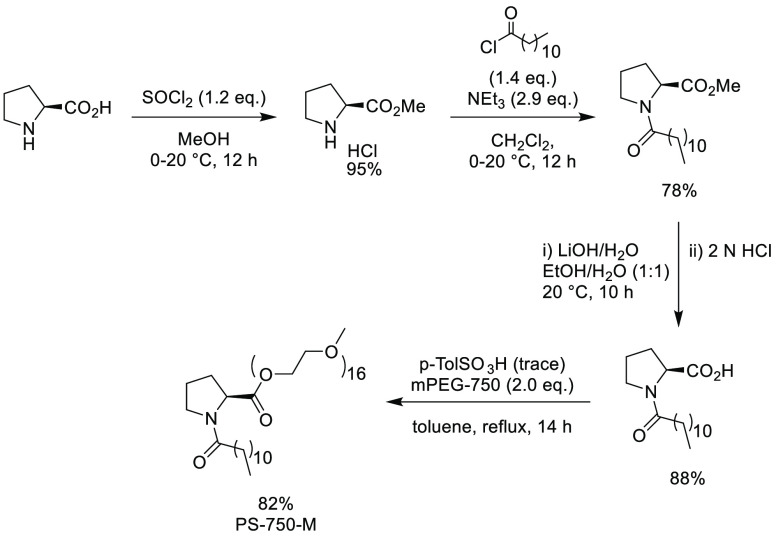
Synthetic Pathway to PS-750-M^238^

PS-750-M, also known as FI-750-M, is a surfactant first described in 2017 in a publication by Handa et al. in collaboration with Novartis, for an aqueous media-based palladium-catalyzed coupling of nitroalkanes and aryl bromides.^238^ Derived from the naturally occurring amino acid l-proline, the surfactant can be prepared in a high-yielding 4-step synthesis (overall 53% across 4 steps, Scheme 4). Since the initial publication, the surfactant has been used to enable aqueous reactions including S_N_Ar,^239^ α-arylations,^240^ fluorination of indoles,^241^ and Pd carbene-mediated insertions.^242^

#### 2.15.2 Polyoxyethanyl-α-tocopheryl Sebacate (PTS)

**Scheme 5:**
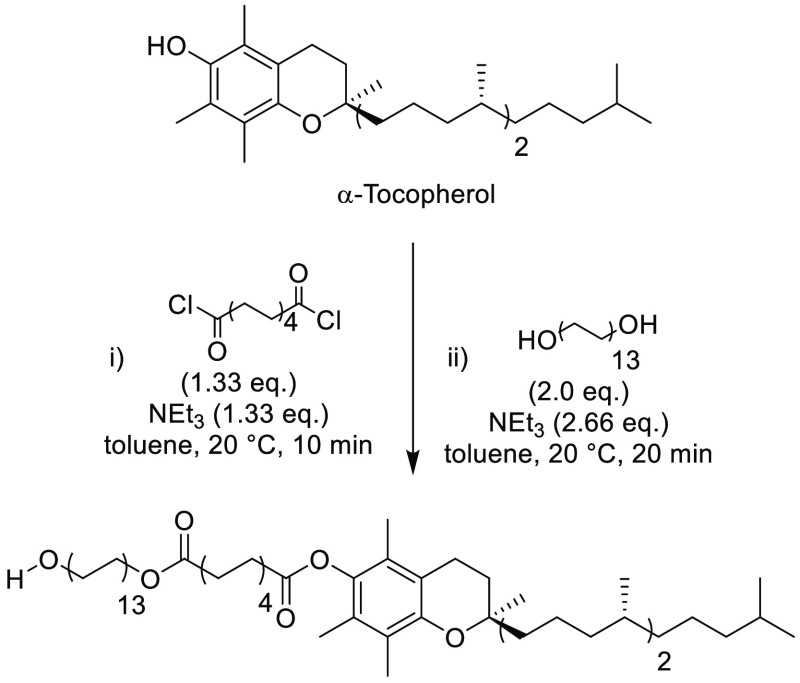
Preparation of Polyoxyethanyl-α-tocopheryl Sebacate (PTS) from α-Tocopherol

Polyoxyethanyl-α-tocopheryl sebacate (PTS), see Scheme 5, is a first generation surfactant to be shown to have utility in organic synthesis by Lipshutz and co-workers in 2008. Derived from the naturally occurring vitamin E307, α-tocopherol, PTS can be prepared by first condensing with sebacoyl chloride, followed by dropwise addition of PEG-600, both steps being carried out in the presence of triethylamine.^243^ Since first being described in organic synthesis as a surfactant, publications include Suzuki-Miyaura,^244,245^ Heck,^246^ Sonogashira,^247^ and Buchwald-Hartwig couplings,^248^ in addition to olefin metathesis.^249,250^

#### 2.15.3 d,l-α-Tocopherol Methoxypolyethylene Glycol Succinate (TPGS-750-M)

**Figure 21:**
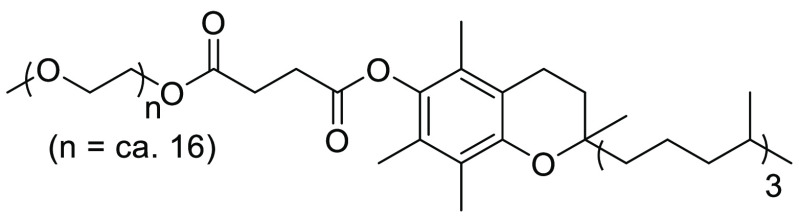
d,l-α-Tocopherol methoxypolyethylene glycol succinate also known as TPGS-750-M.

d,l-α-Tocopherol methoxypolyethylene glycol succinate (TPGS-750-M), see Figure 21, is the second-generation variant of the amphiphile PTS, reported by Lipshutz and co-workers in 2011.^55^ Closely related to polyoxyethanyl-α-tocopheryl sebacate (PTS), TPGS-750-M is again derived from the natural product α-tocopherol, differing in the carbon diacid and PEG chain link. Where PTS used the 8 carbon acid linker sebacic acid, and PEG-600, TPGS has a shorter 2-carbon diacid linker, in addition to the longer PEG-750-M chain. Preparation involves a similar sequence to that described for TPGS-750-M, with sequential condensation of α-tocopherol with succinic anhydride in the presence of triethylamine, followed by PEG-750-M.^55^ In the publications disclosing the surfactant amphiphile, its utility is shown in olefin metathesis, Suzuki-Miyaura, Heck, Sonogashira, Buchwald-Hartwig, Negishi, palladium catalyzed C–H activation, and palladium catalyzed allylations.^55^ A shortened, more stable version of this surfactant known as TPG-Lite has also been developed in which the four carbon succinic acid linker is omitted. Reaction compatibility is broadly that of TPGS-750M.^251^

#### 2.15.4 SPGS-550-M (Nok)

**Figure 22:**
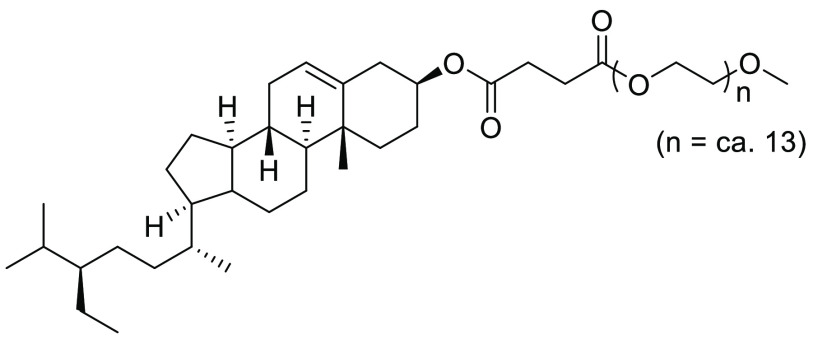
β-Sitosterol methoxypolyethylene glycol succinate SPGS-550-M (Nok).

In 2014, Lipshutz et al. described a further enhancement to the previously designed amphiphile surfactants, with a switch of the α-tocopherol to β-sitosterol, see Figure 22. The resultant amphiphile, SPGS-550-M, otherwise known as “Nok”, was designed to be a more economically attractive surfactant when compared with PTS and TPGS-750-M.^229^ Synthesis is similar to previous variants, with condensation of β-sitosterol with succinic anhydride, followed by further reaction with PEG-550-M.^229^ In the publication, through an extensive comparison process with TPGS-750-M, Nok was shown to perform as effectively as or more effectively than TPGS-750-M in a range of reactions including metathesis, Suzuki-Miyaura and Sonogashira cross-couplings, Miyaura borylations, and Buchwald Hartwig aminations.^229^ Recent developments by the Lipshutz group have seen the invention of a low foaming surfactant known as “Coolade”.^252^ It is known that foaming can occur in surfactants that possess long hydrophobic tails, the long tail leading to increased foam volume and stability.^252^ Coolade does not possess this tail, yet can still self-assemble into micelles. Typical reactions that suffered from foaming/frothing when conducted TPGS-750M, such as NaBH_4_ nitro reductions, were shown to be free from foam when employing Coolade. Foam is generally seen as an inconvenience at best with larger reaction volumes and vessels required.^252^ Workup and product isolation may also become complicated. Other reactions successfully demonstrated included double reduction of geminal dibromocyclopropanes, azide and sulfonyl azide reductions, and Suzuki-Miyaura coupling.

**Figure 23:**
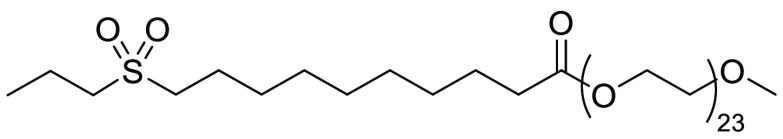
MC-1, a designer surfactant successfully used for peptide coupling under aqueous-micellar conditions.

In 2019, the DMSO inspired surfactant “MC-1” was developed by Cortes-Clerget et al., and it is a designer surfactant that specifically tailors to the needs of peptide chemistry, see Figure 23. The micelle accommodates both amino acids/peptide starting materials and typical coupling reagents such as COMU in a polar yet still lipophilic environment.^253^ Multigram synthesis of dipeptides was successfully conducted in high yields (>90%) using this surfactant.

**Figure 24:**
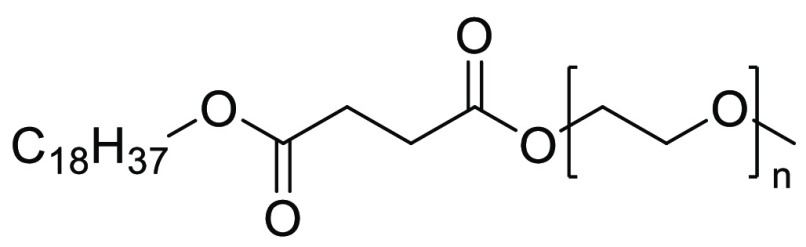
Designer surfactant SMPS used for Zn mediated nitro-arene reduction under aqueous-micellar conditions.^254^

Nonionic designer surfactant stearyl methoxyPEGglycol succinate (SMPS) was designed and successfully implemented in nitro-arene reduction and tethered indole synthesis by Kothandapani et al., see Figure 24(254) Apart from high yielding synthesis of the target substrates in this SMPS-water system, a preliminary toxicity screen was also conducted, which included phytotoxicity, bacterial toxicity, and aquatic toxicity. It was shown at the concentrations screened that seed growth was not affected compared to controls nor were there significant bacterial toxicity effects or harmful effects toward zebrafish during the aquatic toxicity screening.^254^ The surfactant design concept used here was structured around the “Benign by Design” principle central to green chemistry.^255^

**Figure 25:**
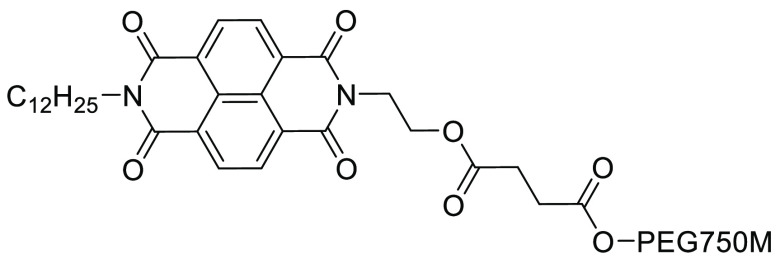
Designer surfactant PiNAP-750M exhibiting additional aromatic conjugation in the hydrophobic region.^256^

**Scheme 6:**
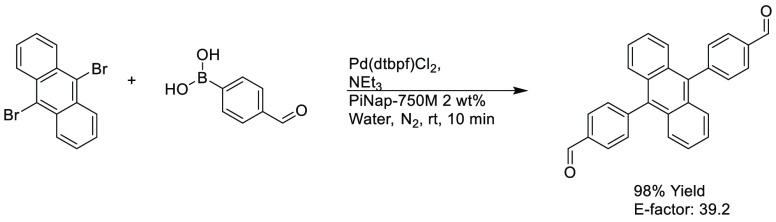
Example Organic Semiconductor Synthesis Achieved Using PiNAP-750M^256^

Utilizing surfactants in organic chemistry can be challenging and often lack generality when it comes to surfactant choice, that is, there is no one universal surfactant. Often, starting material solubility is problematic, and organic cosolvents have been utilized to change the nature and dynamics of the emulsions formed. One particularly problem was assessed by Sanzone et al. for the synthesis of rigid and poorly soluble organic semiconductor molecules.^256^ It was hypothesized that by increasing the aromatic content of the hydrophobic portion of the surfactant, additional π–π interactions could be facilitated between the surfactant and reaction starting materials. Thus, successful dispersion of starting materials that exhibit self-aggregation due to π–π interactions with themselves could be achieved. The surfactant designed, PiNAP-750M, see Figure 25, allowed for the generation of Pd-catalyzed cross-coupling products at room temperature, without cosolvent, and in short reaction times (of 10 min), see Scheme 6. Reaction times were much slower when conducted in commercially available surfactants such as K-EL (hours to days longer). E-factors were also demonstrated to be up to an order of magnitude lower than the currently published synthetic routes utilizing organic solvents when using the PiNAP-750M system.^256^

**Figure 26:**
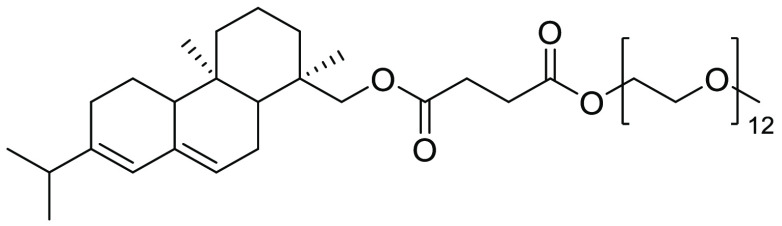
APGS-550-M, prepared from potential biorenewable sources such as rosin and succinic acid.

**Scheme 7:**
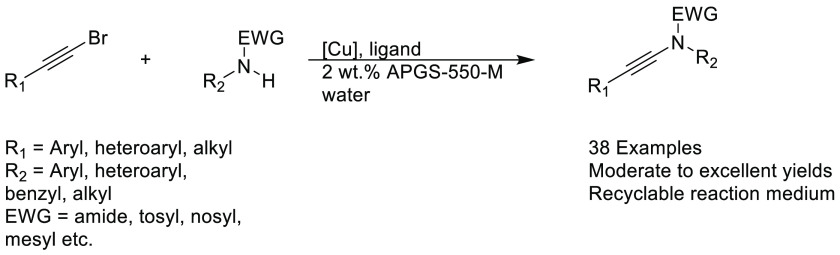
Cu-Mediated Ynamide Synthesis under Aqueous-Micellar Conditions^257^

Designer surfactants can also be prepared from biorenewable materials, such as the synthesis of APGS-550-M from resin acids (from rosin) such as abietic acid, see Figure 26. The surfactant, prepared by Yang et al., was shown to be highly effective in facilitating Cu-mediated amidation of alkynyl bromides under aqueous-micellar conditions, see Scheme 7.^257^ Normally, this type of transformation is conducted in dry solvents such as DMF, DMSO, or toluene at elevated temperatures. Side-reactions that can occur include homocoupling of alkynes to give 1,3-diynes, hydration of the diynes to furans, and hydration of ynamides to amides;^257^ thus, the presence of water in the system is somewhat counterintuitive. However, the micellar conditions allow for smooth conversion of bromoalkynes to the corresponding ynamides. A variety of alkyl and aryl groups were tolerated under the conditions and electron withdrawing groups (EWGs) such as amides, p-toluenesulfonamides, *N*-phenyl methanesulfonamides, oxazolidinones, and *N*-phenyl methanesulfonamides were all compatible with this methodology showing broad functional group tolerance for this type of transformation. Finally, the reaction medium was shown to be recyclable as long as additional copper was added, and by the third run using recycled medium, the reaction E-factor was as low as 5.

**Figure 27:**
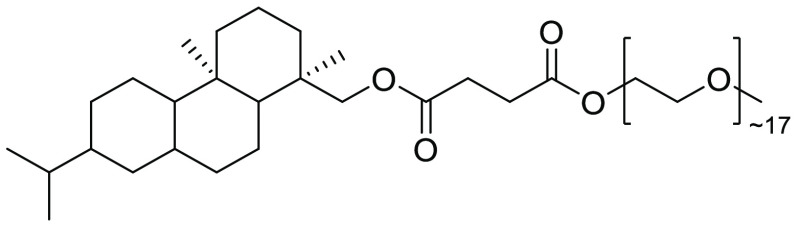
DAPGS-750M, a dehydroabietinol-derived surfactant.

**Scheme 8:**
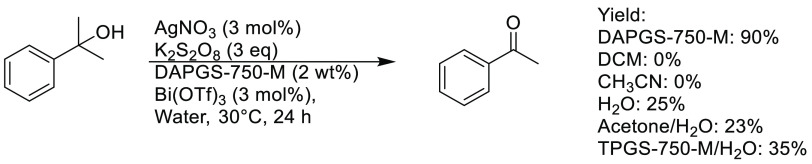
β-Scission Tertiary Alcohol Oxidation Conducted in an Aqueous Micellar System^258^

Chen et al. have also sought to exploit rosin as a potential source of raw materials for the synthesis of surfactants, see Figure 27.^258^ DAPGS-750-M is structurally related to APGS-550-M in that it is also derived from abietic acid. Aqueous-micellar conditions using this surfactant have allowed for β-scission of −CH_3_ from a range of aromatic tertiary alcohols to give corresponding ketones. The same reaction conducted in traditional organic solvents, binary aqueous–organic mixtures, and aqueous-micellar conditions such as TPGS-750-M, gave poor yields, see Scheme 8. One potential reason for this is due to the different shape, size, and distribution of the DAPGS micelles when compared to other surfactants examined in this publication.^258^ A variety of acetophenone-like molecules were prepared from the corresponding tertiary alcohols in moderate to excellent yields, though anilines were not tolerated at all. Furthermore, bulky β-carbon groups were preferentially eliminated in preference of methyl groups. In-flask recycling of the aqueous-micellar system was also demonstrated with an E-factor of 11 observed after three runs.

## 3 Common Reactions in Dipolar Aprotic or Ethereal Solvents Replaced by More Sustainable Alternatives

In this section, some commonly employed reactions using more sustainable, greener dipolar aprotic or ether based solvents will be discussed. Note: solvents in reactions schemes and tables are color coded according to the same traffic light system used in Table 1. It is envisaged that these case studies and examples will provide reliable literature references from which chemists can rely for inspiration and encouragement when potentially conducting solvent screening/swapping investigations of their own.

### 3.1 Amide Bond Formation

**Scheme 9:**
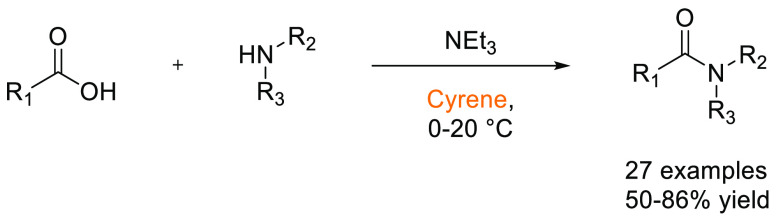
Formation of Amides from Acyl Chlorides and Amines in Cyrene^125^

**Scheme 10:**
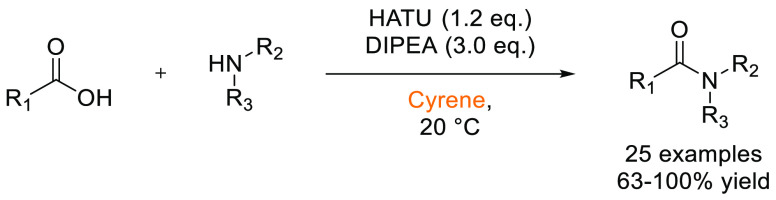
HATU-Mediated Synthesis of Amides from Carboxylic Acids and Amines in Cyrene^119^

The amide moiety is abundant within drug molecules,^259^ and amide coupling has been consistently highlighted as the most frequently employed transformation in medicinal chemistry (Scheme 9, Scheme 10).^260^ Traditionally, amide coupling has relied heavily on solvents with major regulatory issues such as dichloromethane and DMF, which are classed as solvents of concern.^44^ On the basis of the importance of amide bond formation, it is pertinent that greener and more sustainable solvent use should be explored whenever possible.

**Scheme 11:**
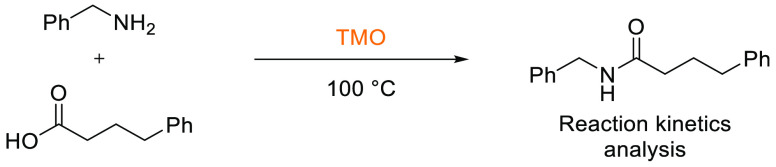
Catalyst-Free Amidation in 2,2,5,5-Tetramethyloxolane^103^

**Figure 28:**
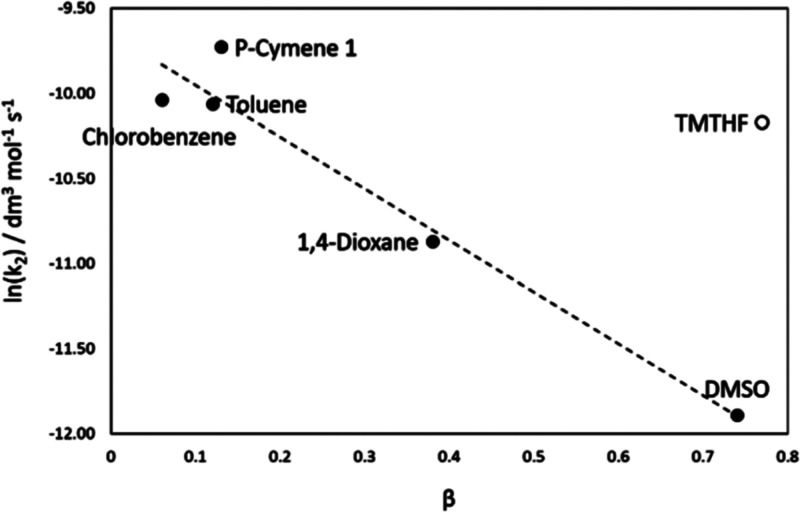
Reaction kinetic analysis for TMO (TMTHF)-mediated amide bond formation conducted by Byrne et al.^103^ Reproduced from ref (103) with permission from the Royal Society of Chemistry.

Several examples of amide coupling reactions employing previously discussed sustainable alternatives to dipolar aprotics are discussed below. Recently, Camp and co-workers reported the synthesis of amides from acid chlorides and amines in the biobased solvent Cyrene.^125^ The substrate scope was investigated pertaining to both the acid chlorides and primary amines. Crucially, the addition of water allowed for precipitation of the product and facile isolation from the Cyrene solvent without the use of chromatography, see Scheme 9. Watson and co-workers have also evaluated the use of Cyrene in the HATU-mediated synthesis of amides and peptides.^119^ It was found that Cyrene was an effective replacement for DMF and NMP in the synthesis of lead-like molecules. Conditions were established that demonstrated a broad functional group tolerance and broad generality after evaluating 25 examples, with yields ranged from 63–100%, see Scheme 10. In 2017, Hunt and co-workers published an article comparing the utility of 2,2,5,5-tetramethyloxolane (TMO) in a series of reactions, one of being formation of an amide from phenylbutanoic acid and benzyl amine (Scheme 11), with traditional solvents such as toluene.^103^ In the studies, reaction kinetics analysis of the amide formation resulted in similar reaction rates to the effective toluene and *p*-cymene solvents, although the basicity (β) of TMO was similar to the less effective DMSO (Figure 28). Also in this report is discussed the nature of ethers containing α-protons to the oxygen to form dangerous peroxides in solution, upon irradiation with UV light. In a comparative irradiation experiment, traditional peroxide-forming ethers THF, 2-MeTHF, and diethyl ether formed varying amounts of detectable peroxide over a period of six months, whereas a solution of TMO stored under identical conditions formed no detectable peroxide.

**Scheme 12:**
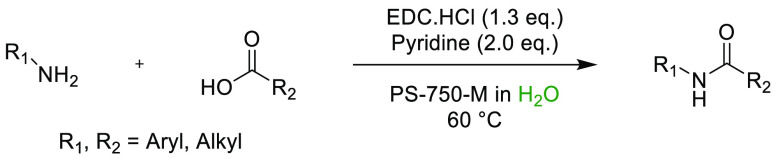
Fast Amide Coupling in a Surfactant–Water Mixture As Exemplified by Handa et al.^261^

Handa et al. have recently developed solvent-free methodology for fast and clean amide couplings in water that require no extraction, chromatography, or crystallization (Scheme 12).^261^ This is achieved by use of the l-proline-based amphiphile PS-750-M, developed by the group.^238^ In the micelle of PS-750-M, the presence of 3° amides from the surfactant proline linker structurally mimics DMF, NMP, and DMAc solvents. This allows extremely fast amide couplings, mediated by 1-ethyl-3-(3-(dimethylamino)propyl) carbodiimide (EDC), rather than expensive and specialized coupling reagents. The conditions developed by the group result in the precipitation of the product and isolation by filtration. The methodology is also reproducible on different reaction scales. Mechanistic and kinetic insights into PS-705-M mediated fast amide bond formation have also been provided.^262^

A similar approach to that of Handa et al. is that of the Lipshutz group, inventors of the α-tocopherol derived surfactant TPGS-750-M,^55^ who demonstrated in 2015 that amide bond formation can be rapidly conducted in TPGS-750-M–water systems in high yields. Products are extracted from the surfactant–water system using an organic solvent. The surfactant has been designed to be preferentially soluble in aqueous layers leading to facile product recovery and ease of recyclability of the surfactant–water phase (5 cycles conducted) and low E-factors (organic solvent + water E factor = 7.8).^263^ Reaction times were typically 1–4 h, and yields of amino acid amide couplings to give dipeptides (19 compounds) ranged from 84 to 99%. In 2013, Watson et al. published a solvent-reagent guide evaluating the use of greener alternatives to DMF and DCM for amide bond formation. It was shown that reaction rates and yields for alternative solvents such as dimethylcarbonate, EtOAc, and 2-MeTHF were comparable to DCM and DMF and could be considered as practical alternatives for both academic and industrial use. CPME was shown to be a poor alternative, most likely due to reaction mixtures becoming heterogeneous as they progressed.^264^

Amidation in water using *N*,*N*′-diisopropylcarbodiimide (DIC) as coupling reagent has been effectively demonstrated by Fattahi et al. with no requirement for surfactant additives.^265^ Both aromatic and aliphatic acids were shown to be compatible with this methodology.

**Scheme 13:**
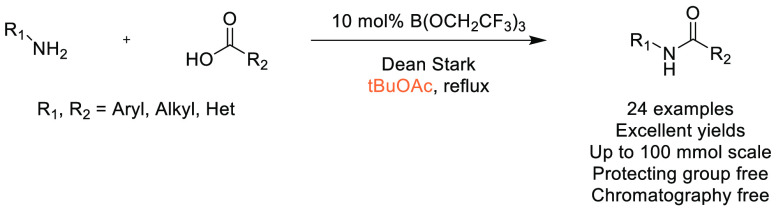
Catalytic, Protecting Group Free, Chromatography Free, Amide Bond Formation Using tBuOAc as Solvent^266^

Catalytic amide bond formation was reported by Coomber et al. in 2019 using a combination of B(OCH_2_CF_3_)_3_ in *tert*-butyl acetate, see Scheme 13. Significantly, this methodology allows for the coupling of unprotected amino acids in high yields. Scales of up to 100 mmol were demonstrated with product isolation achieved by resin catch and release protocols.^266^

Ultrafast amidation reactions using lithium amides under aerobic conditions and ambient temperatures have recently been reported by Fairley et al. under these conditions, amidation of ester starting materials. The reaction proceeds via C–O bond cleavage and is complete in just 20 s. Solvents employed were either 2-MeTHF or glycerol.^267^ High yields and good selectivity were generally observed making this methodology an advancement in accessing carboxamides by sustainable means via air and moisture compatible organometallic chemistry.

**Scheme 14:**
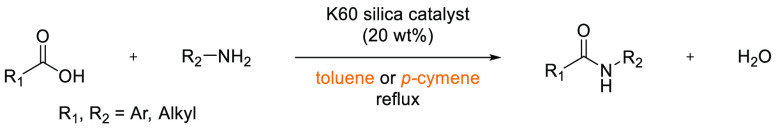
K60 Silica-Catalyzed Direct Amidation of Carboxylic Acids, Scoping the Utility of *p*-Cymene as a Substitute for Solvent of Concern Toluene

A further push toward substitution of regulated solvents used in amidation reactions with more sustainable biobased solvents, such as *p*-cymene, was demonstrated by Clark and co-workers from the University of York in 2018 (Scheme 14).^268^ Discovery of a preferred alternative to toluene for this transformation (which could also act as an alternative to dipolar aprotic solvents such as DMF) also removes concerns associated with reproductive toxicity.^269^ A direct comparison of yields for toluene and *p*-cymene in a silica-catalyzed amidation of carboxylic acids, across a varied substrate scope of 13 amides, was performed. In the majority of cases, *p*-cymene outperformed that of toluene when carried out under reflux (bp 177 °C). This methodology has further enhanced sustainability, with facile purification through amide precipitation, recycling of solvents, and use of a reusable solid-supported catalyst.

#### 3.1.1 Suzuki-Miyaura Cross-Coupling

**Scheme 15:**
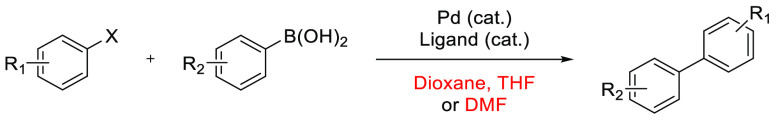
Generic Conditions for a Suzuki-Miyaura Cross-Coupling Reaction

Palladium catalysis is used extensively in both industrial and academic synthetic chemistry laboratories as a powerful methodology for the formation of C–C bonds. The Suzuki-Miyaura cross-coupling of organoboronic acids and their derivatives is the most frequently utilized palladium-catalyzed C–C bond forming reactions in the chemical industry, see Scheme 15.^14^ Traditionally, ethereal solvents such as 1,4-dioxane and THF or dipolar aprotics such as DMF are employed for a large proportion of these reactions.^14^ Owing to the frequent use and importance of this reaction, there is great potential for “greener” solvent alternatives to have a significant impact. Note that discussion surrounding the use of Suzuki-Miyaura reactions as a benchmark for the performance of new solvents suggests that it is not the most informative reaction manifold.^270^ For example, cross-coupling can be successfully conducted in rapeseed oil and butter with both of these lipid-based systems outperforming benchmark solvent DMF.^271^

**Scheme 16:**
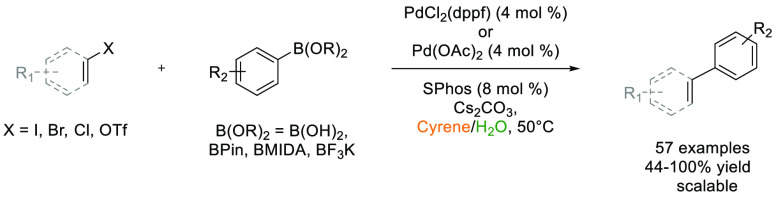
Suzuki Coupling Conducted in Bio-Based Solvent Cyrene^14^

Watson and co-workers developed a mild method for the Suzuki-Miyaura reaction that employed Cyrene as a greener alternative to the commonly used dipolar aprotic solvents (Scheme 16).^14^ The reaction scope was extensively explored by varying both the organoboron nucleophile and aryl electrophile. The conditions developed demonstrated excellent generality and functional group tolerance. The group reported 57 examples with yields ranging from 44 to 100%, from discovery to gram-scale reactions.

**Scheme 17:**
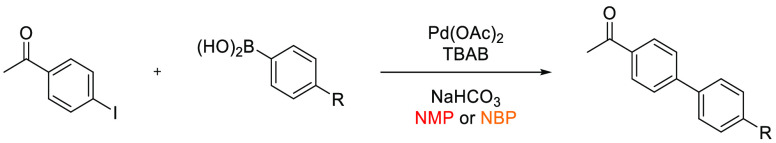
Suzuki Cross-Coupling Conducted in NMP Replacement, NBP^110^

Hunt et al. have explored the use of NBP as a dipolar aprotic solvent for Suzuki cross-coupling reactions, see Scheme 17.^110^ Yields for a series of reactions performed in both NMP and NBP have been reported, to carry out a comparison, using a range of different substituted aryl halides to explore the scope. Although the results showed that reactions using NMP as solvent gave better yields overall (76–90% for model reactions utilizing 4-iodoacetophenone and a range of phenylboronic acids), reactions carried out in NBP still gave good yields (∼10% lower for the same model reaction), as seen in Table 5. However, the reactions had not been optimized to specifically favor *N*-butyl-2-pyrrolidinone, so there may also be potential to improve the performance of these reactions.

**Table 5:** Suzuki Cross-Coupling Yield Comparison: NMP versus NBP^110^

entry	R group	conversion in NMP (%)	conversion in NBP (%)
1	H	83	73
2	CF_3_	76	72
3	NO_2_	89	79
4	OH	87	77
5	Me	90	81

Dimethyl isosorbide has been demonstrated as an effective reaction medium for several classical cross-coupling reactions. In 2018, Watson et al. reported the use of DMI in several Suzuki-Miyaura reactions (12 substrates). In general, aromatics with various electron donating and withdrawing groups were tolerated as were variations in the electrophilic component including vinyl groups, boronic acids, and pinacol esters.^214^ Reaction times were 1 h conducted at 60 °C with 4 mol % Pd(dppf)Cl_2_, and yields ranged from 62 to >99%.

**Scheme 18:**
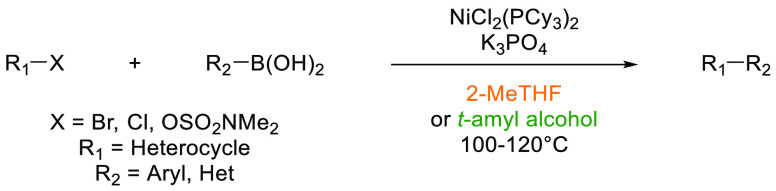
Nickel-Catalyzed Suzuki-Miyaura Cross-Coupling Conducted in Safer, More Sustainable Solvents^272^

Garg and co-workers have reported the nickel-catalyzed Suzuki-Miyaura cross-coupling between aryl halides and (hetero)aromatic boronic acids in a number of greener solvent alternatives.^272^ Initial solvent screening showed that EtOAc, iPrOH, 2-MeTHF, and *t*-amyl alcohol were all demonstrated to be excellent alternatives, see Scheme 18, to traditionally employed solvents such as toluene or 1,4-dioxane. The reaction scope was further explored using 2-MeTHF and *t*-amyl alcohol as solvents. The scope was found to be broad pertaining to both coupling partners, with heterocycles well tolerated (Scheme 18). The methodology was demonstrated on multigram scales using low catalyst loadings (0.5–1.0 mol % nickel) to provide desired products in reasonable to excellent yields. 2-MeTHF has also been successfully employed as reaction solvent in palladium–NHC-catalyzed amide and ester cleaving cross-coupling reactions.^273^

Following the emergence of eucalyptol as a sustainable, bioderived solvent, the utility of it in cross-coupling reactions has been explored in a number of transformations by Berteina-Raboin and colleagues.^205−207^ When tested in palladium-catalyzed Suzuki-Miyaura cross-coupling reactions of arylboronic acids with heteroaryl chlorides, eucalyptol outperformed reactions conducted in THF, DMF, and 1,4-dioxane, with yields ranging from 44 to 99%. Reaction temperatures of 100 °C were required for complete dissolution of reagents.

#### 3.1.2 Mizoroki-Heck Cross-Coupling

**Scheme 19:**
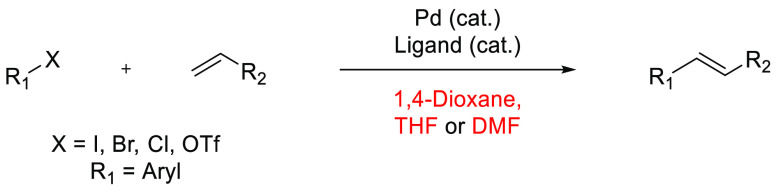
Generic Mizoroki-Heck Cross-Coupling Conditions

The Mizoroki-Heck cross-coupling, commonly referred to as the Heck cross-coupling, involves the reaction of an aryl halide with an alkene under Pd(0)/Pd(II) catalysis. Common reaction solvents for the Heck reaction include 1,4-dioxane, THF, and amide-based solvents such as DMF and DMAc, see Scheme 19. Although there are examples using more sustainable solvents such as water and acetonitrile, expansion of the available green solvents for the transformation is underway.^110,214^

**Scheme 20:**
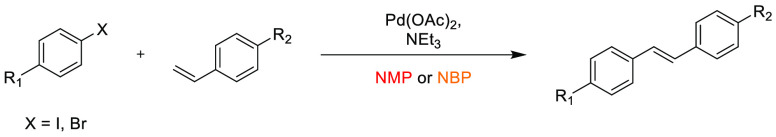
Mizoroki-Heck Reactions Conducted Using NMP Drop-in Replacement NBP^110^

Hunt et al. have explored the scope and limitations of the use of NBP as a dipolar aprotic solvent for the Heck cross-coupling reaction, see Scheme 20, in addition to Suzuki-Miyaura couplings.^110^ The group reported and compared the yields from a series of reactions performed in both NMP and NBP. In summary, they found that the Heck reactions carried out in NBP gave comparable to or higher yields than those carried out in NMP, see Table 6. This comparison has demonstrated that NBP was a viable replacement to NMP as reaction solvent in the Heck cross-coupling reactions examined.

**Table 6:** Mizoroki-Heck Cross-Coupling Yield Comparison: NMP versus NBP^110^

entry	X	R	R′	conversion in NMP (%)	conversion in NBP (%)
1	I	H	H	91	92
2	I	H	Me	96	94
3	I	H	OMe	89	93
4	I	H	2-vinylnaphthalene	>99	>99
5	I	H	CF_3_	94	90
6	I	Cl	H	68	85
7	I	Cl	Me	>99	>99
8	Br	CN	H	85	87
9	Br	CN	Me	>99	99

Dimethyl isosorbide has also been demonstrated as an effective reaction medium for the Mizoroki-Heck reaction by Watson et al.^214^ Optimized reaction conditions using 5 mol % Pd(dppf)Cl_2_ were trialled on 12 substrates and showed that the reaction proceeded well with aryl iodides and bromides facilitating coupling of both electron poor and rich arenes with a variety of vinyl ketones and acrylamides in 47–91% yield. Reactions were generally conducted at 80 °C for 1 h though some aryl bromide examples required heating at 115 °C for 24 h.^214^

**Figure 29:**
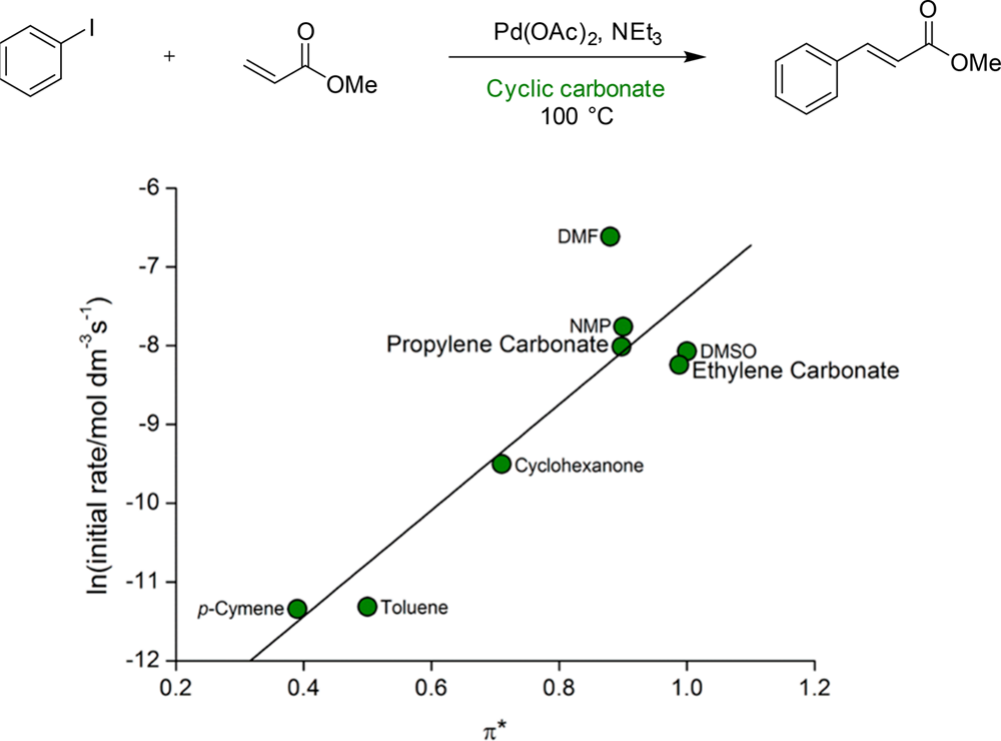
Relationship between solvent dipolarity and the natural logarithm of the initial reaction rate. Reprinted with permission from ref (107). Copyright 2014 American Chemical Society.

Another alternative class of solvents shown to have utility in Mizoroki-Heck cross-couplings reactions is that of cyclic carbonates, see Figure 29. In a study evaluating the use of a selection of dipolar aprotic solvents for the transformation, Clark and colleagues demonstrated the effective use of propylene and ethylene carbonate in the place of NMP and DMSO.^107^ In many cases, both cyclic carbonate solvents performed as or more effectively than NMP across a wide substrate scope, tolerating substrates of various electronics.

**Scheme 21:**
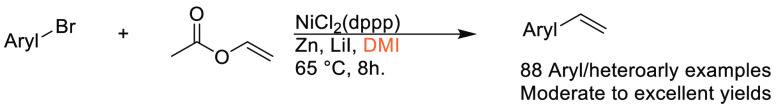
Nickel-Catalyzed Reductive Cross-Coupling Conducted in DMI

Recently, Su et al. have demonstrated that DMI could be effectively utilized in a Ni-catalyzed reductive cross-coupling of aryl bromides with vinyl acetate (acting as a vinylating reagent).^274^ Moderate to excellent yields of the vinylated products were observed across a wide and varied heteroaryl substrate scope, see Scheme 21.

#### 3.1.3 Sonogashira Coupling

**Scheme 22:**
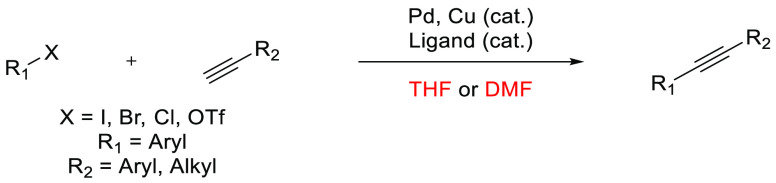
Generic Conditions for Sonogashira Cross-Coupling Reaction

The Sonogashira cross-coupling is a widely used palladium and copper-catalyzed process for accessing functionalized alkyne species from terminal alkynes and aryl halides, see Scheme 22.^275^ Another powerful C–C bond forming reaction, the Sonogashira reaction is commonly performed in THF and DMF,^276^ both of which are problematic due to peroxide formation and reprotoxicity, respectively. Because of the efficiency and widespread use of this reaction in both academia and industry, a search for greener solvent alternatives is highly desirable.

**Scheme 23:**
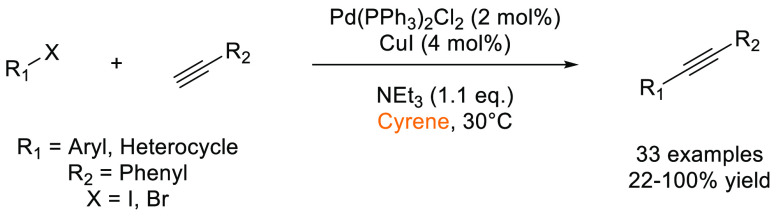
Sonogashira Reactions Conducted Using Bio-Based Solvent Cyrene^127^

**Scheme 24:**
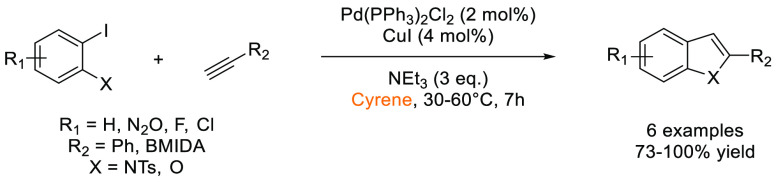
Cacchi-type Annulations to Produce Benzofuran and Indole Scaffolds Using Cyrene as Solvent^127^

Cyrene has been successfully utilized by Watson et al. as reaction solvent for Sonogashira cross-coupling reactions, see Scheme 23.^127^ Utilization of the safer, more sustainable Cyrene as an alternative to traditionally employed DMF^127^ was demonstrated across a number of aryl and heteroaryl iodides and alkyne coupling partners. Broad tolerance to the optimized reaction conditions was observed with yields of 65–99% generally reported, though yield variability was observed for some electron deficient aryl bromides (28–99%). Further expansion of the methodology to Cacchi-type annulations furnished benzofuran and indole scaffolds in good to excellent yields (73–100%), see Scheme 24.

Dimethyl isosorbide has been successfully utilized as reaction solvent for the Sonogashira reaction by Watson et al. Twelve substrates were synthesized using the same catalyst and base conditions as depicted in Scheme 23 (reactions were conducted at 25 °C).^214^ A range of aryl iodides and bromides including electron rich and deficient examples, and a number of functionalized alkynes including BMIDA and TIPS functionalities, were synthesized in 83–98% yields. One example of a thiophenol derived alkyne was synthesized, though in a lower yield of 65%.^214^

**Figure 30:**
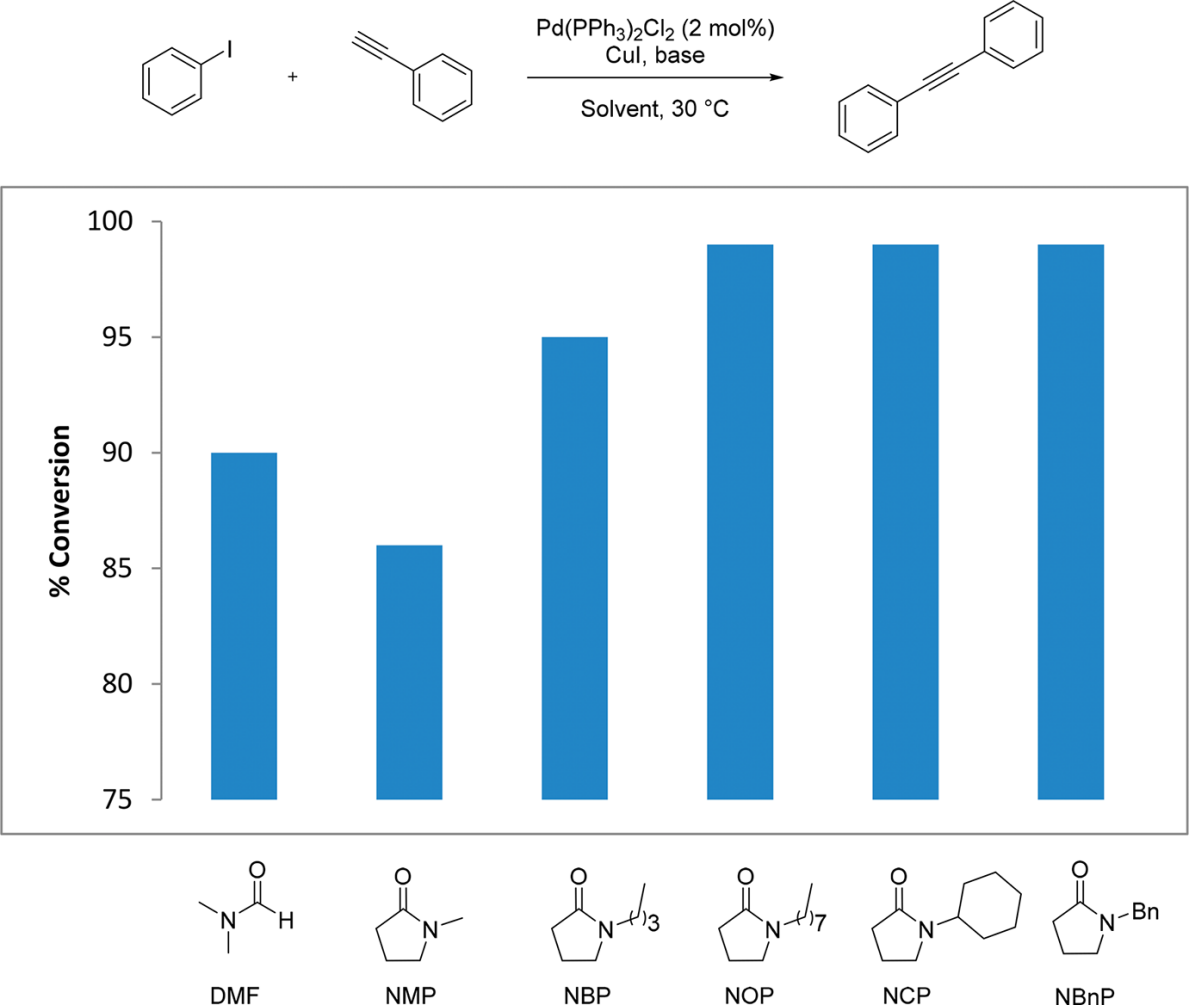
Yield comparison for model Sonogashira reaction conducted by Cabri et al. using a variety of NMP/DMF drop-in replacements.^276^

Cabri and co-workers have recently published an investigation into the use of *N*-substituted-pyrrolidinone solvents, along with a small number of others, as a reaction medium for the Sonogashira coupling.^276^ Given the knowledge of the reprotoxicity problems associated with NMP,^17^ the safer and more sustainable species discussed, such as NBP (*N*-butyl-2-pyrrolidinone) and NCP (*N*-cyclohexyl-2-pyrrolidinone), were shown to outperform DMF and NMP following optimization of reaction conditions. In addition to the broad substrate scope (generally >90% isolated yields), the methodology was applied to the synthesis of Erlotinib, performing a Sonogashira cross-coupling step in quantitative yield, without the need for a CuI additive, see Figure 30.^276^

In addition to the previously mentioned explorations of the utility of eucalyptol as a solvent in palladium-catalyzed processes, it was also shown to be effective in Sonogashira cross-couplings.^277^ A slightly different system to regular Sonogashira cross-couplings, utilizing Pd(PhCN)_2_Cl_2_ and PCy_3_, without addition of CuI, furnished a range of cross-coupled products in moderate to good yields.^277^ It was noted that the cross-coupling of any heterocycles containing free amine functionality, such as 4-chloro-7*H*-pyrrolo[2,3-*d*]pyrimidine is ineffective when using eucalyptol as the solvent; further investigations are required to optimize this process such that yields are comparable to those obtained using traditional Sonogashira solvents such as DMF.

#### 3.1.4 Buchwald-Hartwig Amination

**Scheme 25:**
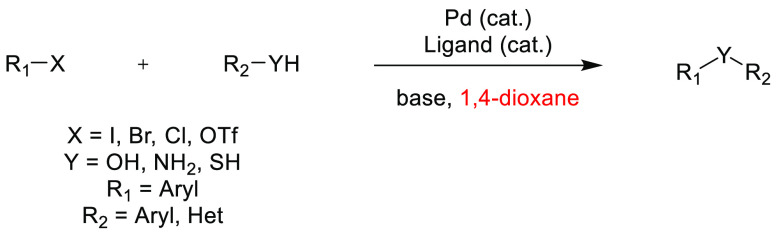
Typical Conditions for Buchwald-Hartwig Coupling of Nucleophiles and Aryl Halides

Buchwald-Hartwig amination is a widely employed cross-coupling strategy used for the formation of C–N bonds, see Scheme 25. Commonly used for substrates that are less reactive to nucleophilic aromatic substitution, the palladium-catalyzed transformation is also known to perform well with oxygen and sulfur nucleophiles.^278,279^ 1,4-Dioxane is commonly utilized as reaction solvent for these transformations, and 1,4-dioxane has serious issues and is regarded as a solvent of concern.^280^

**Scheme 26:**
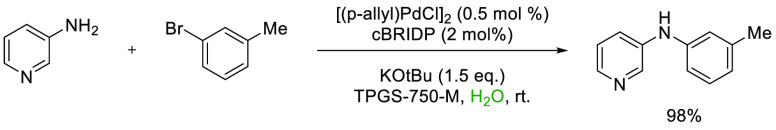
Example Buchwald-Hartwig Amination Conducted in a Surfactant–Water Mixture

A number of strategies to overcome this issue include reactions conducted solvent-free,^281^ in water,^282^ or using water–surfactant systems.^55^ In 2014, the Lipshutz group published methodology describing Buchwald-Hartwig amination using the TPGS-750-M surfactant–water system, see Scheme 26.

**Scheme 27:**
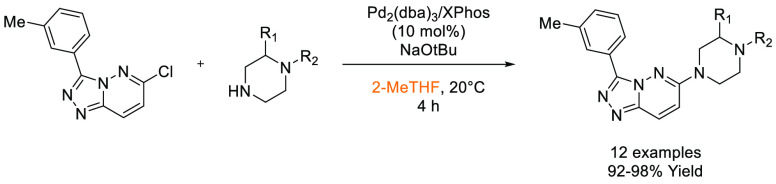
Final Stage Buchwald-Hartwig Aminations in 2-MeTHF to Access Anti-diabetic Compounds^283^

In 2020, Manikandan and co-workers opted for the use of 2-MeTHF in the synthesis of a triazolo-pyridazine-6-yl-substituted via a Buchwald-Hartwig cross-coupling.^283^ The 2-MeTHF was used as the solvent in the final stage of the synthesis for 12 medicinally relevant antidiabetic compounds, and yields were reported in the range of 92–98% using a catalytic combination of 10 mol % Pd_2_(dba)_3_/XPhos, in the presence of NaO*t*Bu at room temperature for 4 h, see Scheme 27.

The use of a surfactant however is not always necessary, and aqueous single or two-phase systems have been known in the Pd-catalyzed amination manifold.^284^ Further exploration into aqueous systems has also been conducted and was well documented by Buchwald et al. in 2003.^285^

Water has also been successfully utilized as reaction solvent in related Cu-catalyzed Ullman-type reactions,^286^ as have other safer solvents such as tBuOH.^287^ Solvent-free examples are also known.^288^

**Scheme 28:**
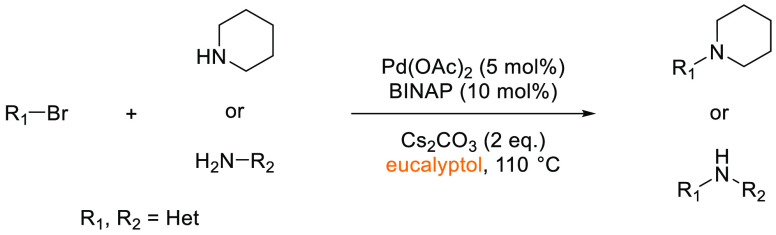
Buchwald-Hartwig Amination Reactions Conducted in Bio-Based Solvent Eucalyptol

Bio-based solvent eucalyptol has also been studied for its potential use as solvent in the Buchwald-Hartwig amination, see Scheme 28. A number of model reactions were examined encompassing a variety of bromoarenes and amines and anilines. Moderate to excellent yields were obtained (43–99%), in many cases better than the reported yields for the analogous reaction conducted in a conventional dipolar aprotic solvent.^289^

**Scheme 29:**
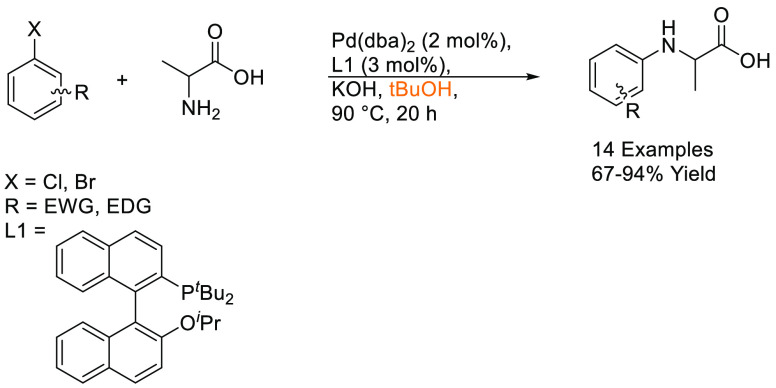
Pd-Catalyzed C–N Bond Formation of Amino Acids Using *t*BuOH as Solvent^290^

Ma et al. have demonstrated that *t*BuOH could be effectively used as an alternative to 1,4-dioxane in the cross-coupling of amino acid esters with aryl bromides and chlorides, see Scheme 29.^290^ The methodology was not just limited to α-amino acids but also included β-, γ-, and δ-, proteinogenic, and nonproteinogenic amino acids. Products were prepared generally in moderate to high yields with retention of optical activity. Other examples utilizing *t*BuOH in Pd catalyzed C–N have also been reported,^291^ and alcohols, such as *i*PrOH, have also seen use in Pd-catalyzed C–N and C–O bond forming reactions.^292^ Solvent-free, ball milling conditions have been described by Shao et al. in 2018.^293^

**Scheme 30:**
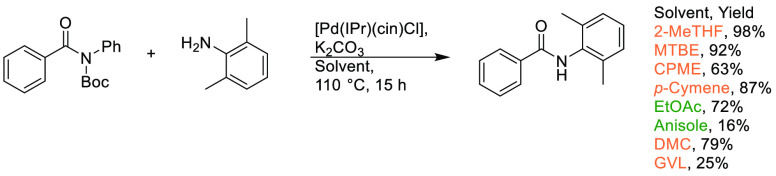
Transamidation Conducted in Alternative Solvents

A solvent selection guide for conducting acyl Buchwald-Hartwig transamidation reactions has recently been compiled by Lei et al. In their study, an exhaustive solvent screen of alternative solvents has been conducted including 2-MeTHF, CPME, GVL, anisole, and *p*-cymene, see Scheme 30.^294^ 1,2-Dimethoxyethane was used as a benchmark solvent, giving 86% yield in the same transformation. Both 2-MeTHF and MTBE showed excellent generality and applicability across a wide range of transamidation coupling partners demonstrated through a substrate screen consisting of a variety of anilines and amides bearing EDGs and EWGs. Thus, dipolar aprotics of concern in these reactions such as THF or 1,2-dimethoxyethane can be effectively replaced by a drop-in alternative.^294^

#### 3.1.5 Borylation Chemistry

**Scheme 31:**
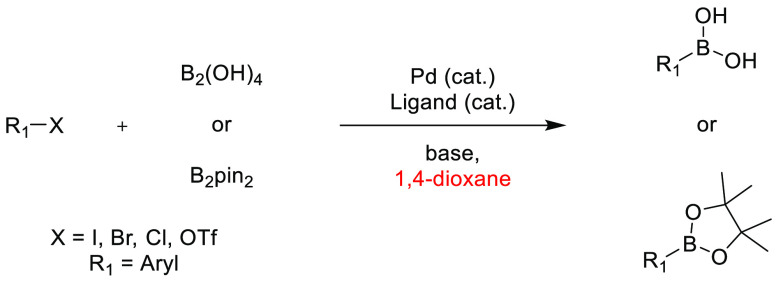
Generic Conditions Employed for Miyaura Borylation Reactions

Because of the widespread use of the Suzuki-Miyaura reaction, and variants thereof, in synthetic and medicinal chemistry,^14^ and the requirement for a boronic acid or boronate coupling partner, sustainable methods for accessing these moieties are highly desirable. Traditional methods for preparation of boronic acids and boronates, such as the Miyaura borylation (Scheme 31), require dipolar aprotic solvents such as DMSO, DMF, and 1,4-dioxane.^295^

**Scheme 32:**
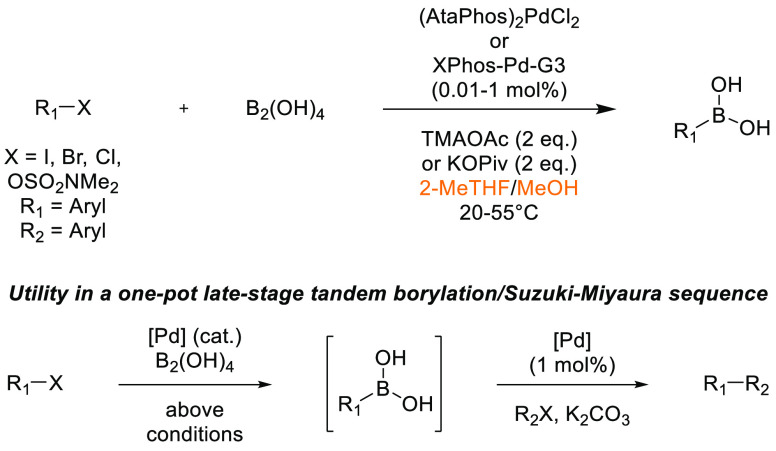
Miyaura Borylation Reactions Conducted Using 2-MeTHF/MeOH Mixtures^296^

Recently, advancements have been made toward more sustainable solvents for borylation reactions, with cyclic ethers and cyclic ketones being used as substitutes. A recent publication by Frantz and co-workers describes a sustainable method for accessing arylboronic acids from pseudohalides and B_2_(OH)_4_ using a 0.01 mol % palladium catalyst loading in a 1:1 mixture of 2-MeTHF and MeOH, see Scheme 32.^296^ Also described is a direct one-pot conversion of aryl halide to the boronic acid diethanolamine (DEA) adduct, providing synthesis of boronate species less susceptible to protodeboronation.^297^ This transformation is well-tolerated for aryl and (hetero)aryl iodides, bromides, and chlorides, with yields ranging from 60–85%, on the choice of catalyst, (AtaPhos)_2_PdCl_2_ or XPhos-Pd-G3. The effectiveness of the methodology is demonstrated by a one-pot tandem borylation-Suzuki sequence, furnishing a diverse range of C–C coupled products with no more than 2 mol % palladium used for the overall two-step process. Many more examples of greener palladium-catalyzed borylations of aryl halides using B_2_(OH)_4_ or B_2_pin_2_ are known in 2-MeTHF^298^ and CPME.^299^

**Scheme 33:**
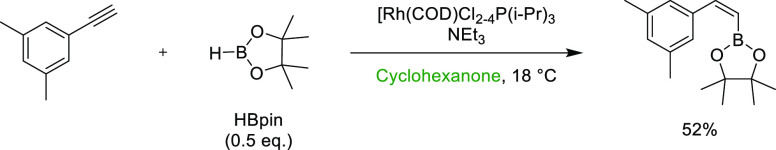
Rhodium-Catalyzed Hydroboration Conducted Using Cyclohexanone as Solvent^301^

Although not as widely used as aryl and hetero(aryl)boronic acids and boronates, alkenyl boronates have important applications in Diels–Alder cycloadditions, as precursors to cyclopropyl boronates, and as effective radical acceptors.^300^ Recently, a number of publications have highlighted the use of sustainable solvents in borylation reactions of alkynes, both rhodium^301^ and copper-catalyzed.^302^ A publication by Hutzler and co-workers in 2017, outlining routes to the synthesis of herbicides, included a rhodium-catalyzed hydroboration of alkynes with HBpin, with a solvent choice of cyclohexanone.^301^ The reaction, carried out below room temperature (18 °C), yielded the desired *Z*-alkene in 52% yield after 2 h (Scheme 33).

**Scheme 34:**
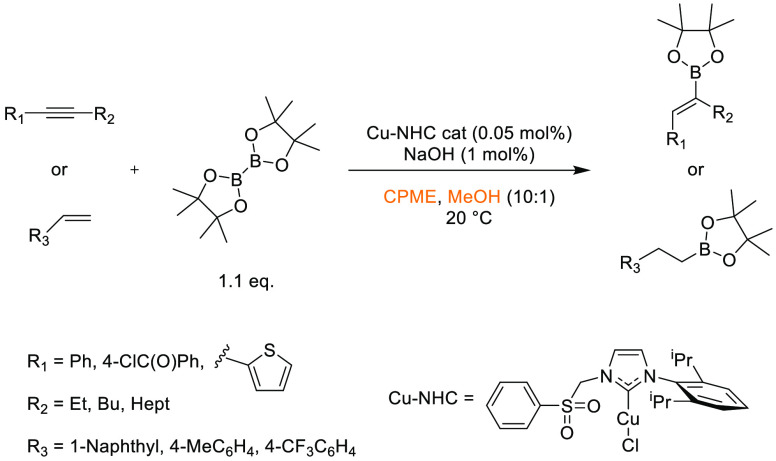
Synthesis of Alkenyl Boronates via NHC Complexes in Alternative Solvent System CPME:MeOH 10:1^302^

Similarly, a publication by Woźniak and co-workers in 2019, showcasing the utility of copper-NHC complexes containing sulfone and sulfoxide side-chains, describes a low catalyst loading (0.05 mol %) hydroboration of alkynes, using CPME as the solvent, with a MeOH additive (10:1), see Scheme 34.^302^ In this instance, CPME outperforms both isopropanol and toluene, and the catalytic system applies to both alkynes (yielding alkenyl boronates) and alkenes (yielding alkyl boronates). Yields for all substrates studied are in excess of 95%, proving the methodology a very powerful and sustainable way to hydroborate alkynes and alkenes. In addition to solvent swaps to CPME, 2-MeTHF, and cyclohexanone, MTBE has also been shown to be highly effective in borylation sequences. Reported in 2009, Marder and co-workers utilized the solvent in a one-pot C–H borylation/Suzuki-Miyaura cross-coupling sequence, a transformation shown to be highly effective for both aryl and heteroaryl substrates.^174^ Borylation in protic solvents such as MeOH^303^ and EtOH^304^ has also been reported including in the 2020 report on transition metal-free borylation of aryl halides using bis-boronic acid in MeOH.^305^

#### 3.1.6 C–H Activation

**Scheme 35:**
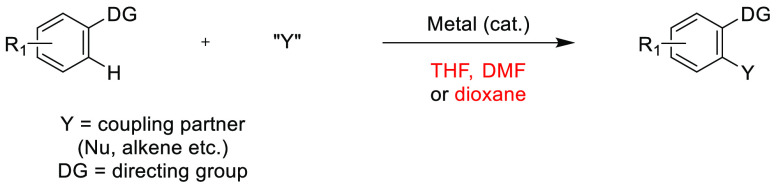
Reaction Scheme for Generic C–H Activation Functionalization

The direct activation and functionalization of C–H bonds, which avoids the installation of intermediate functional groups, is both step- and atom-efficient, see Scheme 35. However, the sustainability of C–H activation is hindered (especially for large-scale chemistry) by numerous factors, one of these being the introduction of safer, greener, and more sustainable solvents. In many examples of chemistry involving C–H activation, green solvents have been too easily overlooked in preference to solvents such as THF, DMF, and 1,4-dioxane, which have been frequently used.^306^ The inclusion of safer solvents such as 2-MeTHF (and exploring less conventional PEG or aqueous-micellar systems) during reaction optimization and solvent screening is therefore critical if the field of C–H activation is to migrate away from solvents of concern. Reviews on the subject of emerging unconventional organic solvents for C–H bond activation have been published in 2019 by Gandeepan et al.,^307^ in 2020 by Yu et al.,^308^ and in 2021 by Dhawa et al.^309^ and Dalton et al.^306^ We direct the reader toward these bodies of work for further information and specific examples.

**Scheme 36:**
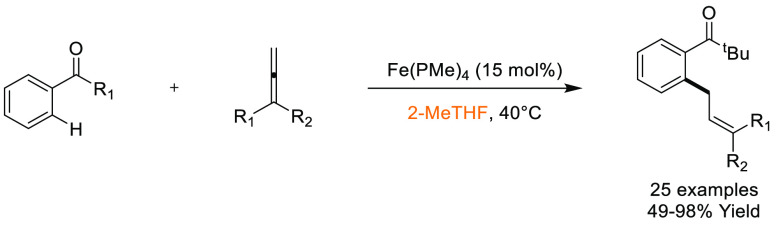
C–H Activation of Allenes Using a Fe(0) Phosphine Catalyst^310^

The 2-MeTHF was used as a replacement to THF and 1,4-dioxane by Ackerman and co-workers in a mild C–H activation of allenes using a Fe(0) phosphine catalyst (Scheme 36).^310^ Across a selection of aryl substrates containing an α-ketone directing group, efficient C–H functionalization using Fe(PMe_3_)_4_ yielded a diverse range of products in good to excellent yields. Also demonstrated was a tolerability of hydroxyl, amino, and alkoxycarbonyl moieties, in addition to mechanistic and computational investigations.

**Scheme 37:**
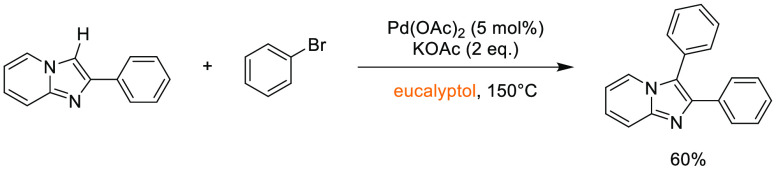
Utilizing Eucalyptol as Solvent in Pd-Catalyzed C–H Activation^277^

The bioderived solvent eucalyptol has also been shown to have utility as a solvent for direct C–H arylations by Berteina-Raboin and colleagues.^277^ Following synthesis of imidazo[1,2-*a*]pyridines from the corresponding 2-aminopyridine and 2-bromoacetophenone derivatives, Pd(OAc)_2_-catalyzed direct C–H arylation at the C-3 position using bromobenzene in eucalyptol yielded the desired arylated product in 60% (5 mol % cat.) and 61% yields (10 mol % cat.) after 24 h at 150 °C, see Scheme 37. Other bio-based solvents such as cumene have also been promoted as a potential solvent for C–H bond activation chemistry.^308^

**Scheme 38:**
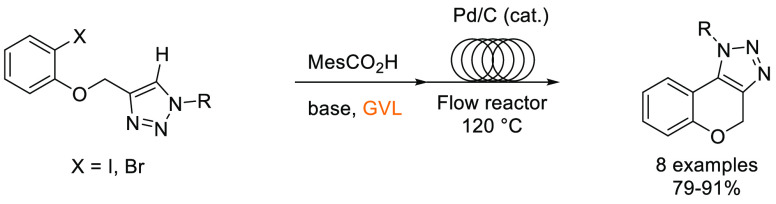
Regioselective C–H Functionalization of 1,2,3-Triazoles in GVL in Continuous Flow^311^

A regioselective C–H functionalization of 1,2,3-triazoles in GVL was accomplished by Vaccaro and co-workers in continuous flow, see Scheme 38. Across 8 substrates, excellent yields (79–91%) of the cyclized product were obtained, with solvent recovery and reuse, without further purification, also demonstrated.^311^

**Scheme 39:**
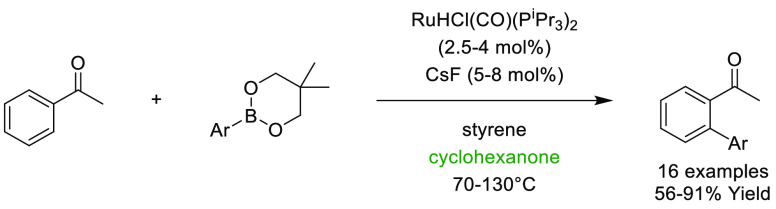
Ru-Catalyzed C–H Activation in Cyclohexanone Solvent

Kakiuchi and co-workers developed a Ru-catalyzed stereoselective C–H arylation of ortho-methoxylated acetophenones and C–H monoarylation of ortho-unsubstituted acetophenones in high yields using cyclohexanone as solvent, see Scheme 39. Note: styrene was utilized as a necessary additive to selectively provide monoarylation.^312,313^ Aqueous surfactant systems such as TPGS750M-H_2_O have also been successfully utilized in ruthenium-catalyzed C–H arylation reactions.^314^

**Scheme 40:**
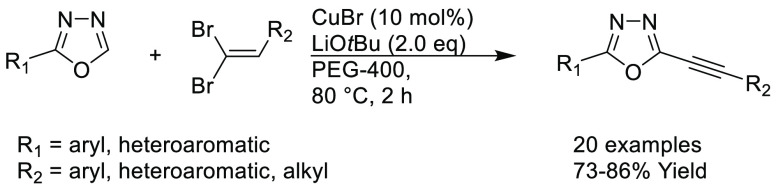
Cu-Mediated Cross-Coupling Using PEG-400 as Solvent

PEG-based systems have been demonstrated as promising alternatives to conventional solvents as evidenced by Ackermann et al., who first showed in 2009 that PEG-20000 could be used as solvent for either Ru- or Pd-catalyzed direct arylation chemistry.^315^ This discovery has led to other PEG-derived systems such as that demonstrated by Reddy et al.^316^ who showed that PEG-400 was an excellent solvent for Cu-mediated cross-coupling of oxadiazoles with dibromoalkenes, see Scheme 40. When conventional solvents such as CH_3_CN and DCM were screened instead of the PEG, no reaction took place.

#### 3.1.7 Boc Deprotection

**Scheme 41:**
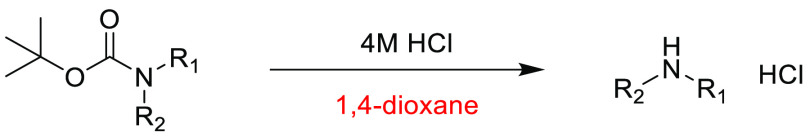
Generic Boc Deprotection Transformation

The *t*-butyl carbamate (Boc) group is a common protecting group for amines, most often utilized in multistep syntheses due to its stability under a wide range of reaction conditions. One of the most common methods of deprotection typically employs 4 M HCl in 1,4-dioxane, a solvent it would be good to replace when possible, see Scheme 41.

Commercially available alternatives to 1,4-dioxane preparations of HCl are available in safer, more sustainable solvents including ethereal alternative CPME.^317^ Given the increasing availability of HCl solutions in more sustainable solvents, it is recommended that alternative mixtures are considered before opting to use HCl in 1,4-dioxane. Several examples throughout the literature demonstrate the utility of more sustainable methods for Boc deprotections, as part of lengthy syntheses.

**Scheme 42:**
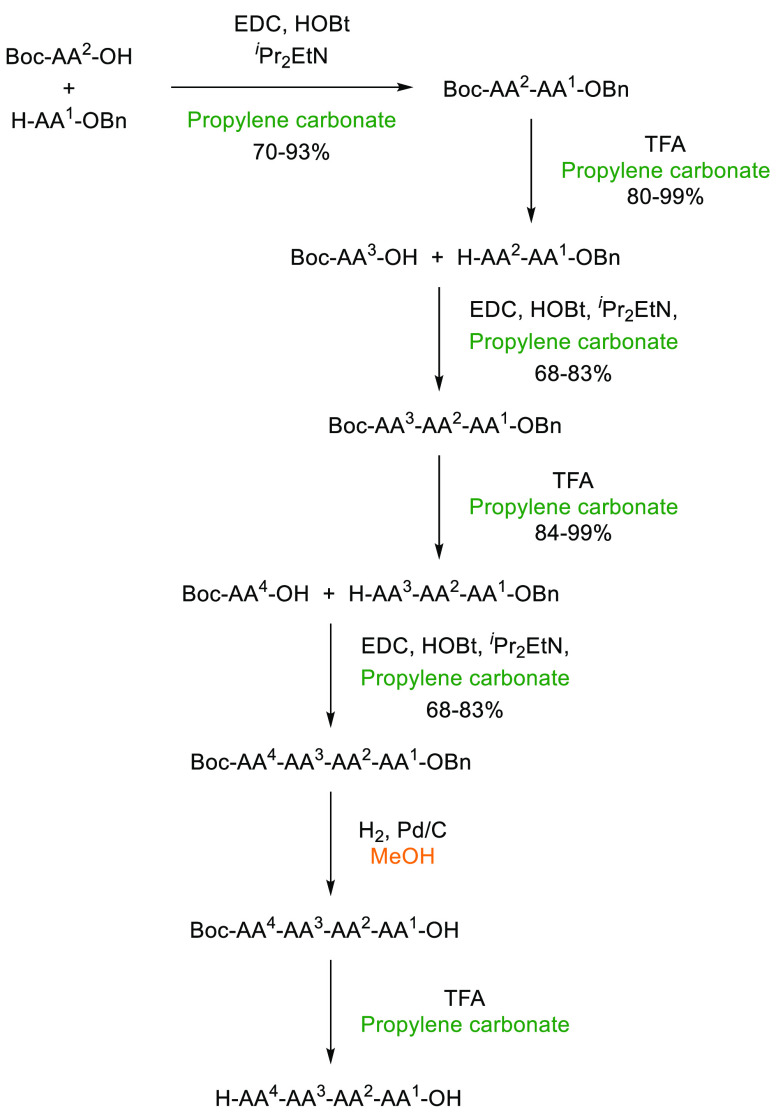
TFA-Mediated Boc Deprotection in a More Sustainable Solvent, Propylene Carbonate^319^

As part of the design and testing of a sustainable methodology for an improved Pinner reaction in CPME, Torisawa and co-workers discuss the use of a 4 M solution of HCl in CPME for the deprotection of a Boc-protected amino acid derivative.^318^ Stirring of the derivative for 6 h at 0 °C provided the desired deprotected amine in 97% yield, with the workup procedure also employing the greener solvent. A common alternative acidic medium for the deprotection of Boc groups involves the use of CF_3_CO_2_H, trifluoroacetic acid (TFA). The use of TFA within a sustainable solvent was demonstrated as part of a peptide coupling by North et al. in 2017, in which the final Boc deprotection step used a solution of TFA in propylene carbonate, see Scheme 42.^319^ Use of this combination provided sequential deprotection yields between 80 and 99% yields in the three individual Boc deprotection steps in the multistep synthesis of tetrapeptides.

**Scheme 43:**
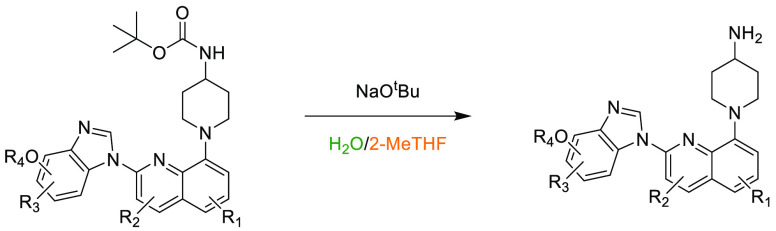
NaOtBu-Mediated Boc Deprotection in a Water/2-MeTHF Mixture^158,320^

An additional example demonstrating the deprotection of a Boc group was reported in a Pfizer patent in 2005.^320^ Instead of using TFA or HCl, a biphasic mixture of sodium *tert*-butoxide in H_2_O/2-MeTHF provided improved conditions for the deprotection of a range of heterocyclic substrates with medicinal applications, see Scheme 43.^158^

#### 3.1.8 Carbonylations and Carboxylations

**Scheme 44:**
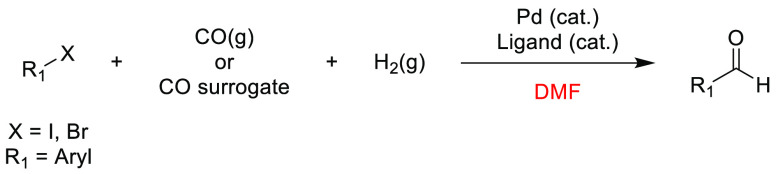
Formylation of Aryl Halides

**Scheme 45:**
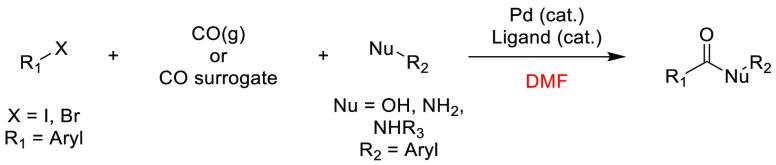
Aminocarbonylations and Carbonylative Etherifications of Aryl Halides

**Scheme 46:**
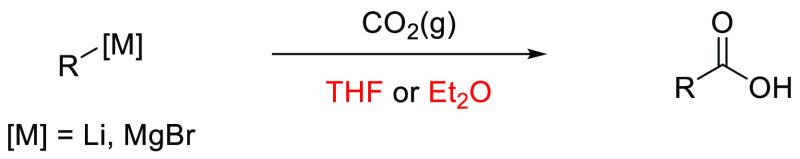
Carboxylations of Organometallic Reagents

Aldehydes, amides, esters, and carboxylic acids are abundant groups in synthetic organic chemistry, whether as part of a reaction intermediate or pharmaceutically relevant scaffold. Traditional methods for the preparation of such species include hydroformylations of alkenes and amide couplings, to name a few, which often require toxic heavy metals,^321^ pyrophoric organometallic reagents,^322^ or allergenic amide coupling reagents.^323^ In recent years there has been a shift toward catalytic methodologies including palladium-catalyzed formylations and carbonylations of aryl halides using carbon monoxide gas or surrogates thereof.^324^ Unfortunately, use of the gas is often accompanied by the use of dipolar aprotic solvents such as DMF,^325^ see Schemes 44 and 45, or 1,4-dioxane,^326^ see Scheme 46, to aid the solubility of the gas in the reaction medium.^327^

**Scheme 47:**
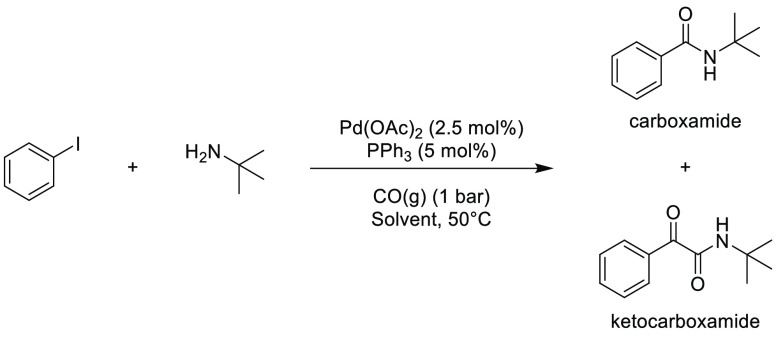
Solvent Screening for a Model Aminocarbonylation Reaction^328^

Recently, there has been interest in substituting reprotoxic dipolar aprotic solvents with more sustainable alternatives for carbonylation reactions. One of the alternative solvents for such transformations has been the cellulose-derived solvent GVL. In 2016, initial exploratory work into the use of biomass-derived solvents by Mika and colleagues revealed the utility of GVL in the aminocarbonylation of aryl halides under CO(g), see Scheme 47.^328^ In a system catalyzed by Pd(OAc)_2_/PPh_3_, it was shown that a mixture of the carboxamide and ketocarboxamide products could be obtained, and relative ratio of the ketocarboxamide could be favored by increasing of CO(g) pressure, see Table 7.^328^

**Table 7:** Yield and Selectivity Results for Aminocarbonylation Reaction Depicted in Scheme 47

solvent	carboxamide (%)	ketocarboxamide (%)
DMF	42	58
1,4-dioxane	68	32
GVL	30	70

**Scheme 48:**
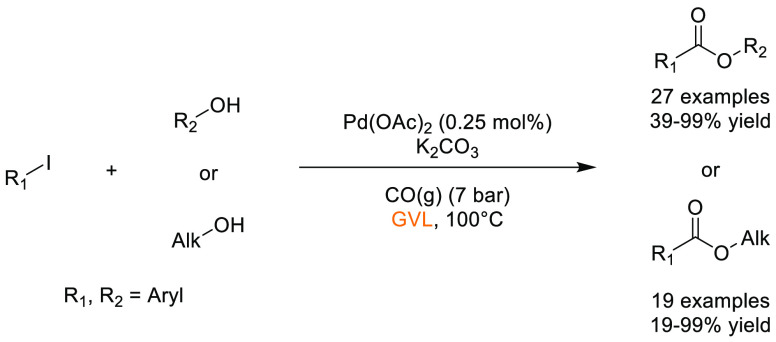
Pd-Catalyzed Carbonylative Esterification Using GVL as Solvent^329^

Following on from this initial work, the group later published a palladium-catalyzed carbonylative esterification of aryl iodides with a wide range of alcohols in 2020.^329^ Again using GVL, the transformation provided a wide range of ester products with significantly improved yields compared to the previous aminocarbonylation,^328^ ranging from 39 to 99% with phenol coupling partners and 13–99% with alkyl alcohol coupling partners, see Scheme 48.^329^

**Scheme 49:**
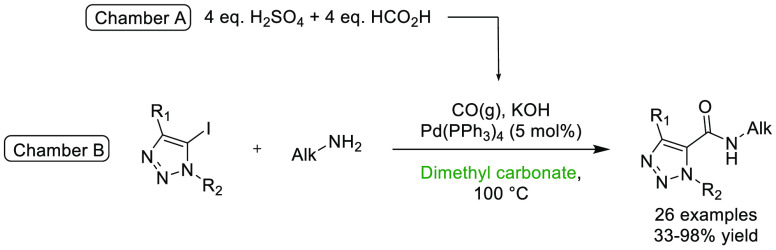
Synthesis of a Variety of 1,2,3-Triazole-5-carboxamides Using Dimethyl Carbonate as Solvent^330^

In 2019, Schwab and co-workers reported a simple and efficient aminocarbonylation process to access 1,2,3-triazole-5-carboxamides from the related heteroaryl iodide species.^330^ The Pd(PPh_3_)_4_-catalyzed process involved *in situ* generation of CO(g) using a combination of H_2_SO_4_ and HCO_2_H within CO-ware equipment. Utilizing the sustainable solvent dimethyl carbonate, a variety of functionalized 1,2,3-triazole-5-carboxamides were produced, with yields ranging from 33 to 98% (Scheme 49).^330^

**Scheme 50:**
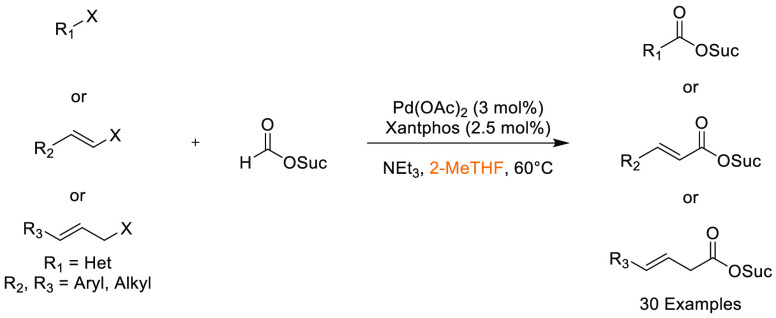
Carboxylation Reactions Conducted in 2-MeTHF^331^

In the area of carboxylation reactions, in 2015, Levacher and co-workers reported a sustainable carboxylation of organic (heteroaryl, alkenyl, and allyl) halides in 2-MeTHF, see Scheme 50.^331^ Using *N*-hydroxysuccinimidyl formate as a CO_2_(g) surrogate, and in the process eliminating the requirement for a high CO_2_-solubilizing dipolar aprotic solvent and opting for the use of 2-MeTHF, a wide range of *N*-hydroxysuccinimide esters were accessible (Scheme 50). The methodology was shown to be effective in the carboxylation of a functionalized quinoline intermediate in the preparation of a number of acetylcholinesterase (AChE) inhibitors, with the transformation progressing in 82% isolated yield.^331^

**Scheme 51:**
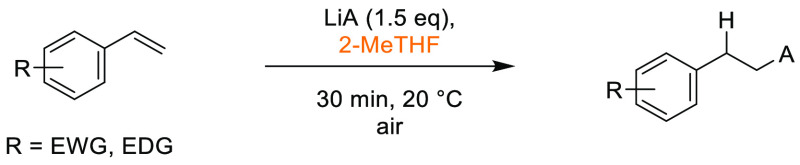
Alkali-Metal-Amide Hydroamination Reactions under Air Using 2-MeTHF as Solvent^332^

Metal-catalyzed hydroamination of styrenes has recently been reported by Mulks et al., see Scheme 51. In their work, the conventional wisdom of air- and moisture-free organometallic reactions is challenged by their demonstration of hydroamination of aryl alkenes in 2-MeTHF using lithium amides, see Table 8. Reactions were open to air and moisture and stirred at room temperature for 30 min. Yields were moderate to excellent with some incompatibility observed when using styrenes containing aryl halide moieties.^332^ Remarkably, the presence of adventitious moisture was shown to be beneficial and not detrimental. Reactions were accelerated by moisture, which generated free amine *in situ* at a steady rate.^332^ Sodium amides were also shown to be compatible with the methodology.

**Table 8:** Yields for a Selection of Hydroamination Reactions Conducted by Mulks et al., Depicted in Scheme 51(332)

amide A	yield (%)
Piperidide	90
Pyrrolidide	75
N-Me-piperazide	>99
Morpholide	52

**Scheme 52:**
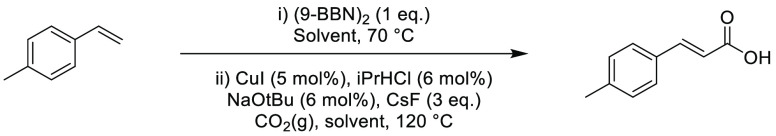
Telescoped Hydroboration–Carboxylation Conducted Using a Variety of Sustainable Solvents^208^

In 2020, a publication by Gevorgyan et al. investigated the utility of newly discovered biomass-sourced solvents in carboxylation reactions.^208^ In an overall transformation for the hydrocarboxylation of alkenes, via a boronate species from an initial hydroboration with 9-BBN, sustainable solvents including eucalyptol, dimethyl isosorbide (DMI), and GVL were screened, see Scheme 52. Given that the process occurs via a boronate species, the effectiveness of the secondary carboxylation of boronic acid pinacol esters, Bpins, was also explored. For the overall hydrocarboxylation of 4-methylstyrene, 2-MeTHF, eucalyptol, DMI, and GVL were all found to outperform dipolar aprotics of concern including THF and 1,4-dioxane, see Table 9. The hydroboration methodology was not restricted to just benzylic alkenes, that is, styrenes.^208^

**Table 9:**
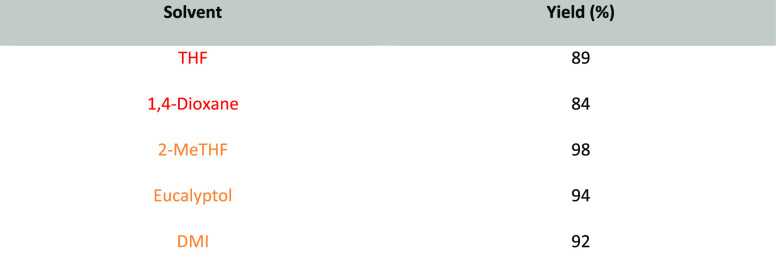
Yield Data for the Model Reaction, Depicted in Scheme 52(208)

**Scheme 53:**
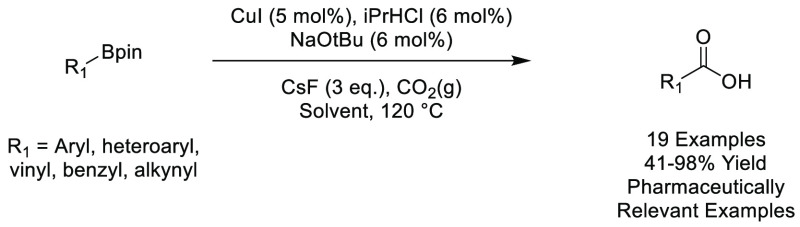
Bpin Carboxylation Transformation Solvent Screen^208^

For the conversion of phenyl Bpin to the corresponding phenyl carboxylic acid, of the general type depicted in Scheme 53, DMI was found to outperform THF and 1,4-dioxane, with 2-MeTHF and (+)-rose oxide also notably performing above 70% yield (Table 10). In addition to these two reactions, the solvents were comparatively screened for numerous other carboxylation processes including transition metal-catalyzed carboxylations (copper, zirconium, iron, and nickel).^208^ The Cu-catalyzed carboyxylation methodology was further applied toward the synthesis of active pharmaceutical ingredients Fenoprofen (60% yield) and Flurbiprofen (53% yield).

**Table 10:**
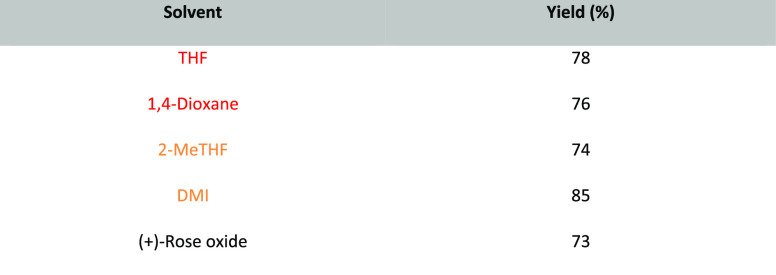
Yield Data for Model Transformation, Depicted in Scheme 53(208)

Pd-catalyzed alkoxy- and amino-carbonylation and aryl bromide/boronic acid carbonylation in greener more sustainable solvents has been recently demonstrated by Ismael et al.^333^ Sixteen potentially safer, more sustainable solvents were included during solvent screening investigations including dipolar aprotic alternatives such as DMI, GVL, ethylene carbonate and propylene carbonate, and ether 2-MeTHF. It was found that aminocarbonylation proceeded well in DMC while alkoxycarbonylation was successfully conducted in DMC and 2-MeTHF.^333^

**Scheme 54:**
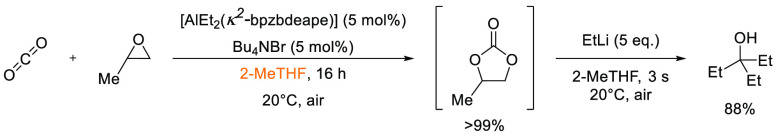
Al-Catalyzed Conversion of Epoxides and CO_2_ to Tertiary Alcohols via Cyclic Carbonates

Lastly, a very recent report by Elorriaga and colleagues disclosed a hybrid synthetic method in which an aluminum-catalyzed fixation of carbon dioxide in 2-MeTHF led to conversion of epoxides into tertiary alcohols.^334^ This process, catalyzed by AlEt_2_(κ^2^-bpzbdeape) (bpzbdeape = 1,1-bis(4-(diethylamino)phenyl)-2,2-bis(3,5-dimethyl-1H-pyrazol-1-yl)ethan-1-ol), results in insertion of CO_2_ into one of the epoxide C–O bonds and subsequent ring expansion to form 5-membered cyclic carbonates in a near-quantitative yield. These cyclic carbonates, derived from the structure of the starting epoxide, are then treated with 5 equiv of an alkyllithium reagent (e.g., EtLi), resulting in rapid conversion to the trialkyl alcohol. This methodology highlights an efficient method for the fixation of carbon dioxide to produce a range of highly substituted tertiary alcohols in one pot (Scheme 54).^334^

#### 3.1.9 Nucleophilic Aromatic Substitution (S_N_Ar)

**Scheme 55:**
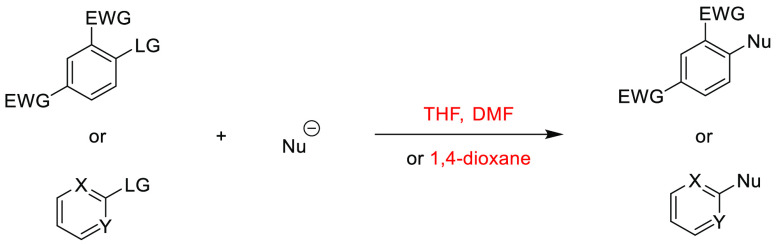
General Reaction Scheme for S_N_Ar-type Transformations

Nucleophilic aromatic substitution, or S_N_Ar, is one of the most commonly used classes of reaction for synthesis of aryl and heteroaryl ring systems, see Scheme 55.^260^ Because of the ease of reaction, including room temperature procedures, high-yielding transformations, and the ability to predict regioselectivity,^335^ the popularity of this reaction class has significantly increased in the previous 30 years.^260^ Although there are many examples using sustainable solvents in S_N_Ar reactions, incidences using solvents such as 1,4-dioxane and THF are also prevalent within the literature.^336,337^

**Scheme 56:**
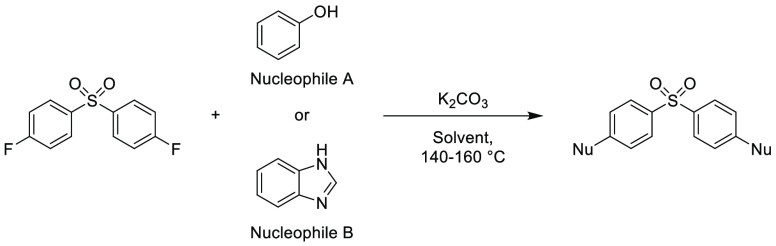
S_N_Ar Reaction Solvent Screening Conducted by Szekely et al.^104^

In the previous 5 years, a number of advances have been made toward the use of novel, sustainable solvents in nucleophilic aromatic substitution reactions (S_N_Ar). In 2019, Szekely and co-workers discussed the use of novel solvent PolarClean alongside other greener alternatives (GVL, Cyrene, and propylene carbonate) in a selection of reaction classes including S_N_Ar reactions.^104^ Using model reactions for the substitution of phenol or benzimidazole on substrate bis(4-fluorophenyl) sulfone, comparisons between the sustainable solvents and more traditional alternatives of concern (DMAc, toluene) were explored, see Scheme 56. Using phenol, the more favorable alternatives performed effectively compared to DMAc/toluene (86–92% compared to 70%) though were slightly less effective using benzimidazole (77–87% compared to 97%), see Table 11.

**Table 11:**
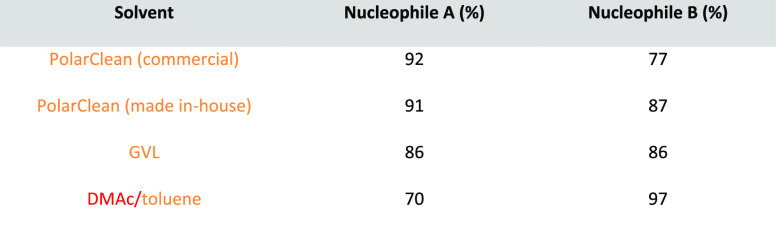
Yield Data for S_N_Ar Reactions Conducted by Szekely et al., Depicted in Scheme 56(104)

**Scheme 57:**
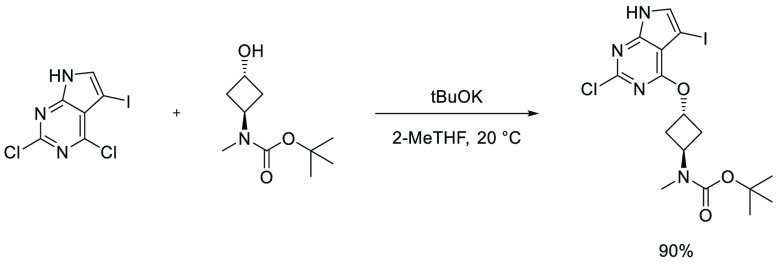
Switching of THF to 2-MeTHF in a Key S_N_Ar Step for Synthesis of a Medicinally Relevant EGFR Receptor Inhibitor^339^

Studying the use of Cyrene-derived solvents such as those falling into the Cygnet class, Clark and colleagues at the University of York explored the substitution of these solvents in nucleophilic aromatic fluorination reactions of pyridines.^66^ In the study, which also involved use in other reaction classes, kinetic investigations in the fluorination of 2-chloro-5-nitropyridine using a Cygnet 0.0 and a selection of dipolar aprotic solvents were carried out. Cygnet 0.0 resulted in higher reaction rates that MeCN and NMP, with comparable rates to DMF.^66^ A small selection of publications in the previous 5 years have opted for a straight solvent swap of the disfavored THF for the more sustainable alternative 2-MeTHF, mostly focusing on S_N_Ar reactions for medicinal chemistry applications. While investigating Buchwald-Hartwig cross-couplings, Buchwald and Smith discovered that in a small number of cases when coupling secondary amines with the model substrate, the reaction in 2-MeTHF proceeded effectively under noncatalyzed conditions.^338^ In the design of a greener synthetic route to an EGFR receptor inhibitor, McWilliams and co-workers from Pfizer substituted THF for 2-MeTHF in a key S_N_Ar step, yielding the desired product in 90% yield, see Scheme 57.^339^

A report by Parmentier et al. in 2016 demonstrated the utility of surfactant-based amphiphiles in the synthesis of a pharmaceutically relevant API.^340^ Looking to enhance the first generation route for synthesis, the group explored the use of TPGS-750-M in water over the 5-step synthesis, with the primary step involving an S_N_Ar reaction of an amine fragment and an (hetero)aryl electrophile (structures likely withdrawn from the publication due to intellectual property restraints). For this S_N_Ar step, a switch from organic solvents to a surfactant–water system resulted in a slightly diminished yield of 75% compared to 87%, but for the overall 5-step route, the process mass intensity (PMI) of the process was decreased by 32%.^340^

**Scheme 58:**
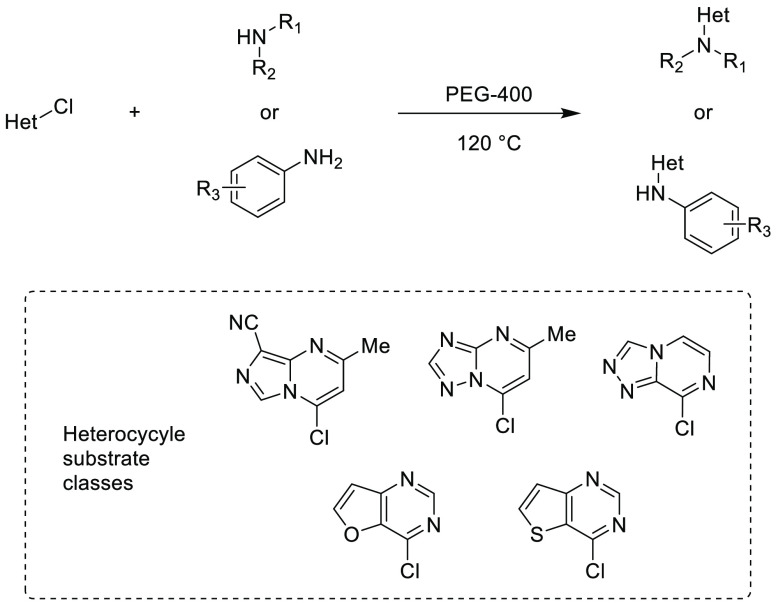
S_N_Ar Reactions Conducted in PEG-400^341^

More recently, Berteina-Raboin and co-workers explored the use of the widely available PEG-400 (polyethylene glycol) as a solvent in nucleophilic aromatic substitution reactions, see Scheme 58.^341^ The reaction was shown to perform effectively, using a range of aliphatic and aromatic amines, on medicinally relevant substrate classes including imidazo[1,5-*a*]pyrimidines, [1,2,4]triazolo[1,5-*a*]pyrimidines, and [1,2,4]triazolo[4,3-*a*]pyrazines. For the wide ranging substrate scope, yields are reported in the range of 70–99%.^341^

Various PEG variants have also been successfully applied in many metal-mediated reactions including oxidation, reduction, cross-coupling, and polymerization. This subject has been well reviewed by Colacino et al.^342^

Often, organic solvents can be replaced entirely from S_N_Ar type chemistry. In 2013, Walsh et al. demonstrated that several S_N_Ar reactions comprising heteroaryl chlorides such as pyrimidine, pyrazine, and quinazoline could be conducted in water in the presence of KF. Surprisingly, several transformations that were previously reported in the literature as requiring transition metal catalysis were able to be conducted free from Pd.^343^

#### 3.1.10 Organometallic Reactions

**Figure 31:**
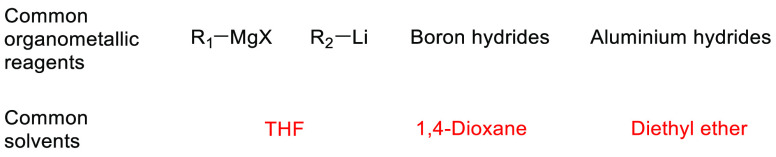
Organometallic reagents and their commonly employed ethereal solvents.

Organometallic-based reactions, such as those involving the use of alkyl lithium reagents, Grignard reagents, and metal hydride reductions, are commonly carried out in ethereal solvents and hydrocarbons due to the need for stability under basic conditions.^204,344^ The most commonly used solvents within this class include diethyl ether, THF, and 1,4-dioxane, all of which are solvents of concern, see Figure 31.^8^ In recent years, as a solution to the ongoing issues surrounding these solvents, 2-MeTHF^158,160^ and CPME^345^ have both been proposed as sustainable alternative solvents in a range of organometallic reactions, key examples of which will be discussed in this section. A number of commonly employed organometallic reagents are also now available as commercial preparations in alternative solvents such as 2-MeTHF.^346^

##### 3.1.10.1 Organometallic Reactions Employing 2-MeTHF

**Scheme 59:**
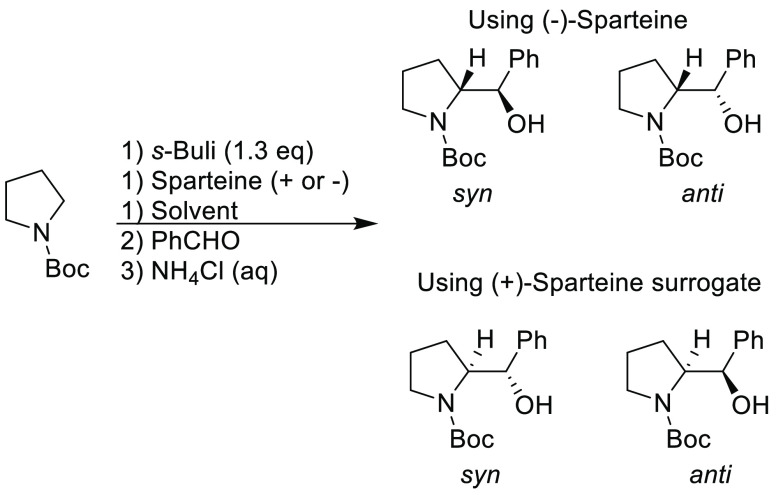
Asymmetric Lithiation Reactions Conducted Using Chiral Amines in a Variety of Ethereal Solvents^347^

2-MeTHF has been successfully utilized in a multitude of organometallic reactions, often acting as a “drop in” replacement for THF or 1,4-dioxane. In 2010, Carbone et al. demonstrated that 2-MeTHF could be used as solvent in asymmetric deprotonation reactions where a chiral amine anion base is prepared *in situ*. Remarkably, the chiral nature of the amine base is critical for enantioselectivity in asymmetric lithiation reactions when THF or 2-MeTHF are used as solvent; anion bases derived from (−)-sparteine proceeded with low enantioselectivity, whereas when a (+)-sparteine surrogate was employed, excellent selectivity was observed.^347^ When MTBE was employed as solvent, excellent enantioselectivity was observed for both sparteine bases, see Table 12 and Scheme 59. The selectivity is explained by the authors through a series of experiments investigating the solution phase dimerization and organization of the lithium base solvent complexes. The (+)-sparteine surrogate used is (+)-(1*R*,2*S*,9*S*)-11-methyl-7,11-diazatricyclo[7.3.1.0^2,7^tridecane].

**Table 12:**
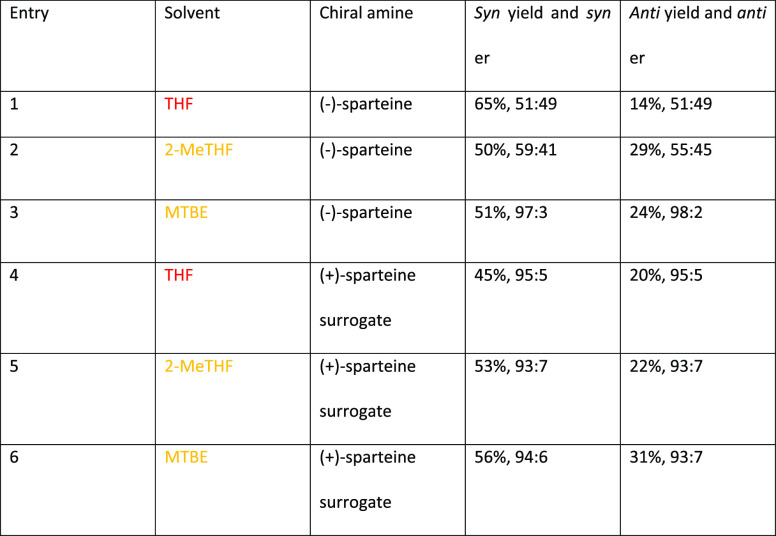
Yield and Enantiomeric Ratio Data for Asymmetric Lithiation Reactions Conducted Using Chiral Amines in a Variety of Ethereal Solvents^347^

**Scheme 60:**
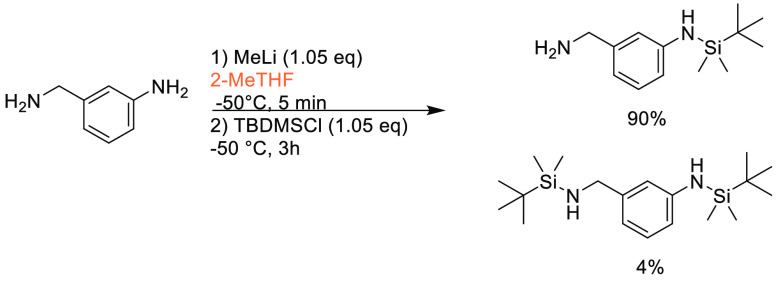
Site-Selective *N*-TBS Protection Using 2-MeTHF as Solvent^348^ Note: −50 °C used here instead of 0 °C.

Mild, chemoselective *N*-TBS protection of anilines has been reported by Pace et al. using 2-MeTHF as a direct replacement for THF. Under their optimized reaction conditions, TBS protection of aniline was conducted at 0 °C in just 30 min using MeLi and TBDMSCl in excellent yields (88–98%, 15 examples). Excellent chemoselectivity was also observed when iodo and bromo anilines were utilized as substrates; no lithium-halogen exchange was observed when using 2-MeTHF as solvent but exchange did occur when using diethyl ether. Finally, site selective TBS protection of aniline nitrogen over alkylamino nitrogen atoms was demonstrated, see Scheme 60. When using THF or diethyl ether, a 1:1 mixture was observed. This remarkable selectivity has been proposed by the authors as being due to a reduction in the amount of highly polar amine dianion formed in 2-MeTHF due to the solvent’s polarity.^348^ Additionally, the authors undertook an examination of deprotection conditions showing that it was possible to deprotect the TBS protected anilines in near quantitative yield in 2 h by simply stirring the compounds in a suspension of silica gel in an ethanol:water mixture (1:5).

**Scheme 61:**
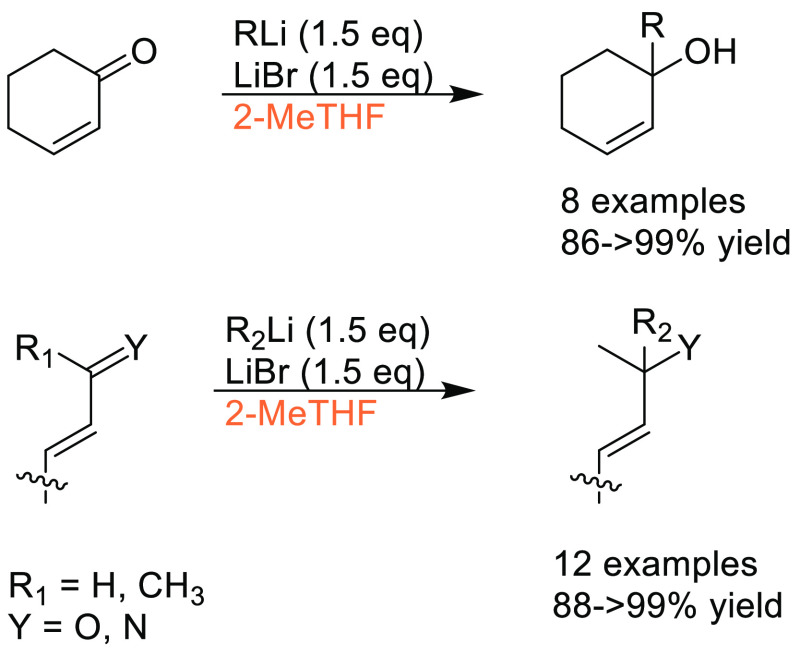
LiBr-Mediated Selective 1,2-Addition of Organolithium Reagents to α,β-Unsaturated Imines, Ketones, and Aldehydes^349^

The utility of 2-MeTHF in LiBr mediated selective 1,2-additions of organolithium reagents to α,β-unsaturated imines, ketones, and aldehydes to give a variety of allylic alcohols and amines has been demonstrated.^349^ Notably, no 1,4-addition products were detected by ^1^H NMR, and the reactions were conducted at 0 °C making this an attractively simple method. Analogous reactions conducted in THF took longer and gave poorer yields when compared to 2-MeTHF (e.g., 6 h 79% yield vs 2 h quantitative yield for the 1,2 addition of MeLi to cyclohexenone), see Scheme 61.

**Scheme 62:**
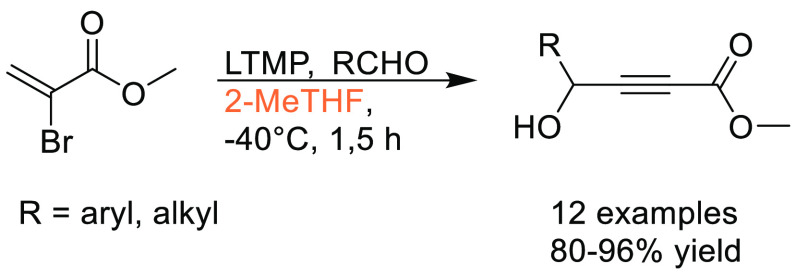
Synthesis of γ-Hydroxy-α,β-acetylenic Esters Using LTMP in 2-MeTHF^350^

Other lithium base mediated reactions that have been successfully conducted in 2-MeTHF include preparations of γ-hydroxy-α,β-acetylenic esters using the base lithium tetramethylpiperidide (LTMP).^350^ Once more, Pace et al. have demonstrated the superior utility of 2-MeTHF through their exhaustive screening of hydrocarbon and ethereal solvents in the reaction of methyl-2-bromoacrylate with benzaldehyde in the presence of a strong alkyllithium base, see Scheme 62. The 2-MeTHF gave a 94% yield of the desired alcohol in just 1.5 h at −40 °C compared to THF, which gave just 67% under the same conditions.

**Scheme 63:**
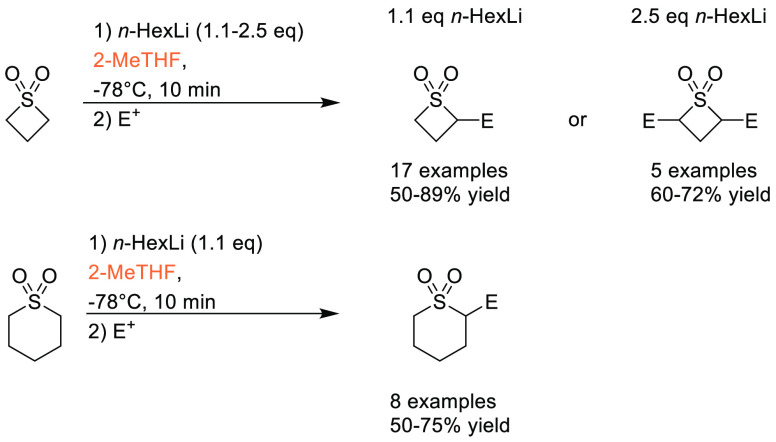
Functionalization of 4- and 6-Membered Cyclic Sulfones via a Lithiation–Electrophile Trapping^351^

Efficient access decorated four- and six-membered sulfur containing heterocycles (i.e., thietanes and thiopyrans) have also been achieved through a lithiation electrophile trapping strategy using 2-MeTHF as solvent, see Scheme 63. The 2-MeTHF was shown to improve regioselectivity considerably over THF.^351^ A wide range of benzylic and aliphatic electrophiles were utilized, and varying degrees of electrophile dependent stereocontrol were observed.

Other areas of organometallic chemistry in which 2-MeTHF has seen utility include zirconium chemistry such as that employing Schwartz’s reagent ((C_5_H_5_)_2_ZrHCl). Schwartz’s reagent in the presence of 2-MeTHF has been used to reduce isocyanates to formamides under mild conditions with excellent chemoselectivity and stereoretention of chiral substrates observed (room temperature, 1 h), see Scheme 65.^352^ THF gave slightly poorer yields of about −5% during solvent screening reaction development. Similarly, Schwartz’s reagent can be utilized to reduce isothiocyanates to thioformamides.^351^ Electron withdrawing groups attached to the aromatic isocyanates were also well tolerated including reactive functional groups such as azo and azide motifs.^353^ The use of 2-MeTHF and the use of an *in situ* generated Schwartz reagent as the hydride source were crucial for obtaining high chemocontrol.^354^

**Scheme 64:**
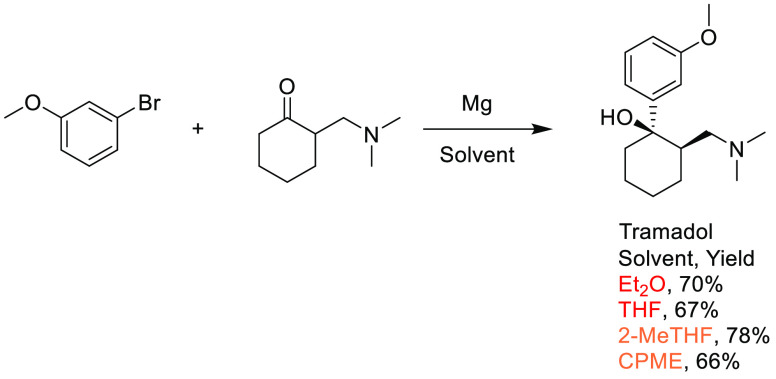
Grignard Synthesis of API Tramadol Using a Variety of Alternative Solvents

**Scheme 65:**
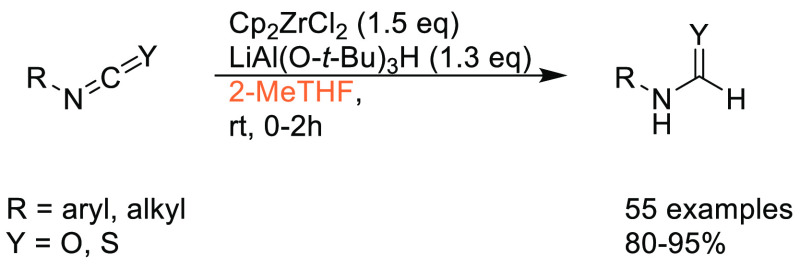
Reduction of Isocyanates and Isothiocyanates to Formamide Using Schwartz’s Reagent in 2-MeTHF^352,353^ Schwartz’s reagent is prepared *in situ* from Cp_2_ZrCl_2_ and LiAl(O-t-Bu)_3_H, that is, Snieckus protocol.

Subtle differences in the solubilizing and stabilizing effect of 2-MeTHF have been effectively leveraged by Lelo et al., who have successfully employed this solvent to promote α-elimination of lithium halide salts to carbenoids, that is, Kirmse’s α-elimination. Taking advantage of the unique properties of 2-MeTHF, LiX salts have been degraded to carbenoids and reacted *in situ* with epoxides to give β-halohydrins. Employing THF or diethyl ether under the same conditions helps to stabilize the lithium salts, and the transformations were poorly accommodated by these more stabilizing solvents.^355^ Other uses of ICH_2_X include the NaBH_4_-mediated reduction of diselenides in the presence of ICH_2_F to give α-fluoromethyl selenoethers.^356^

**Scheme 66:**
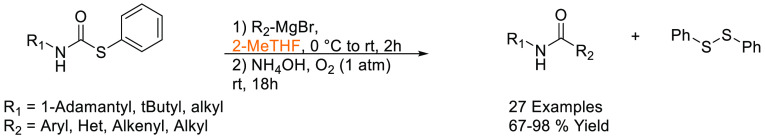
Secondary Amide Formation from Thiocarbamates Using Grignard Reagents in 2-MeTHF^357^

**Scheme 67:**
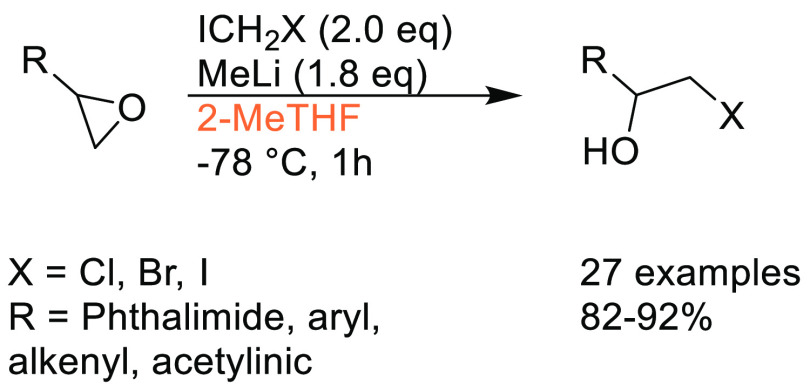
Epoxide Ring-Opening Giving β-Halohydrins^355^

Synthesis of secondary amides from phenyl thiocarbamates and commercially available Grignard reagents has been demonstrated by Mampuys et al. utilizing 2-MeTHF as solvent, see Scheme 66.^357^ Good to excellent yields were observed across a wide variety of substrates including sterically hindered and electron-deficient compounds (Scheme 67).

**Scheme 68:**
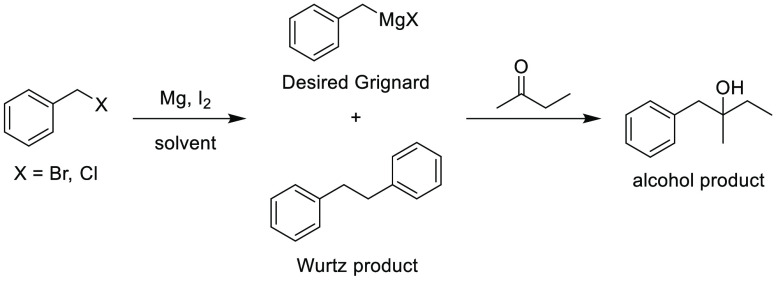
Successful Replacement of THF or Et_2_O with 2-MeTHF in Grignard Reactions^177^

The Grignard reaction manifold represents a classical and key methodology for C–C bond formation. Grignard reactions, at their simplest, involve the addition of an organomagnesium halide to a carbonyl containing molecule, forming a new C–C bond and an alcohol. The reaction was discovered in 1900 by Victor Grignard, for which he was awarded the Nobel Prize in Chemistry in 1912.^358^ The reaction today is still widely employed and has even been conducted in the large scale manufacturing of active pharmaceutical ingredients and their intermediates.^177^ Grignard reactions have classically employed solvents of concern such as THF or diethyl ether, thus prompting the research group of Zhang et al. to undertake a systematic study evaluating the performance of a variety of alternative and more sustainable solvents in Grignard-type reactions.^177^ It was demonstrated that 2-MeTHF performed just as well or out-performed diethyl ether or THF in a wide variety of Grignard reactions including benzyl, aryl, and heteroaromatic Grignard reagents. CPME was also shown to be an appropriate solvent when DIBAL-H instead of I_2_ was used as an initiator. A solvent comparison study for the formation of API tramadol was also conducted, see Scheme 64, with 2-MeTHF outperforming THF by +11% yield. After a performance evaluation and screening of solvents for a range of Grignard reactions, Zhang et al. now recommend the use of 2-MeTHF as alternative solvent to Et_2_O and THF for the preparation of most Grignard reagents and their subsequent reactions, see Scheme 68. They noted that the use of 2-MeTHF was equal if not superior in suppressing the Wurtz coupling byproduct from benzyl Grignard reactions, see Table 13.^177^

**Table 13:**
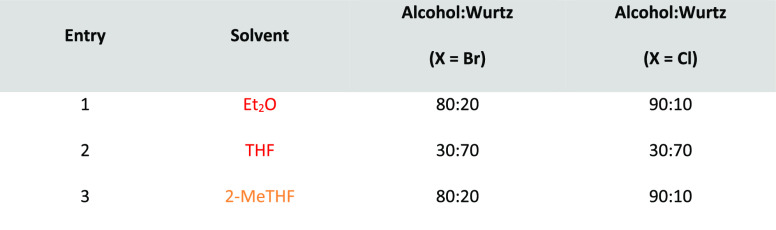
Yield and Selectivity Data for Grignard Reaction, Depicted in Scheme 68(177)

**Scheme 69:**
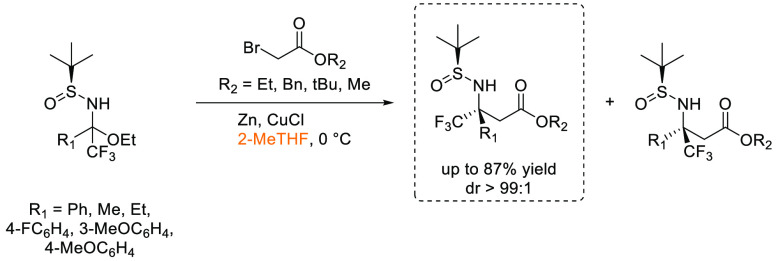
Reformatsky Reactions Conducted in 2-MeTHF^359^

The use of Reformatsky reagents in the large-scale synthesis of various enantiopure *N*-tert-butanesulfinyl trifluoromethyl β-amino esters from bench-stable analogues of aliphatic and aromatic trifluoromethyl *N*-tert-butanesulfinyl ketoimines was reported by Grellepois, see Scheme 69. During optimization studies, it was found that 2-MeTHF was the best solvent.^359^

**Scheme 70:**
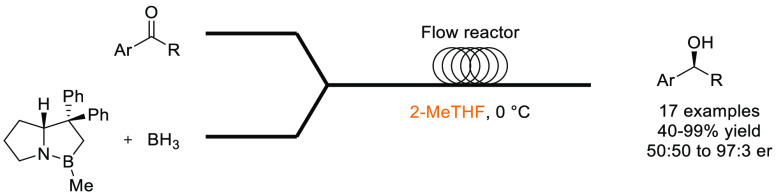
CBS Oxazaborolidine-Mediated Reduction of Prochiral Ketones, in Continuous Flow, Using 2-MeTHF as Solvent^360^

The synthesis of enantioenriched alcohols via the Corey-Baskshi-Shibata (CBS) oxazaborolidine-mediated reduction of prochiral ketones was carried out by Luisi and co-workers using a combination of flow technology and 2-MeTHF, see Scheme 70. The optimized conditions gave the desired asymmetric products in yields of up to 99% and 91:9 enantiomeric ratio (er), with a reaction duration 10 min.^360^

##### 3.1.10.2 Organometallic Reactions Employing CPME

CPME, as discussed preciously in section 2, has been available in commercially useful quantities since 2005^165^ and has been utilized in a wide variety of reaction classes including organometallic mediated transformations. A selection of examples highlighting some of the breadth and scope of CPME employment in organometallic reactions will be discussed in this section.

**Scheme 71:**
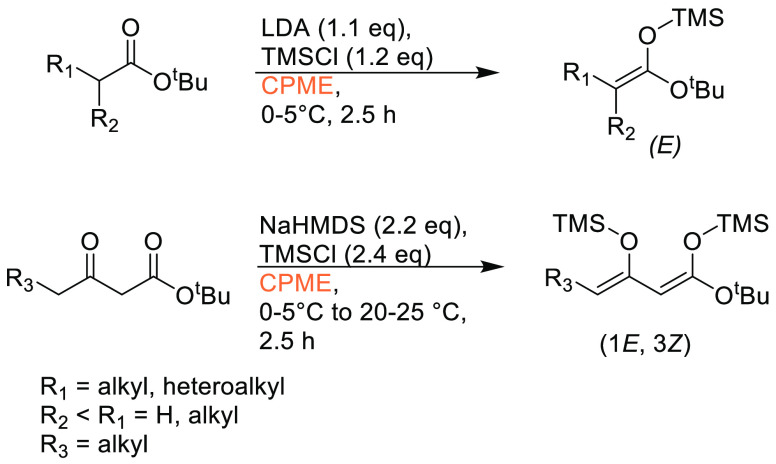
CPME Utilized as Solvent in Synthesis of Ketene Trimethylsilyl Acetals

In 2007, Okabayashi et al. developed a facile method for the regio- and stereoselective preparation of ketene trimethylsilyl acetals (KSAs) from *t*-butyl esters or ketoesters using strong bases such as LDA and NaHMDS, in conjunction with TMSCl, and CPME as solvent. The reactions were conducted under mild conditions, 0–5 °C, 2.5 h and produced both (*E*)-KSAs and 1,3-bis(TMS)-KSAs, see Scheme 71.^361^ α-Oxygen and α -nitrogen-substituted *t*-butyl esters were also shown to be compatible with this methodology. During solvent screening for reaction condition optimization, it was shown that other ethereal solvents such as diethyl ether, THF, 1,4-dioxane, and dimethoxyethane led to poor regioselectivity and *C*-silylation, whereas CPME by comparison outperformed all of these solvents.

**Scheme 72:**
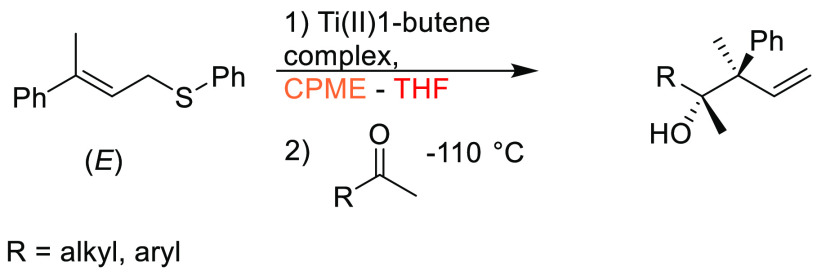
Ti-Mediated Ketone Allylation Using CPME as Co-solvent^362^

CPME has also seen use as a cosolvent in conjunction with THF, see Scheme 72. Takeda et al. demonstrated that CPME was critical for a titanocene-mediated ketone allylation strategy that gave access to alkyl systems containing adjacent stereocenters in high yield and high diastereoselectivity.^362^ CPME has also been used as a co-solvent (in conjunction with toluene) by researchers at Novartis who employed the solvent as a stabilizer for a Grignard reagent during a reaction sequence.^363^

**Scheme 73:**
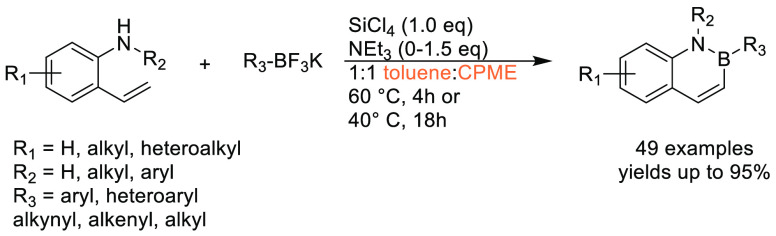
Azaborine Synthesis in 1:1 Mixture of Toluene:CPME^364^

The stabilizing effect of CPME was once again exploited by Molander et al. during their reaction optimization for the synthesis of azaborines. A 1:1 mixture of CPME:toluene was discovered to be optimum.^364^ Azaborines represent an important and emerging class of potential isosteric replacements for traditional carbon containing heterocycles, and the methodology developed, Scheme 73, has allowed for facile synthesis using, at least in part, a more contemporary solvent.

**Scheme 74:**
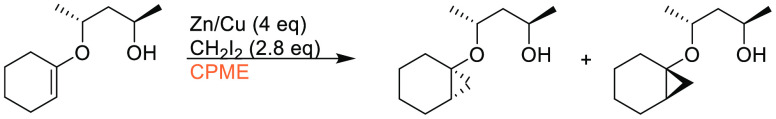
Simmons-Smith Cyclopropanation Conducted Using CPME as Solvent

The Simmons-Smith cyclopropanation is a classical method for the synthesis of cyclopropanes from alkenes via insertion of a zinc carbene, see Scheme 74. THF or diethyl ether is most often employed as solvent in this reaction manifold, and in 2013, Fujii et al. conducted solvent screening to determine whether other alternative ethers could be used. It was demonstrated that reactions conducted using CPME went to completion up to ten-times faster than those in diethyl ether with no loss in product yield or selectivity. Yield improvements in some cases were also noted.^365^

**Scheme 75:**
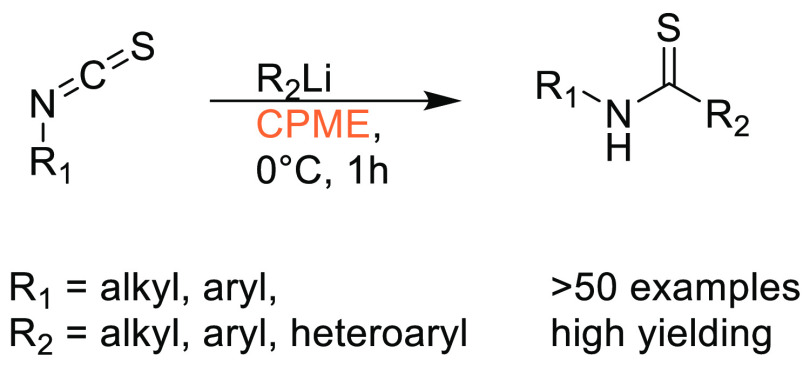
Thioamide Formation Using Isothiocyanates and Lithium Reagents in CPME Solvent System^366^

In 2015, Pace et al. demonstrated that CPME was the optimum solvent for their synthesis of thioamides from the corresponding isothiocyanate and lithium reagent, see Scheme 75. Solvent screening showed CPME to be superior to traditional ethers such as THF and diethyl ether and even outperformed 2-MeTHF in terms of yield. The methodology reported provides robust and efficient access to secondary thioamides from commercially available isothiocyanates and includes examples where chiral isothiocyanates or organolithium reagents have been used to form enantiopure products with excellent selectivity.

**Scheme 76:**
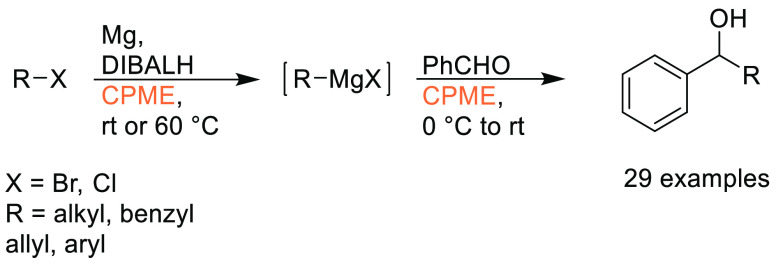
Grignard Reactions Conducted Using CPME as Solvent

In 2016, a thorough investigation into the use of CPME in Grignard reactions was conducted by Kobayashi et al., see Scheme 76.^345^ It was shown that CPME can be used in place of THF to great effect and that DIBALH was the most effective method of activating the Mg when using CPME as solvent. Efficient solvent recycling of CPME was also demonstrated with no loss of yield observed. Promisingly, CPME was shown to be stable to Grignard reagents during long-term storage with no solvent degradation observed. Furthermore, Grignard reagent stability in CPME was often in the order of many months; thus, CPME could be used in commercial preparations of Grignard reagents.^345^

#### 3.1.11 Urea Synthesis

**Scheme 77:**
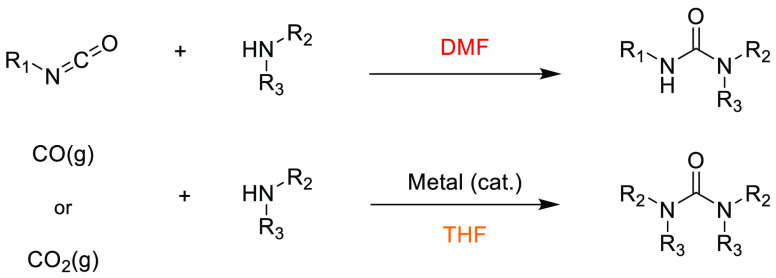
General Reaction Scheme for Various Types of Urea Synthesis

Ureas are used in agrochemicals, and in the pharmaceutical industry where they are found in areas such as antibiotics, antimalarial compounds, antimicrobials, and many others. They have also been utilized as catalysts, ligands, and solvents, see Scheme 77.

**Scheme 78:**
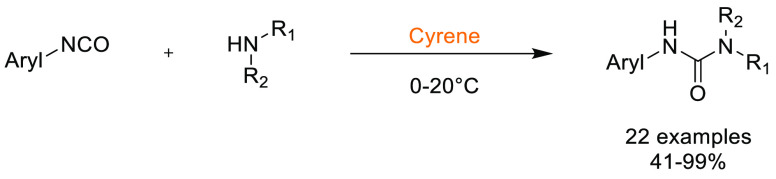
Urea Synthesis Utilizing Bio-Based Solvent Cyrene^121^

A greener, mild, and efficient approach for the synthesis of ureas, from isocyanates and secondary amines, was developed by Camp and co-workers using the bioalternative solvent Cyrene, see Scheme 78.^88^ This method removes the use of toxic solvents such as DMF, which has been used in the traditional synthesis of ureas. A simple workup procedure for the removal of Cyrene was also established by the addition of water, which led to precipitation of the desired urea. The substrate scope of both the amine and isocyanate was investigated.

#### 3.1.12 Steglich-type Esterification

**Scheme 79:**
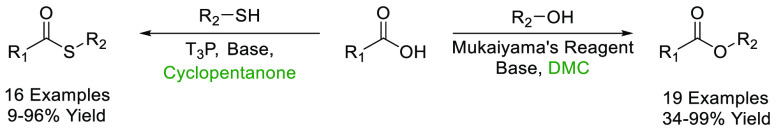
Ester and Thioester Formation Conducted in Replacement Solvents Such as Cyclopentanone and DMC^367−369^

Steglich esterification remains a commonly employed technique for the mild synthesis of esters from carboxylic acids and alcohols or thiols.^184,367^ This methodology employs coupling reagents often encountered in amide bond formation such as carbodiimides and relies heavily on either DCM or DMF as solvents of choice.^8,367^ Solvent-reagent selection guides for both ester and thioester synthesis have been developed by Jordan et al. and effectively demonstrate the effective replacement of dipolar aprotic solvents with safer, more sustainable alternatives, see Scheme 79.

For the formation of thioesters from acids and thiols, it was found that coupling reagent T3P in conjunction with solvent cyclopentanone gave good to excellent yields across a variety of building-block-like molecules, often with rapid reaction times of just 1 h, see Scheme 79 (left). 2-MeTHF, EtOAc, DMC, and CPME were also shown to be viable alternatives, though less general in their applicability/compatibility to the substrates screened.^184^

A solvent-reagent guide for ester formation from alcohols and acids has also been recently developed. In this study, a high-throughput approach allowed for the simultaneous identification of alternative solvent DMC in conjunction with safer coupling reagent 2-chloro-1-methylpyridinium iodide (Mukaiyama’s reagent). Further optimized conditions were identified from the initial screen and then successfully applied in the synthesis of a selection of building-block-like molecules, see Scheme 79 (right). In both studies, less desirable polar aprotic solvents were successfully replaced with greener, safer, more sustainable alternatives.

Steglich esterification in water has also been demonstrated by Fattahi et al. using DIC as coupling reagent, though the methodology is limited to phenolic alcohols due to their lower p*K*_a_ when compared to aliphatic alcohols in water.^265^

#### 3.1.13 Solid Phase Peptide Synthesis (SPPS)

**Scheme 80:**
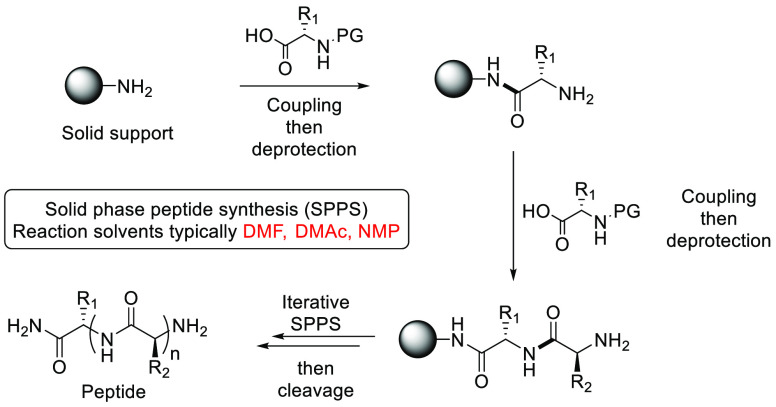
General Overview of SPPS Operation

A range of peptide-based compounds are widely used globally, including clinically used hormones such as oxytocin, and peptide therapeutics. From 2015–2019, 208 new drugs were approved by the FDA, 15 of which were peptides or peptide-containing molecules (7%).^370^ The increasing proportion of therapeutics falling into the peptides category has been accompanied by a need for more sustainable methods for the solid phase synthesis of peptides (SPPS), see Scheme 80. Commonly used solvents for SPPS fall within the reprotoxic dipolar aprotics category, being DMF, DMAc, and NMP.^371^ The challenges in improving the sustainability in peptide synthesis and purification are well reviewed by Isidro-Llobet et al.^372^

Recently, a number of publications have aimed to move away from reprotoxic dipolar aprotic solvents in solid phase peptide synthesis, switching to more sustainable alternatives. The work of Lawrenson et al. has successfully demonstrated the use of propylene carbonate as a safer, more sustainable alternative to polar aprotic solvents such as DMF or NMP. In their publication, both SPPS and solution phase methodology were successfully demonstrated using propylene carbonate as solvent with no significant impact on racemization of the amino acid (AA) observed. Synthesis of nonapeptide “Bradykinin” was also demonstrated.

NBP was identified by Novartis in 2018 during a systematic examination of 34 alternative solvents shortlisted as potential DMF replacements in SPPS.^222^ A number of parameters were examined including solvent ability to swell resin, solubility of SPPS reagents, and reaction performance including Fmoc deprotection steps. NBP was successfully demonstrated as a viable drop-in replacement for DMF; NBP was successfully used as the solvent during the SPPS synthesis of an octapeptide with comparable yield and purity to that of DMF (93% yield NBP vs >99% DMF, 80% purity NBP vs 86% DMF). One major point of difference was highlighted: NBP is more viscous than DMF (4.0 cP vs 0.8 cP) and makes for slower fluid transfer steps during smaller scale SPPS operations.^222^

Two reports were published in 2020 in which the solvent NBP was used in place of traditional solvents. Kumar et al. have successfully demonstrated the use of NBP as a replacement for DMF and NMP SPPS operations using both *in situ* activation and preactivation strategies. Less racemization was observed for reactions conducted in NBP, and a reduction in the generation of side-products when forcing conditions were utilized was also observed. Thus, NBP has been demonstrated as a superior alternative to both DMF and NMP in this study.^373^

De la Torre et al. report the successful use of NBP in an SPPS sequence employing problematic amino acid residue Fmoc-Arg (Pbf)–OH; pbf = (2,2,4,6,7-pentamethyldihydrobenzofuran-5-sulfonyl group). The amino acid residue is particularly troublesome due to its propensity to react intramolecularly leading to δ-lactam side product. A systematic analysis of reaction conditions allowed for the identification of optimum conditions, and NBP was shown to be an excellent alternative solvent to DMF, with levels of amino acid incorporation into the peptide backbone approaching 100%. Purity of the crude peptides was also similar.^374^

From a safety perspective, it has been reported by Erny et al. that the levels of HCN produced in DIC/Oxyma mediate amide bond forming reactions can be significantly reduced when a mixture of NBP:EtOAc 1:4 is employed.^375^ In conjunction with dimethyl trisulfide (DMTS) as a HCN scrubbing additive, the authors suggest that with further developments, the implementation of this methodology combining greener solvents and HCN-free synthesis could be applied to the industrial manufacturing of peptides.

Other than NBP, GVL has also been proposed as a potential alternative solvent in SPPS. The group of Albericio and co-workers illustrated that GVL could be utilized in polystyrene (PS)-based SPPS. GVL showed excellent coupling efficiencies in both the synthesis of a pentapeptide (Aib-enkephalin) and decapeptide (Aib-ACP), with no major side products or ring opening products detected.^376^ A follow-up to this work was published in 2019 demonstrating the effectiveness of GVL in Wang resin SPPS; DCM was effectively replaced as an alternative solvent for the initial anchoring to the Wang resin. Subsequent elongation of the peptide was also successfully conducted in GVL with racemization and dipeptide formation found to be within acceptable levels.^377^ Both publications also note the compatibility of Fmoc residues in GVL. Most recently, Leko et al. have demonstrated that a combination of EtOAc/CH_3_CN can be used in chlorotrityl chloride and 4-methylbenzhydryl bromide resin loading operations with loading levels comparable to that when DCM, THF, 2-MeTHF, or DMF was utilized.^378^

#### 3.1.14 Biocatalysis

In recent years, there has been a significant expansion in the field of biocatalysis, commonly using water as a solvent or cosolvent. However, there are a number of problems associated with the use of water as a solvent in biocatalysis, even though it is readily available, is nonhazardous, and does not have the flammability or explosion risks associated with organic solvents.^379^ Many synthetically useful reagents, those that are of interest in biocatalytic transformations, are poorly water-soluble, which in turn results in a requirement of extremely dilute reaction mixtures, leading to increased solvent use. There has been a recent drive for discovery of enzymatic reactions compatible with sustainable organic solvents, most notably 2-MeTHF^380^ and CPME.^168^

**Scheme 81:**
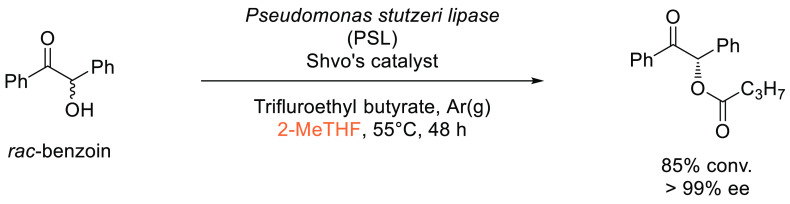
Bio-Catalyzed Kinetic Resolutions of *rac*-Benzoin Using *Pseudodomonas stutzeri* Lipase (PSL) and Shvo’s Catalyst in Bio-Based Solvent 2-MeTHF

The most expansive class of enzymatic reaction using 2-MeTHF is that of hydrolases, mostly in stereoselective acylations for kinetic resolutions of racemates.^381−383^ In 2017, Secundo and co-workers reported the use of 2-MeTHF in the esterification of a range of natural products (menthol, *rac*-sulcatol and *rac*-α-cyclogeraniol) using a range of commercial lipases.^384^ 2-MeTHF, as well as CPME, performed well compared to traditional solvents such as toluene and MTBE, with high enantioselectivity. A similar enzymatic acylation of racemic benzoin in 2-MeTHF, reported by Alcántara and colleagues in 2011, using *Pseudomonas stutzeri* lipase (PSL) and Shvo’s catalyst, led to kinetic resolution over 48 h, in a 85% yield and 99% e.e. (Scheme 81).^385^ Numerous additional examples are also found within the literature using 2-MeTHF as an alternative bio-based solvent, targeting selective acylations of glycosides.^386−390^

Away from kinetic resolutions, *Pseudomonas stutzeri* lipase (PSL) has additionally be used as an effective catalyst in amination reactions of esters, reported by Wong et al. in 2011.^391^ Shown to form a wide range of alkyl and aryl amides, 2-MeTHF was reportedly selected due to a high solubilizing ability of the organic starting materials, with MTBE also shown to perform effectively in the transformation.

**Scheme 82:**
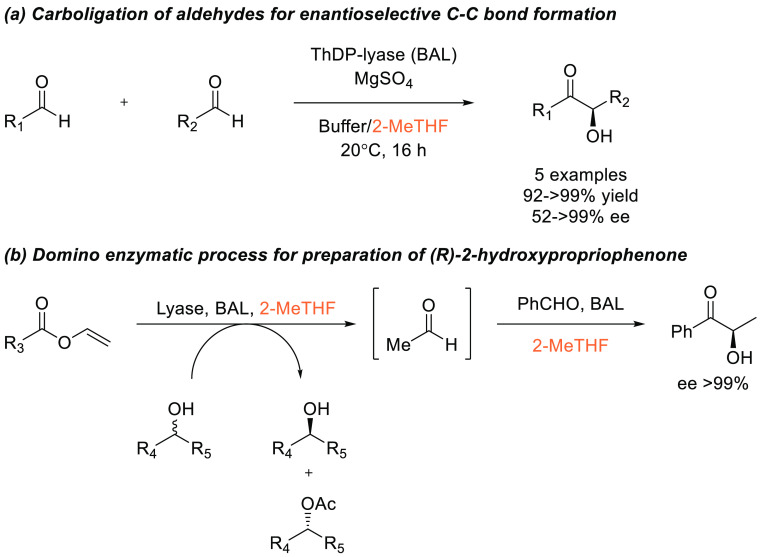
Enantioselective C–C Bond Formation Using Benzaldehyde Ligase (a) Carboligation of aldehydes to produce chiral α-hydroxyketones. (b) Domino enzymatic process for the preparation of 2-HPP, with *in situ* generation of acetaldehyde via kinetic resolution.

A selection of papers using 2-MeTHF as a bio-based solvent, for accessing chiral α-hydroxyketones through either carboligation^392,393^ or domino enzymatic reactions,^394^ have also been reported. The first of which, by Domínquez de María in 2010, involved use of 2-MeTHF as a (co)solvent alternative to DMSO or MTBE for enantioselective C–C bond formation between aldehydes. Catalyzed by a thiamine diphosphate-dependent lyase (ThDP-Lyase), an efficient enantioselective carboligation of aldehydes to furnish chiral α-hydroxyketones was described, providing products in >90% yield and generally >95% e.e. (Scheme 82a). A similar report by Rother and co-workers in 2012 also used ThDP-Lyase for the *umpolung* carboligation of acetaldehyde and benzaldehyde, examining the relative product distribution of the eight possible enantiomeric products.^393^

This was further followed up in 2012 by a domino enzymatic cascade using an aldehyde and butyrate or laurate substrate, in the presence of *Candida antarctica* lipase B (CAL-B) and benzaldehyde lyase (BAL).^394^ An initial conversion of the vinyl ester into acetaldehyde, followed by a subsequent C–C bond formation with benzaldehyde, catalyzed by BAL, furnished (*R*)-2-hydroxypropriophenone (2-HPP) in quantitative conversion and >99% e.e. (Scheme 82b).

**Scheme 83:**
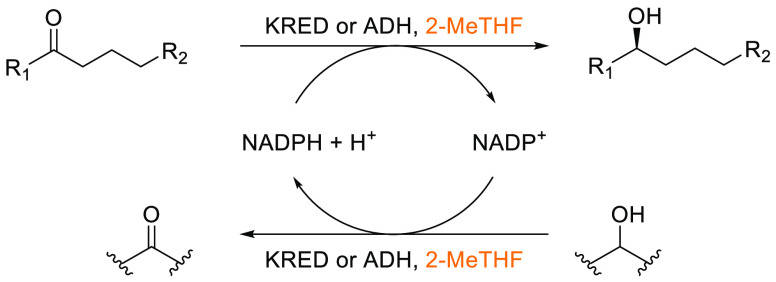
General Scheme for Enzymatic Reductions of Ketones Using Ketoreductases (KRED) or Alcohol Dehydrogenases (ADH) in 2-MeTHF^396,397^

Although the majority of biocatalytic transformations carried out in organic solvents such as 2-MeTHF fall under the hydrolases class due to their high robustness, a small number of other enzymes are also compatible. In 2014, metagenome screening by Kurokawa and colleagues identified a dehydrogenase enzyme, homologous Leifsoniaadh alcohol dehydrogenase (HLADH), which was able to effectively reduce a selection of ketones, with high tolerance to 2-MeTHF, in excellent (generally >99%) enantiomeric excess.^395^ Similar ketone reductions have been reported, for example, using YOL151W reductase^396^ and KREDs (Scheme 83).^397^

**Scheme 84:**
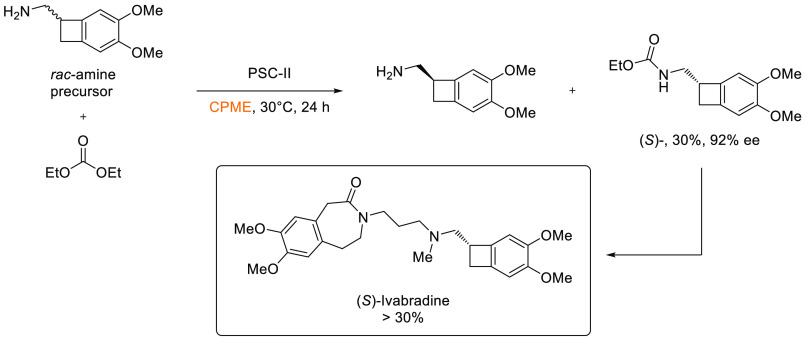
Use of Lipase PSC-II in CPME for Kinetic Resolution of Racemic Alcohol for Use in Synthesis of (*S*)-Ivanbradine, a Drug for Treatment of Stable Angina Pectoris

Other than 2-MeTHF, a solvent (or cosolvent) that has demonstrated potential within biocatalytic transformations is cyclopentyl methyl ether (CPME). CPME, similar to 2-MeTHF, has proven effective in kinetic resolution, such as the one reported by González-Sabín and co-workers in 2017.^398^ As part of a key step in the synthesis of Ivabradine, a drug used for the treatment of stable angina pectoris, alkoxycarbonylation kinetic resolution of an amine precursor was carried out using the lipase PSC-II and diethylcarbonate, yielding the desired (*S*)-enantiomer in 30% yield, 92% e.e. (Scheme 84).

**Scheme 85:**
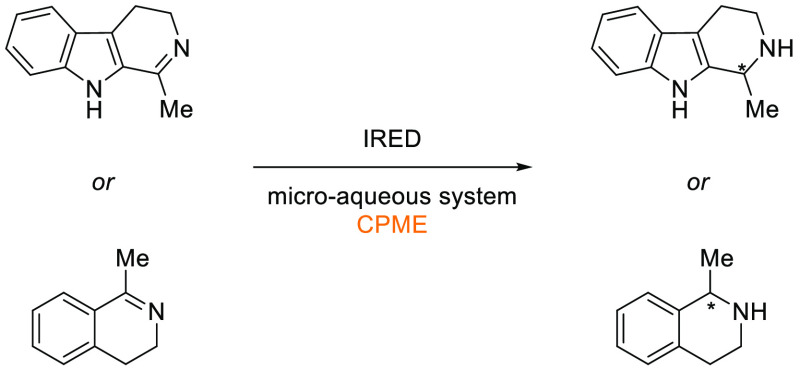
Use of Imine Reductases (IRED) to Reduce Iminic-type Functional Groups in Heterocyclic Rings to Chiral Amines, Using CPME as Reaction Solvent^399^

The utility of CPME as a solvent in the reduction of imine-type functionalities, using IREDs (imine reductases), was reported by Rother and Maugeri in 2016.^399^ Reduction of cyclic C=N functionalities using whole cells containing *Streptomyces aurantiacus* and *Paenibacillus elgii* B69 gave an e.r. of >99:1 under micro aqueous reaction conditions (Scheme 85).

## 4 Future Directions for Development of More Sustainable Solvents and Processes

The future direction for organic synthesis as a whole will most likely involve a steady transition away from petrochemical-based solvents and unsustainable practices. Dwindling palladium stocks and rising palladium prices (+2170% in 25 years)^400^ are surely challenges that will face us all as chemists in the near future. Even more concerning is the inextricable link between crude oil price and availability with organic solvent and raw material cost, for example, “oil-price shocks”. Surely it is time for us to start to free ourselves from our reliance on petrochemical-derived solvents?

The alternatives will likely comprise a combination of sustainably sourced organic solvents and aqueous–micellar systems. One can envision conventional solvents being retained only for those instances where no viable alternative has yet been found. What is certain at this time is that there are more than enough evidence, case studies, and real world examples to start to promote the early adoption of alternative solvent systems. Sometimes a “drop-in” solvent replacement may be sufficient to migrate away from solvents of concern; other times a solvent mixture may be more appropriate. Research is also being conducted into the identification of new solvents such as 4-MeTHP,^401^ or TMO,^116^ or the creation of brand new solvents such as Cyrene.^122^

Finally, when solvent selection options are exhausted, finding new ways of doing things will be critical for moving forward. Telescoping reaction steps into “one-pot” methods and utilizing cascades, tandem and domino reactions, and multicomponent reactions will surely allow for more efficient synthesis since only one reaction medium is required for multiple steps/transformations.^402^ Late-stage functionalization also represents an enormously powerful technique in opening up more efficient routes to targets or even allowing access to targets that might previously have been impossible to make. However, as has been previously demonstrated, the literature’s over reliance on DCE for these reactions is troublesome, and it is the pioneering work from groups such as Ackermann et al. that will steer the C–H activation community away from unsustainable solvent practices. The utilization of enzymatic catalysis in organic synthesis will also become more commonplace as the gap between biology and chemistry narrows and overlaps, for example, see the recent synthesis of Molnupiravir in a 3-step enzymatic cascade to produce the COVID-19 antiviral in 69% yield. In this process, a combination of aqueous buffered reaction media and 2-MeTHF as extraction solvent shows that a multidisciplinary approach to sustainability may become more commonplace.

The transition away from unsustainable solvent usage practices and processes has already begun, and challenges have been identified and are gradually being overcome. One case study is the adoption of CPME in process chemistry and industry; due to CPME being only available from one supplier (Zeon), supply chain robustness has been a concern. CPME was also absent from the ICH Q3C guidelines for residual solvents in APIs. ICH, or the “International Council For Harmonisation Of Technical Requirements For Pharmaceuticals For Human Use” guidelines are the *de facto* rules for solvent usage and residual solvent levels in API in pharmaceutical manufacturing. Because of this absence in the early days of CPME use, justification for residual levels of CPME would have to be provided, which may have been deemed to be too costly and time-consuming for the potential sustainability improvements achieved.^352^ CPME is now included in the guidance as of 2020/21.^353^ Therefore, it is imperative that regulatory updates and guidance are carried out in a time frame that supports the rapidly progressing developments in greener, more sustainable processes.

Responsibility for training new generations of chemists is also critical for the successful transition toward more sustainable organic synthesis practices. It is no coincidence that the current generation of organic chemists relies so heavily on DMF, THF, and 1,4-dioxane as reaction media, after all it is how we were taught and it became the norm. By introducing safer, more sustainable solvents and synthetic techniques into teaching laboratories and organic synthesis lecture courses, the upcoming generation of chemists stands a chance of adopting these practices and challenging the status quo. Support for these endeavors will also have to come from top levels of organizations to stand a chance of becoming policy and not just personal choice. To quote from Bruce Lipshutz, “the “business as usual” mentality is no longer appropriate”.^236^

## 5 Conclusion

The case for moving away from traditionally employed solvents such as DMF, NMP, and 1,4-dioxane is growing as evidence reinforces the potential hazards that currently employed solvents can inflict on users and the environment. The motivation to move toward safer, greener, more sustainable alternatives to those currently in use has never been clearer. In synthetic organic chemistry, migrating from solvents that carry reproductive toxicity warnings toward those that do not is now a real possibility across a wide range of reaction types.

To assist chemists in industrial and academic settings alike in making more informed solvent selection, we have provided a clear depiction of the current state-of-the-art in the field of potential replacement solvents, their production, and their uses. In addition, the physicochemical properties of a selection of dipolar aprotic and ethereal solvents have been compiled to assist in more judicious solvent selection. Finally, real world examples and case studies where solvents of concern have been successfully replaced have been compiled and presented to reinforce the ideology that solvent moving toward safer, more sustainable alternatives can provide end results that are just as good, if not better, than the current status quo. It is our hope that the information within this review will aid and assist chemists from many disciplines in moving toward a more sustainable future for chemistry as a whole.

## Supporting Information Available

Summarized, printable table of potential solvent replacements for reactions of importance to medicinal and organic chemistry, as discussed in the text; summary and categorization of dipolar aprotic and ethereal solvents by H-phrases (PDF)
Analysis of reaction solvent usage in a variety of organic chemistry journals (XLSX)

## Abbreviations

2-MeTHF: 2-methyltetrahydrofuran
AchE: acetylcholinesterase
API: active pharmaceutical ingredient
CDC: Centre for Disease Control and Prevention
CO(g): carbon monoxide
C*O*_2_(*g*): carbon dioxide
CPME: cyclopentyl methyl ether
DCM: dichloromethane
DIC: *N*,*N*′-diisopropylcarbodiimide
DMAc: *N*,*N*-dimethylacetamide
DMC: dimethylcarbonate
DMF: *N*,*N*′-dimethylformamide
DMI: dimethyl isosorbide
DMSO: dimethyl sulfoxide
dppf: 1,1′-bis(diphenylphosphino)ferrocene
ECHA: European Chemicals Agency
GVL: γ-valerolactone
HMPA: hexamethylphosphoramide
HSP: Hansen solubility parameter
MTBE: methyl *tert*-butyl ether
NBP: *N*-butyl-2-pyrrolidinone
NHC: *N*-heterocyclic carbene
NMP: *N*-methyl-2-pyrrolidinone
NOK: β-sitosterol methoxypolyethylene glycol succinate SPGS-550-M
PC: propylene carbonate
Pd: palladium
PPh_3_: triphenylphosphine
SDS: safety data sheet
S_N_Ar: nucleophilic aromatic substitution
Suc: succinimide
THF: tetrahydrofuran
TMO: 2,2,5,5-tetramethyloxalone
TMTHF: 2,2,5,5-tetramethyltetrahydrofuran
