# Supplementary material for: Replacement of Less-Preferred Dipolar Aprotic and Ethereal Solvents in Synthetic Organic Chemistry with More Sustainable Alternatives
Source: Chem Rev. 2022 Feb 24;122(6):6749–94. doi: 10.1021/acs.chemrev.1c00672 (PMC9098182; doi:10.1021/acs.chemrev.1c00672)
Supplement: Supplementary file 1 — cr1c00672_si_001.pdf [file cr1c00672_si_001.pdf]

Supporting Information

Replacement of Less-Preferred Dipolar Aprotic and Ethereal Solvents in Synthetic Organic Chemistry with More Sustainable Alternatives

Andrew Jordan,[a]* Callum G. J. Hall,[b,c] Lee R. Thorp,[c] Helen F. Sneddon[d]*

[a] School of Chemistry, University of Nottingham, GlaxoSmithKline Carbon Neutral Laboratory, 6 Triumph Road, Nottingham, NG7 2GA, UK. E-mail: andrew.jordan@nottingham.ac.uk

[b] Department of Pure & Applied Chemistry, WestCHEM, University of Strathclyde, Glasgow G1 1XL, Scotland, UK.

[c] GlaxoSmithKline Medicines Research Centre, Gunnels Wood Road, Stevenage, Hertfordshire, SG1 2NY, UK

[d] Green Chemistry Centre of Excellence, University of York, Dept. of Chemistry, University of York, Heslington, York YO10 5DD. E-mail: helen.sneddon@york.ac.uk

Table of Contents

Replacement of Less-Preferred Dipolar Aprotic and Ethereal Solvents in Synthetic Organic Chemistry with More Sustainable Alternatives S1

References S9

Table S1: Unified solvent selection guide for replacement of common dipolar aprotic and ethereal solvents in synthetically useful transformations.

Table S2: Categorization of dipolar aprotic and ethereal solvents by H-phrases

Table S1. Unified solvent selection guide for replacement of common dipolar aprotic solvents in synthetically useful transformations.

1. Amide formation

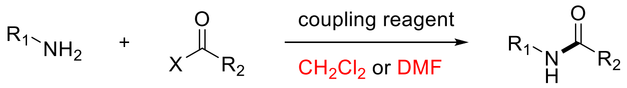

- Suggested solvents, either for reaction of amine with acyl chloride or carboxylic acid in the presence of coupling reagent: **Cyrene.**[1, 2]
- Addition of H_2O may simplify purification by precipitation of amide products.
- Also consider the use of surfactant-water systems such as **PS-750-M** as an alternative to organic solvents. 3
- Water with no surfactant additives can also potentially be used for DIC mediated amide bond formation.[4]
- Direct amidation of unprotected carboxylic acids and amines can be conducted catalytically using 10 mol% $B(OCH_2CF_3)_3$ in *tert*-butyl acetate [5]
- Solvent-Reagent Guide for common amide coupling reagents.[6]

2. Suzuki-Miyaura cross-coupling

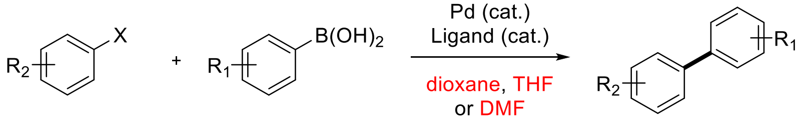

X = I, Br, Cl, OTf

- Suggested solvents: **Cyrene,**[7] **NBP,**[8] **DMI,**[9] or **2-MeTHF.**[10, 11]
- Green solvent selection for Suzuki-Miyaura cross-coupling of amides.[12]
- Sustainable solvents for Suzuki-Miyaura cross-couplings.[13]

* *Early experimental observations suggest Eucalyptol may also be compatible with specific substrate classes.*[14]

3. Mizoroki-Heck cross-coupling

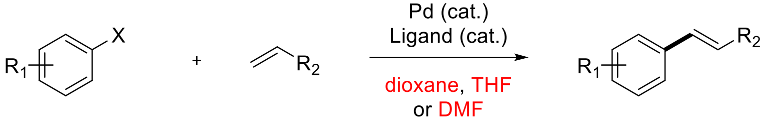

X = I, Br, Cl, OTf

- Suggested solvents: **NBP**,[8] **DMI**,[9] or **propylene carbonate**.[15]
- Comparison of sustainable solvents for Mizoroki-Heck reactions.[15]

4. Sonogashira cross-coupling

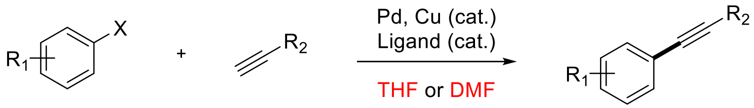

X = I, Br, Cl, OTf

- Suggested solvents: **Cyrene,**[16] **DMI**,[9] **NBP.**[17]
- Other less common pyrrolidinone derivatives such as NOP (N-octyl), NCP (N-cyclohexyl) and NBnP (N-benzyl) may also prove effective.[17]

* *Early experimental observations suggest Eucalyptol may also be compatible with specific substrate classes, with an option of using NEt_3 as a co-solvent in a 1:2/1:3 ratio.*[14]

5. Buchwald-Hartwig amination

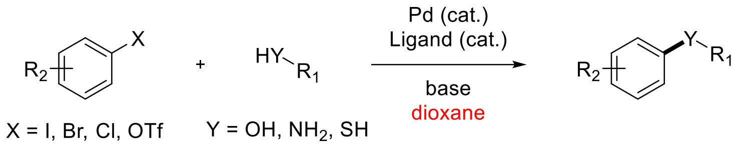

- Suggested solvents: **2-MeTHF**[18] or ***t*BuOH**[19]
- Consider the use of surfactant-water systems such as **TPGS-750-M** as an alternative to organic solvents.[20]
- Where possible, consider performing the transformation with S_NAr conditions to eliminate the use of precious metals as catalysts.[21]
- Solvent Selection Guide for transamidation.[22]

* *Early experimental observations suggest Eucalyptol may also be compatible with specific substrate classes.*[23]

6. Borylation chemistry

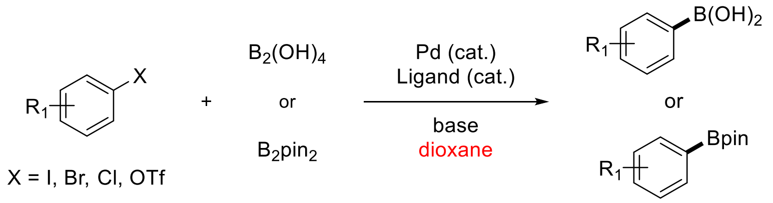

- Suggested solvents: 1:1 mixture of **2-MeTHF** and methanol,[24, 25] **CPME**,[26, 27] **MTBE**[28] or **cyclohexanone**.[29]

7. C-H activation

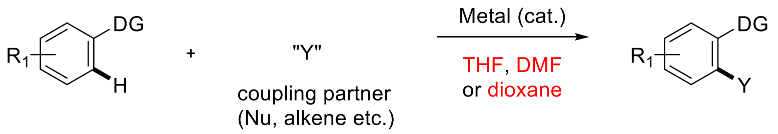

- Suggested solvents: **2-MeTHF**,[30] or **cyclohexanone**.[31]
- PEG-400[32]
- Reviews:
  - 2021 by Dhawa *et al.*[33]
  - 2021 by Dalton *et al.* [34]
  - 2020 by Yu *et al.* [35]
  - 2019 by Gandeepan *et al.*[36]

* Early experimental observations suggest Eucalyptol may also be compatible with specific substrate classes.[14] * Cumene has also been promoted as a potential solvent for C-H bond activation chemistry.[35]

8. Boc deprotection

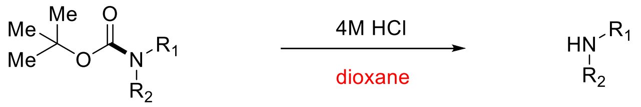

- Suggested solvents: **HCl** in **CPME**,[37] **TFA** in **propylene carbonate**.[38]
- Solutions of HCl in CPME are now commercially available.[39]
- Also consider alternative Boc deprotection conditions, such as the use of NaO^tBu in H_2O/2-MeTHF.[40, 41]

9. Carbonylations & Carboxylations

Carbonylations

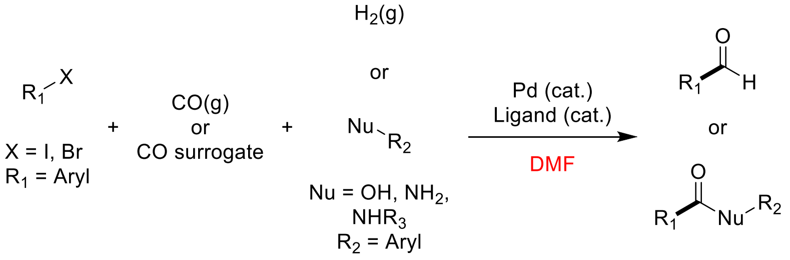

Carboxylations

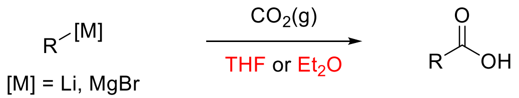

- Suggested solvents for carbonylations: **dimethyl carbonate.**[42, 43]
- Suggested solvents for carboxylations: **2-MeTHF, DMI,**[44] or **dimethyl carbonate.**[43]

11. Organometallic reactions

**Common organometallic reagents**	R_1—MgX	R_2—Li	Boron hydrides	Aluminium hydrides

- Suggested solvents: **2-MeTHF,**[49-51] **CPME**[52]
- A selection of Grignard reagents are commercially available in **2-MeTHF.**[53]

10. Nucleophilic Aromatic Substitution (S_NAr)

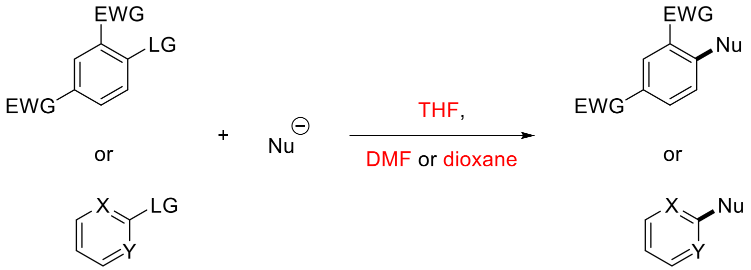

- Suggested solvents: **2-MeTHF.**[45, 46]
- **PEG-400** has also been shown to perform as an effective sustainable solvent for S_NAr reactions of Het-Cl and amines.[47]
- Attempt using metal-free conditions where appropriate.[21]

* *Early experimental observations suggest PolarClean may also be compatible with specific substrate classes.*[48]

12. Urea synthesis

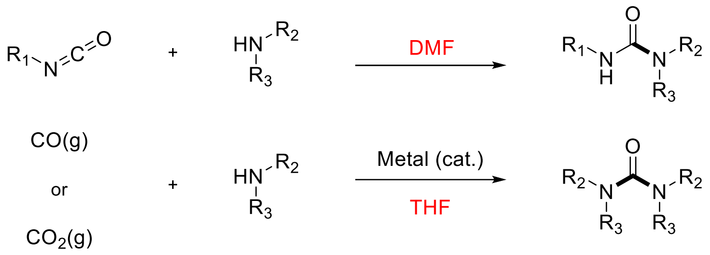

- Suggested solvents: **Cyrene**.[54]
- For the work up, it is recommended to add H_2O to the reaction mixture to induce precipitation of the urea.[54]

13. Solid phase peptide synthesis

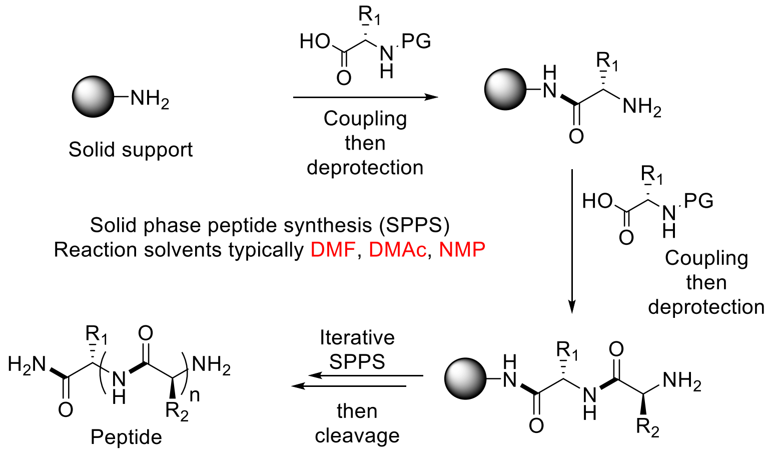

- Suggested solvents: **NBP**,[55] **GVL**.[56, 57]
- Use of a 1:4 mixture of **NBP:EtOAc** has been reported to reduce amounts of HCN formed as a by-product during DIC/Oxyma reactions.[58]
- Use of dimethyl trisulfide (DMTS) as a HCN scrubbing additive can also reduce HCN levels.[58]

* *Early experimental observations suggest GVL may also be compatible with SPPS.*[56, 57]

14. Steglich Esterification

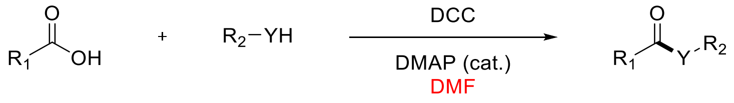

- Reactions utilizing alcohols as the nuclophile can be conducted in **DMC** in conjunction with Mukaiyama's reagent and a suitable tertiary amine base.[59]
- Reactions using phenolic alcohols can also be conducted in water when DIC is used as the coupling reagent.[4]
- Reactions utilizing thiols as the nucleophile can be conducted in **cyclopentanone** in conjunction with T_3P and a suitable tertiary amine base.[60]

Key:

1. **Amide formation**
2. **Suzuki-Miyaura cross-coupling**
3. **Mizoroki-Heck cross-coupling**
4. **Sonogashira cross-coupling**
5. **Buchwald-Hartwig amination**
6. **Borylation chemistry**
7. **C-H activation**
8. **Boc deprotection**
9. **Carbonylations & carboxylations**
10. **S_NAr reactions**
11. **Organometallic reactions**
12. **Solid-phase peptide synthesis**
13. **Urea synthesis**
14. **Steglich Esterification**

***** See main review text for in-depth discussions and further examples.

Disclaimer: Advice provided in this guide in no way represents a risk assessment or chemical compatibility assessment. The author’s take no responsibility for practitioners safety or reaction outcomes. Adequate risk assessment and safety precautions must be taken when planning any chemical process or transformation and are the responsibility of the practitioner.

Table S2. Categorization of dipolar aprotic and ethereal solvents by H-phrases according to the work of McElroy et al.[61] Note: Hazard phrases are those according to the "Harmonized Classification and Labelling" system as approved by the EU. Additional classifications as provided by companies to ECHA in REACH registrations are also included. Exposure limits STEL (Short-term Exposure Limit) and LTEL (Long-term Exposure Limit) are according to ECHA data. *No occupational exposure data available from ECHA. The information provided here does not constitute an alternative to conducting a risk assessment of any solvents used, or sourcing an up to date SDS from your chemical supplier. Environmental impact H-phrases and Thermal Safety/Explosivity H-phrases were omitted for clarity as no red or amber flags were raised for any of the solvents assessed. H340 = May cause genetic defects. H350 = May cause cancer. H351 = Suspected of causing cancer. H360 = May damage fertility or the unborn child.

**Solvent**	**Toxic Red Flag**	**Toxic Amber Flag**	**Long Term toxicity Red Flag**	**Long Term toxicity Amber Flag**	**GHS Pictograms**			**LTEL ppm**	**STEL ppm**	**ECHA Ref**
	H300, H310, H330	H301, H311, H331	H340, H350, H360, H370, H372	H341, H351, H361, H371, H373						
Propylene carbonate					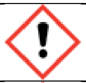			*	*	62
Acetonitrile					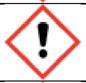	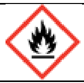		40	*	63
Dimethylsulfoxide (DMSO)					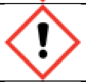			*	*	64
Sulfolane			H360		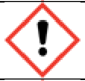			*	*	65
N,N-Dimethylformamide (DMF)			H360		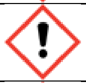		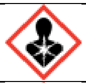	5	10	66
N,N-Dimethylacetamide (DMAc)			H360		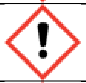		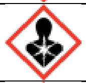	10	20	67
1-Methylpyrrolidin-2-one (NMP)			H360		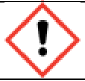		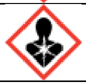	10	20	68
Acetone					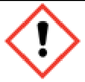	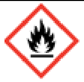		500	*	69
Cyrene					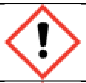			*	*	70
N-Butyl-2-Pyrrolidinone (NBP)					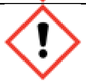			*	*	71

Hexamethylphosphoramide (HMPA)			H340, H350					*	*	72
γ-Valerolactone (GVL)					-	-	-	*	*	73
Cyclohexanone								10	20	74
Cyclopentanone								*	*	75
Dimethyl carbonate								*	*	76
Tetrahydrofuran (THF)				H351				50	100	77
2-Methyl tetrahydrofuran (2-MeTHF)								*	*	78
1,4-Dioxane				H351				20	*	79
MTBE (aka TBME)								50	100	80
Eucalyptol								*	*	81
Diethyl Ether								100	200	82
Cyclopentyl methyl ether (CPME)								*	*	83
Dimethylisosorbide (DMI)					-	-	-	*	*	84
2,2,5,5-Tetramethyloxalone (TMO)								*	*	85
PolarClean								*	*	86

References

(1) Camp, J. E. Bio-available Solvent Cyrene: Synthesis, Derivatization, and Applications. *ChemSusChem* **2018**, *11* (18), 3048-3055. DOI: 10.1002/cssc.201801420.

(2) Wilson, K. L.; Murray, J.; Jamieson, C.; Watson, A. J. B. Cyrene as a bio-based solvent for HATU mediated amide coupling. *Org. Biomol. Chem.* **2018**, *16* (16), 2851-2854, 10.1039/C8OB00653A. DOI: 10.1039/C8OB00653A.

(3) Gabriel, C. M.; Keener, M.; Gallou, F.; Lipshutz, B. H. Amide and Peptide Bond Formation in Water at Room Temperature. *Organic Letters* **2015**, *17* (16), 3968-3971. DOI: 10.1021/acs.orglett.5b01812.

(4) Fattahi, N.; Ayubi, M.; Ramazani, A. Amidation and esterification of carboxylic acids with amines and phenols by N,N′-diisopropylcarbodiimide: A new approach for amide and ester bond formation in water. *Tetrahedron* **2018**, *74* (32), 4351-4356. DOI: https://doi.org/10.1016/j.tet.2018.06.064.

(5) Coomber, C. E.; Laserna, V.; Martin, L. T.; Smith, P. D.; Hailes, H. C.; Porter, M. J.; Sheppard, T. D. Catalytic direct amidations in tert-butyl acetate using $B(OCH_2CF_3)_3$. *Org. Biomol. Chem.* **2019**, *17* (26), 6465-6469, 10.1039/C9OB01012B. DOI: 10.1039/C9OB01012B.

(6) MacMillan, D. S.; Murray, J.; Sneddon, H. F.; Jamieson, C.; Watson, A. J. B. Evaluation of alternative solvents in common amide coupling reactions: replacement of dichloromethane and N,N-dimethylformamide. *Green Chem.* **2013**, *15* (3), 596-600, 10.1039/C2GC36900A. DOI: 10.1039/C2GC36900A.

(7) Wilson, K. L.; Murray, J.; Jamieson, C.; Watson, A. J. B. Cyrene as a Bio-Based Solvent for the Suzuki–Miyaura Cross-Coupling. *Synlett* **2018**, *29* (05), 650-654. DOI: https://dx.doi.org/10.1055/s-0036-1589143.

(8) Sherwood, J.; Parker, H. L.; Moonen, K.; Farmer, T. J.; Hunt, A. J. N-Butylpyrrolidinone as a dipolar aprotic solvent for organic synthesis. *Green Chem.* **2016**, *18* (14), 3990-3996, 10.1039/C6GC00932H. DOI: https://doi.org/10.1039/C6GC00932H.

(9) Wilson, K. L.; Murray, J.; Sneddon, H. F.; Jamieson, C.; Watson, A. J. B. Dimethylisosorbide (DMI) as a Bio-Derived Solvent for Pd-Catalyzed Cross-Coupling Reactions. *Synlett* **2018**, *29* (17), 2293-2297. DOI: https://doi.org/10.1055/s-0037-1611054.

(10) Ramgren, S. D.; Hie, L.; Ye, Y.; Garg, N. K. Nickel-Catalyzed Suzuki–Miyaura Couplings in Green Solvents. *Org. Lett.* **2013**, *15* (15), 3950-3953. DOI: 10.1021/ol401727y.

(11) Lei, P.; Ling, Y.; An, J.; Nolan, S. P.; Szostak, M. 2-Methyltetrahydrofuran (2-MeTHF): A Green Solvent for Pd−NHC-Catalyzed Amide and Ester Suzuki-Miyaura Cross-Coupling by N−C/O−C Cleavage. *Adv. Synth. Catal.* **2019**, *361* (24), 5654-5660. DOI: 10.1002/adsc.201901188.

(12) Lei, P.; Mu, Y.; Wang, Y.; Wang, Y.; Ma, Z.; Feng, J.; Liu, X.; Szostak, M. Green Solvent Selection for Suzuki–Miyaura Coupling of Amides. *ACS Sustainable Chem. Eng.* **2021**, *9* (1), 552-559. DOI: 10.1021/acssuschemeng.0c08044.

(13) M. Yousaf; A. F. Zahoor; R. Akhtar; M. Ahmad; Naheed, S. *Mol Divers.* **2020**, *24*, 821-839.

(14) Campos, J. F.; Scherrmann, M.-C.; Berteina-Raboin, S. Eucalyptol: a new solvent for the synthesis of heterocycles containing oxygen, sulfur and nitrogen. *Green Chem.* **2019**, *21* (6), 1531-1539, 10.1039/C8GC04016H. DOI: 10.1039/C8GC04016H.

(15) Parker, H. L.; Sherwood, J.; Hunt, A. J.; Clark, J. H. Cyclic Carbonates as Green Alternative Solvents for the Heck Reaction. *ACS Sustain. Chem. Eng.* **2014**, *2* (7), 1739-1742. DOI: 10.1021/sc5002287.

(16) Wilson, K. L.; Kennedy, A. R.; Murray, J.; Greatrex, B.; Jamieson, C.; Watson, A. J. B. Scope and limitations of a DMF bio-alternative within Sonogashira cross-coupling and Cacchi-type annulation. *Beilstein J. Org. Chem.* **2016**, *12*, 2005-2011. DOI: 10.3762/bjoc.12.187 PubMed.

(17) Ferrazzano, L.; Martelli, G.; Fantoni, T.; Daka, A.; Corbisiero, D.; Viola, A.; Ricci, A.; Cabri, W.; Tolomelli, A. Fast Heck–Cassar–Sonogashira (HCS) Reactions in Green Solvents. *Org. Lett.* **2020**, *22* (10), 3969-3973. DOI: 10.1021/acs.orglett.0c01269.

(18) Bindu, B.; Vijayalakshmi, S.; Manikandan, A. Synthesis and discovery of triazolo-pyridazine-6-yl-substituted piperazines as effective anti-diabetic drugs; evaluated over dipeptidyl peptidase-4 inhibition mechanism and insulinotropic activities. *Eur. J. Med. Chem.* **2020**, *187*, 111912. DOI: https://doi.org/10.1016/j.ejmech.2019.111912.

(19) Ma, F.; Xie, X.; Ding, L.; Gao, J.; Zhang, Z. Palladium-catalyzed coupling reaction of amino acids (esters) with aryl bromides and chlorides. *Tetrahedron* **2011**, *67* (48), 9405-9410. DOI: https://doi.org/10.1016/j.tet.2011.09.109.

(20) Lipshutz, B. H.; Ghorai, S.; Abela, A. R.; Moser, R.; Nishikata, T.; Duplais, C.; Krasovskiy, A.; Gaston, R. D.; Gadwood, R. C. TPGS-750-M: A Second-Generation Amphiphile for Metal-Catalyzed Cross-Couplings in Water at Room Temperature. *J. Org. Chem.* **2011**, *76* (11), 4379-4391. DOI: 10.1021/jo101974u.

(21) Walsh, K.; Sneddon, H. F.; Moody, C. J. Amination of Heteroaryl Chlorides: Palladium Catalysis or SNAr in Green Solvents? *ChemSusChem* **2013**, *6* (8), 1455-1460. DOI: https://doi.org/10.1002/cssc.201300239.

(22) Lei, P.; Wang, Y.; Mu, Y.; Wang, Y.; Ma, Z.; Feng, J.; Liu, X.; Szostak, M. Green-Solvent Selection for Acyl Buchwald–Hartwig Cross-Coupling of Amides (Transamidation). *ACS Sustainable Chem. Eng.* **2021**, *9* (44), 14937-14945. DOI: 10.1021/acssuschemeng.1c05307.

(23) Campos, J. F.; Berteina-Raboin, S. Eucalyptol as a Bio-Based Solvent for Buchwald-Hartwig Reaction on O,S,N-Heterocycles. *Catalysts* **2019**, *9* (10), 840.

(24) Reilly, M. K.; Rychnovsky, S. D. DABO Boronates: Stable Heterocyclic Boronic Acid Complexes for Use in Suzuki-Miyaura Cross-Coupling Reactions. *Synlett* **2011**, *2011* (16), 2392-2396. DOI: 10.1055/s-0030-1261218

(25) Wang, X.; Liu, W.-G.; Tung, C.-H.; Wu, L.-Z.; Cong, H. A Monophosphine Ligand Derived from Anthracene Photodimer: Synthetic Applications for Palladium-Catalyzed Coupling Reactions. *Org. Lett.* **2019**, *21* (20), 8158-8163. DOI: 10.1021/acs.orglett.9b02414.

(26) Zhang, M.; Yao, Y.; Stang, P. J.; Zhao, W. Divergent and Stereoselective Synthesis of Tetraarylethylenes from Vinylboronates. *Angew. Chem. Int. Ed.* **2020**, *59* (45), 20090-20098. DOI: https://doi.org/10.1002/anie.202008113.

(27) Szadkowska, A.; Pawłowski, R.; Zaorska, E.; Staszko, S.; Trzybiński, D.; Woźniak, K. NHC copper complexes functionalized with sulfoxide and sulfone moieties. *Appl Organometal Chem.* **2019**, *33* (8), e4983. DOI: https://doi.org/10.1002/aoc.4983.

(28) Harrisson, P.; Morris, J.; Steel, P. G.; Marder, T. B. A One-Pot, Single-Solvent Process for Tandem, Catalyzed C-H Borylation-Suzuki-Miyaura Cross-Coupling Sequences. *Synlett* **2009**, *2009* (01), 147-150.

(29) Taher, E. S.; Guest, P.; Benton, A.; Ma, X.; Banwell, M. G.; Willis, A. C.; Seiser, T.; Newton, T. W.; Hutzler, J. The Synthesis of Certain Phomentrioloxin A Analogues and Their Evaluation as Herbicidal Agents. *J. Org. Chem.* **2017**, *82* (1), 211-233. DOI: 10.1021/acs.joc.6b02372.

(30) Messinis, A. M.; Finger, L. H.; Hu, L.; Ackermann, L. Allenes for Versatile Iron-Catalyzed C–H Activation by Weak O-Coordination: Mechanistic Insights by Kinetics, Intermediate Isolation, and Computation. *J. Am. Chem. Soc.* **2020**, *142* (30), 13102-13111. DOI: 10.1021/jacs.0c04837.

(31) Suzuki, I.; Kondo, H.; Kochi, T.; Kakiuchi, F. Selective Monoarylation of Aromatic Ketones via C–H Bond Cleavage by Trialkylphosphine Ruthenium Catalysts. *J. Org. Chem.* **2019**, *84* (20), 12975-12982. DOI: 10.1021/acs.joc.9b01756.

(32) Reddy, G. C.; Balasubramanyam, P.; Salvanna, N.; Das, B. Copper-Mediated C–H Activation of 1,3,4-Oxadiazoles with 1,1-Dibromo-1-alkenes Using PEG-400 as a Solvent Medium: Distinct Approach for the Alkynylation of 1,3,4-Oxadiazoles. *Eur. J. Org. Chem.* **2012**, *2012* (3), 471-474. DOI: https://doi.org/10.1002/ejoc.201101542.

(33) Dhawa, U.; Kaplaneris, N.; Ackermann, L. Green strategies for transition metal-catalyzed C–H activation in molecular syntheses. *Organic Chemistry Frontiers* **2021**, *8* (17), 4886-4913, 10.1039/D1QO00727K. DOI: 10.1039/D1QO00727K.

(34) Dalton, T.; Faber, T.; Glorius, F. C–H Activation: Toward Sustainability and Applications. *ACS Cent. Sci.* **2021**, *7* (2), 245-261. DOI: 10.1021/acscentsci.0c01413.

(35) Yu, C.; Sanjosé-Orduna, J.; Patureau, F. W.; Pérez-Temprano, M. H. Emerging unconventional organic solvents for C–H bond and related functionalization reactions. *Chem. Soc. Rev.* **2020**, *49* (6), 1643-1652, 10.1039/C8CS00883C. DOI: 10.1039/C8CS00883C.

(36) Gandeepan, P.; Kaplaneris, N.; Santoro, S.; Vaccaro, L.; Ackermann, L. Biomass-Derived Solvents for Sustainable Transition Metal-Catalyzed C–H Activation. *ACS Sustainable Chem. Eng.* **2019**, *7* (9), 8023-8040. DOI: 10.1021/acssuschemeng.9b00226.

(37) Watanabe, K.; Kogoshi, N.; Miki, H.; Torisawa, Y. Improved Pinner Reaction with CPME as a Solvent. *Synth. Commun* **2009**, *39* (11), 2008-2013. DOI: 10.1080/00397910802632548.

(38) Lawrenson, S. B.; Arav, R.; North, M. The greening of peptide synthesis. *Green Chem.* **2017**, *19* (7), 1685-1691, 10.1039/C7GC00247E. DOI: 10.1039/C7GC00247E.

(39) *3M HCl in CPME available from Sigma Aldrich*. https://www.sigmaaldrich.com/catalog/product/aldrich/705551?lang=en&region=GB (accessed.

(40) Aycock, D. F. Solvent Applications of 2-Methyltetrahydrofuran in Organometallic and Biphasic Reactions. *Org. Process Res. Dev* **2007**, *11* (1), 156-159. DOI: 10.1021/op060155c.

(41) Tom, N. J.; Ripin, D. B.; Castaldi, M. J. Processes for the preparation of benzoimidazole derivatives. US 20050020625, 2005.

(42) de Albuquerque, D. Y.; de Moraes, J. R.; Schwab, R. S. Palladium-Catalyzed Aminocarbonylation Reaction to Access 1,2,3-Triazole-5-carboxamides Using Dimethyl Carbonate as Sustainable Solvent. *Eur. J. Org. Chem.* **2019**, *2019* (39), 6673-6681. DOI: https://doi.org/10.1002/ejoc.201901249.

(43) Ismael, A.; Gevorgyan, A.; Skrydstrup, T.; Bayer, A. Renewable Solvents for Palladium-Catalyzed Carbonylation Reactions. *Organic Process Research & Development* **2020**, *24* (11), 2665-2675. DOI: 10.1021/acs.oprd.0c00325.

(44) Gevorgyan, A.; Hopmann, K. H.; Bayer, A. Exploration of New Biomass-Derived Solvents: Application to Carboxylation Reactions. *ChemSusChem* **2020**, *13* (8), 2080-2088. DOI: 10.1002/cssc.201903224.

(45) Tao, Y.; Keene, N. F.; Wiglesworth, K. E.; Sitter, B.; McWilliams, J. C. Early Process Development of an Irreversible Epidermal Growth Factor Receptor (EGFR) T790 M Inhibitor. *Org. Process Res. Dev.* **2019**, *23* (3), 382-388. DOI: 10.1021/acs.oprd.8b00437.

(46) Smith, S. M.; Buchwald, S. L. Regioselective 2-Amination of Polychloropyrimidines. *Org. Lett.* **2016**, *18* (9), 2180-2183. DOI: 10.1021/acs.orglett.6b00799.

(47) Campos, J. F.; Loubidi, M.; Scherrmann, M.-C.; Berteina-Raboin, S. A Greener and Efficient Method for Nucleophilic Aromatic Substitution of Nitrogen-Containing Fused Heterocycles. *Molecules* **2018**, *23* (3), 684-699.

(48) Cseri, L.; Szekely, G. Towards cleaner PolarClean: efficient synthesis and extended applications of the polar aprotic solvent methyl 5-(dimethylamino)-2-methyl-5-oxopentanoate. *Green Chem.* **2019**, *21* (15), 4178-4188, 10.1039/C9GC01958H. DOI: 10.1039/C9GC01958H.

(49) De Angelis, S.; De Renzo, M.; Carlucci, C.; Degennaro, L.; Luisi, R. A convenient enantioselective CBS-reduction of arylketones in flow-microreactor systems. *Org. Biomol. Chem.* **2016**, *14* (18), 4304-4311, 10.1039/C6OB00336B. DOI: 10.1039/C6OB00336B.

(50) Pace, V.; de la Vega-Hernández, K.; Urban, E.; Langer, T. Chemoselective Schwartz Reagent Mediated Reduction of Isocyanates to Formamides. *Org. Lett.* **2016**, *18* (11), 2750-2753. DOI: 10.1021/acs.orglett.6b01226.

(51) Pace, V.; Hoyos, P.; Castoldi, L.; Domínguez de María, P.; Alcántara, A. R. 2-Methyltetrahydrofuran (2-MeTHF): A Biomass-Derived Solvent with Broad Application in Organic Chemistry. *ChemSusChem* **2012**, *5* (8), 1369-1379. DOI: 10.1002/cssc.201100780.

(52) Kobayashi, S.; Shibukawa, K.; Miyaguchi, Y.; Masuyama, A. Grignard Reactions in Cyclopentyl Methyl Ether. *Asian Journal of Organic Chemistry* **2016**, *5* (5), 636-645. DOI: https://doi.org/10.1002/ajoc.201600059.

(53) *Commercially available reagent preparations available as 2-MeTHF solutions from Sigma-Aldrich*. https://www.sigmaaldrich.com/catalog/search?term=2-methf&interface=All&N=0&mode=match%20partialmax&lang=en&region=GB&focus=product (accessed 30/07/2020).

(54) Mistry, L.; Mapesa, K.; Bousfield, T. W.; Camp, J. E. Synthesis of ureas in the bio-alternative solvent Cyrene. *Green Chem.* **2017**, *19* (9), 2123-2128, Article. DOI: 10.1039/c7gc00908a.

(55) Lopez, J.; Pletscher, S.; Aemissegger, A.; Bucher, C.; Gallou, F. N-Butylpyrrolidinone as Alternative Solvent for Solid-Phase Peptide Synthesis. *Org. Process Res. Dev* **2018**, *22* (4), 494-503. DOI: https://doi.org/10.1021/acs.oprd.7b00389.

(56) Kumar, A.; Jad, Y. E.; El-Faham, A.; de la Torre, B. G.; Albericio, F. Green solid-phase peptide synthesis 4. γ-Valerolactone and N-formylmorpholine as green solvents for solid phase peptide synthesis. *Tetrahedron Lett.* **2017**, *58* (30), 2986-2988. DOI: https://doi.org/10.1016/j.tetlet.2017.06.058.

(57) Al Musaimi, O.; El-Faham, A.; Basso, A.; de la Torre, B. G.; Albericio, F. γ-Valerolactone (GVL): An eco-friendly anchoring solvent for solid-phase peptide synthesis. *Tetrahedron Lett.* **2019**, *60* (38), 151058. DOI: https://doi.org/10.1016/j.tetlet.2019.151058.

(58) Erny, M.; Lundqvist, M.; Rasmussen, J. H.; Ludemann-Hombourger, O.; Bihel, F.; Pawlas, J. Minimizing HCN in DIC/Oxyma-Mediated Amide Bond-Forming Reactions. *Organic Process Research & Development* **2020**, *24* (7), 1341-1349. DOI: 10.1021/acs.oprd.0c00227.

(59) Jordan, A.; Sneddon, H. F.; Whymark, K. D.; Sydenham, J. A Solvent-Reagent Selection Guide for Steglich-type Esterification of Carboxylic Acids. *Green Chem.* **2021**, *10.1039/D1GC02251B*, 10.1039/D1GC02251B. DOI: 10.1039/D1GC02251B.

(60) Jordan, A.; Sneddon, H. F. Development of a solvent-reagent selection guide for the formation of thioesters. *Green Chem.* **2019**, *21* (8), 1900-1906, 10.1039/C9GC00355J. DOI: 10.1039/C9GC00355J.

(61) McElroy, C. R.; Constantinou, A.; Jones, L. C.; Summerton, L.; Clark, J. H. Towards a holistic approach to metrics for the 21st century pharmaceutical industry. *Green Chem.* **2015**, *17* (5), 3111-3121, 10.1039/C5GC00340G. DOI: 10.1039/C5GC00340G.

(62) *Propylene Carbonate ECHA*. https://echa.europa.eu/substance-information/-/substanceinfo/100.003.248 (accessed 07/06/2021).

(63) *Acetonitrile ECHA*. https://echa.europa.eu/substance-information/-/substanceinfo/100.000.760 (accessed 07/06/2021).

(64) *DMSO ECHA*. https://echa.europa.eu/substance-information/-/substanceinfo/100.000.604 (accessed 07/06/2021).

(65) *Sulfolane ECHA*. https://echa.europa.eu/substance-information/-/substanceinfo/100.004.349 (accessed 07/06/2021).

(66) *DMF ECHA*. https://echa.europa.eu/substance-information/-/substanceinfo/100.000.617 (accessed 07/06/2021).

(67) *DMAc ECHA*. https://echa.europa.eu/substance-information/-/substanceinfo/100.004.389 (accessed 07/06/2021).

(68) *NMP ECHA*. https://echa.europa.eu/substance-information/-/substanceinfo/100.011.662 (accessed 07/06/2021).

(69) *Acetone ECHA*. https://echa.europa.eu/substance-information/-/substanceinfo/100.000.602 (accessed 07/06/2021).

(70) *Cyrene ECHA*. https://echa.europa.eu/substance-information/-/substanceinfo/100.234.612 (accessed 07/06/2021).

(71) *NBP ECHA*. https://echa.europa.eu/substance-information/-/substanceinfo/100.020.399 (accessed 07/06/2021).

(72) *HMPA ECHA*. https://echa.europa.eu/substance-information/-/substanceinfo/100.010.595 (accessed 07/06/2021).

(73) *GVL ECHA*. https://echa.europa.eu/substance-information/-/substanceinfo/100.003.245 (accessed 07/06/2021).

(74) *Cyclohexanone ECHA*. https://echa.europa.eu/substance-information/-/substanceinfo/100.003.302 (accessed 07/06/2021).

(75) *Cyclopentanone ECHA*. https://echa.europa.eu/substance-information/-/substanceinfo/100.004.033 (accessed 07/06/2021).

(76) *DMC ECHA*. https://echa.europa.eu/substance-information/-/substanceinfo/100.009.527 (accessed 07/06/2021).

(77) *THF ECHA*. https://echa.europa.eu/substance-information/-/substanceinfo/100.003.389 (accessed 07/06/2021).

(78) *2-MeTHF ECHA*. https://echa.europa.eu/substance-information/-/substanceinfo/100.002.281 (accessed 07/06/2021).

(79) *1,4-Dioxane ECHA*. https://echa.europa.eu/substance-information/-/substanceinfo/100.004.239 (accessed 07/06/2021).

(80) *TBME ECHA*. https://echa.europa.eu/substance-information/-/substanceinfo/100.015.140 (accessed 23/01/2022).

(81) *Eucalyptol ECHA*. https://echa.europa.eu/substance-information/-/substanceinfo/100.006.757 (accessed 07/06/2021).

(82) *Diethyl Ether ECHA*. https://echa.europa.eu/substance-information/-/substanceinfo/100.000.425 (accessed 23/01/2022).

(83) *CPME ECHA*. https://echa.europa.eu/substance-information/-/substanceinfo/100.104.006 (accessed 07/06/2021).

(84) *DMI ECHA*. https://echa.europa.eu/substance-information/-/substanceinfo/100.023.782 (accessed 07/06/2021).

(85) *TMO ECHA*. https://echa.europa.eu/substance-information/-/substanceinfo/100.035.547 (accessed 07/06/2021).

(86) *PolarClean ECHA*. https://echa.europa.eu/substance-information/-/substanceinfo/100.148.956 (accessed 07/06/2021).
